# From Crypts to Cancer: A Holistic Perspective on Colorectal Carcinogenesis and Therapeutic Strategies

**DOI:** 10.3390/ijms25179463

**Published:** 2024-08-30

**Authors:** Ehsan Gharib, Gilles A. Robichaud

**Affiliations:** 1 Département de Chimie et Biochimie, Université de Moncton, Moncton, NB E1A 3E9, Canada; 2 Atlantic Cancer Research Institute, Moncton, NB E1C 8X3, Canada

**Keywords:** colorectal cancer, epidemiology, molecular pathogenesis, risk factor, therapeutic strategy

## Abstract

Colorectal cancer (CRC) represents a significant global health burden, with high incidence and mortality rates worldwide. Recent progress in research highlights the distinct clinical and molecular characteristics of colon versus rectal cancers, underscoring tumor location’s importance in treatment approaches. This article provides a comprehensive review of our current understanding of CRC epidemiology, risk factors, molecular pathogenesis, and management strategies. We also present the intricate cellular architecture of colonic crypts and their roles in intestinal homeostasis. Colorectal carcinogenesis multistep processes are also described, covering the conventional adenoma–carcinoma sequence, alternative serrated pathways, and the influential Vogelstein model, which proposes sequential *APC*, *KRAS*, and *TP53* alterations as drivers. The consensus molecular CRC subtypes (CMS1-CMS4) are examined, shedding light on disease heterogeneity and personalized therapy implications.

## 1. Introduction

Over the past decade, significant advances have been made to our understanding of cellular and molecular processes supporting CRC pathogenesis. This has been largely due to experimental animal models, patient-derived resources, and advanced techniques (i.e., organoids/xenografts). These advances have provided invaluable contributions to decipher intratumoral heterogeneity and cancer subtype nomenclature. Globally, this knowledge has paved the way to current screening, prevention programs, and intervention (i.e., surgical, chemotherapeutic, and emerging targeted/immunotherapy) management strategies. The following Sections of this review will encompass current knowledge on CRC biology, pathogenesis, and mechanistic networks while emphasizing strategic approaches and ongoing research for improving patient outcome and personalized therapy.

### 1.1. Colorectal Cancer around the World: Burden, Risks, and Management

Colorectal cancer (CRC) poses a major global health challenge [1]. It is one of the most commonly diagnosed cancers worldwide and a leading cause of cancer-related mortality internationally [1,2]. In 2022, CRC accounted for over 1.9 million new cases and 904,019 deaths globally according to estimates from the Global Cancer Observatory (GLOBOCAN) [3]. As shown in Table 1, the incidence and mortality rates vary substantially between world regions where developed nations report higher CRC burden [3].

Various lifestyle and hereditary risk factors influence CRC development [4,5,6,7,8,9,10,11]. Age is the predominant risk factor, as the majority of cases are diagnosed after the age of 60 [4]. Compounding behavioral risks include smoking, heavy alcohol use, physical inactivity, and diets rich in red/processed meats [5,6]. Medical risks encompass family history of CRC [7] or adenomatous polyps [8,10], personal history of inflammatory bowel disease [9], and genetic syndromes like familial adenomatous polyposis (FAP) [11]. In fact, it is estimated that up to 20% of CRC may be attributed to modifiable lifestyle habits [5,12].

Screening has shown to play a critical role in mitigating CRC burden [13,14]. It allows for early detection of precancerous polyps which can be removed before developing into cancer [13,14]. Regular screening starting at age 50 is recommended for average-risk individuals by major health organizations [15]. Common screening tests include colonoscopy, fecal immunochemical testing, and flexible sigmoidoscopy [16,17,18]. Screening guidelines may start earlier or occur more frequently for those with elevated familial or medical risks [19].

Treatment options depend on the cancer stage, location, and patient risk factors. Surgical resection remains the mainstay for early-stage colon and rectal cancers [20,21]. Advanced cases may require more extensive surgeries like colectomies [22]. Chemotherapy regimens following or preceding surgery are standard for intermediate and high-risk patients [23]. Targeting drug and immunotherapies are being increasingly utilized as well [24,25]. However, radiation therapy has proven worthy mainly for rectal cancers [26]. Palliative care is still common practice and aims to improve quality of life for metastatic cases [27,28]. Studies show that lifelong surveillance after primary treatment greatly reduces recurrence risks [7,29]. Overall, implementation of organized screening programs coupled with multidisciplinary management approaches have led to declining CRC mortality in developed nations over the past few decades [30,31,32].

### 1.2. Structure and Function of Colon Crypts in Health and CRC Disease

The normal colon epithelium consists of structures called crypts of Lieberkühn or colonic crypts [33]. These crypts are small invaginations or pits located in the lining of the colon (large intestine) [34]. They play a vital role in the functioning of the colon [35,36,37,38]. The cells within the crypts are responsible for various functions, including absorption, secretion, and protection of the colon [35]. The main types of cells found in colonic crypts include absorptive cells (enterocytes), goblet cells, enteroendocrine cells, Paneth cells, and stem cells [39,40]. Enterocytes are the most abundant cells in the colon epithelium [41]. They have microvilli on their surface, which increase the surface area for absorption of water, electrolytes, and nutrients [42]. The goblet cells are specialized cells secrete mucus, which helps lubricate the intestinal lining and protects it from the abrasive action of fecal matter [43]. They are responsible for the production of mucus that forms the protective mucus layer in the colon [44]. Enteroendocrine cells produce and release various hormones into the bloodstream [45]. These hormones play a role in regulating digestion, nutrient absorption, and other physiological processes [36]. Paneth cells are primarily found in the base of the crypts in the small intestine, but they can also be present in the crypts of the colon [46]. Paneth cells secrete antimicrobial peptides, enzymes, and growth factors that help maintain the intestinal barrier and protect against pathogens [37]. Stem cells are found at the bottom of the colonic crypts and continuously divide and differentiate to replace the cells that are shed from the surface of the colon [38]. These stem cells are crucial for the regeneration and maintenance of the colonic epithelium [47]. These different cell types work together to maintain the normal structure and function of the colon epithelium, ensuring proper absorption, secretion, and protection of the colon [35,36,37,38,44].

### 1.3. Deciphering Colon and Rectal Cancers: Location-Specific Differences in Behavior and Management

The location of cancer along the colon and rectum tract can have a significant impact on clinical outcomes and drug responsiveness [48]. Given that the colon and rectum are distinct anatomical tissues of the gastrointestinal tract, they are characterized with unique physiological features [49]. As a result, tumors that arise in these regions exhibit differences in behavior (i.e., aggression and malignancy) and response to treatment [50]. As a result, metastasis and disease progression represent key factors contributing to the variability in colon and rectum cancers clinical outcomes [51].

Interestingly, tumors located in the right side of the colon (ascending colon and cecum) tend to have a different biological behavior compared to those in the left side (descending colon, sigmoid colon, and rectum) [52]. This difference can be attributed to several factors beyond just anatomical variations. The right and left colon have distinct embryological origins (midgut and hindgut, respectively), leading to different genetic and epigenetic profiles [53]. Additionally, the microbiome composition and mucosal immune environment vary between the two sides, influencing tumor development and progression [54,55,56]. Specifically, right-sided colon cancers are often only detected in later stages and have a higher likelihood of metastasis at the time of diagnosis [57]. They also tend to be associated with worse prognostic features, such as poorly differentiated tumors [58] and characterized with specific genetic alterations such as BRAF mutations [59]. Right-sided tumors are more likely to be microsatellite instability-high (MSI-H) and have the CpG island methylator phenotype (CIMP) [60,61], while left-sided tumors more frequently exhibit chromosomal instability (CIN) and mutations in genes like *APC*, *KRAS*, and *TP53* [60,62,63]. In addition, evidence suggests that tumors in different locations of the colon and rectum differentially respond to drug therapies [41,64]. This may be partly due to the distinct metabolic functions and gene expression patterns observed between the right and left colon [65,66]. For example, cancers arising in the right side of the colon have been found to be less responsive to certain targeted therapies, such as anti-epidermal growth factor receptor (EGFR) antibodies (i.e., cetuximab and panitumumab) [67,68]. On the other hand, these therapeutic approaches show better efficacy in treating tumors located in the left side of the colon and rectum [69]. The varying exposure to carcinogens due to differences in transit time between the right and left colon may also contribute to these distinct biological behaviors and treatment responses [70].

Rectal cancers are located in the lower part of the large intestine, near the anus [71]. Due to its proximity to other organs and structures, such as the sphincter muscles and the pelvic bones, the surgical removal of rectal tumors can be challenging [72]. In order to facilitate successful surgery and improve patient outcome, neoadjuvant therapy is often used for rectal cancers [73]. Neoadjuvant therapy involves administering chemotherapy and radiation therapy prior to surgery to shrink the tumor and reduce the risk of recurrence [74]. This approach helps to increase the chances of complete tumor removal and preserve the sphincter function, which is crucial for bowel control [75]. On the other hand, colon cancers, which are located in the upper part of the large intestine, present more space and are relatively more accessible for surgical intervention [76,77]. Although neoadjuvant therapy is less commonly used for colon cancers [78], chemotherapy may be administered in some cases after surgery (adjuvant therapy) to help destroy any remaining cancer cells and reduce the risk of recurrence [79]. Nevertheless, it is important to note that treatment approaches always vary based on the cancer profile and individual patient factors [80]. The decision on the most appropriate treatment plan for a specific patient is typically made by a multidisciplinary team of healthcare professionals, including surgeons, oncologists, and radiologists, taking into account various factors, including tumor location, staging, and patient-specific characteristics (e.g., age and health) [81].

## 2. CRC Development through Genetic and Epigenetic Changes

CRC development is a complex process that involves the accumulation of genetic and epigenetic alterations in the cells lining the colon or rectum [82]. While there are various pathways through which CRC can develop, the most common pathway involves the progression from an aberrant crypt to a benign adenomatous polyp and ultimately to sporadic CRC [82]. The process begins with the formation of an aberrant crypt, which is an abnormal glandular structure within the lining of the colon or rectum [83]. Aberrant crypts can arise due to genetic mutations or environmental factors that lead to cellular changes [84]. Over time, some of these aberrant crypts can grow and develop into benign adenomatous polyps [84]. Adenomatous polyps are characterized by the presence of dysplastic or abnormal cells [85]. These polyps have the potential to progress further and become cancerous if left untreated [86]. The transformation from a benign adenomatous polyp to sporadic CRC involves the accumulation of additional genetic and epigenetic alterations, which can disrupt normal cellular function and lead to uncontrolled cell growth [51]. Nevertheless, it is important to note that not all adenomatous polyps will progress to cancer [87]. However, certain factors such as the size, number, and degree of dysplasia of the polyps can increase the risk of progression to CRC [88]. Other factors, such as family history, inflammatory bowel disease, and lifestyle choices like smoking and diet, can also contribute to the development of sporadic CRC [89,90]. The understanding of the progression from aberrant crypts to benign adenomatous polyps and eventually to sporadic CRC has helped in the development of screening and prevention strategies [14]. Regular screening tests, such as colonoscopies, can detect and remove adenomatous polyps before they become cancerous, thus reducing the risk of developing CRC [91].

### 2.1. The Conventional Adenoma–Carcinoma Sequence Model of CRC Progression

**Figure 1:**
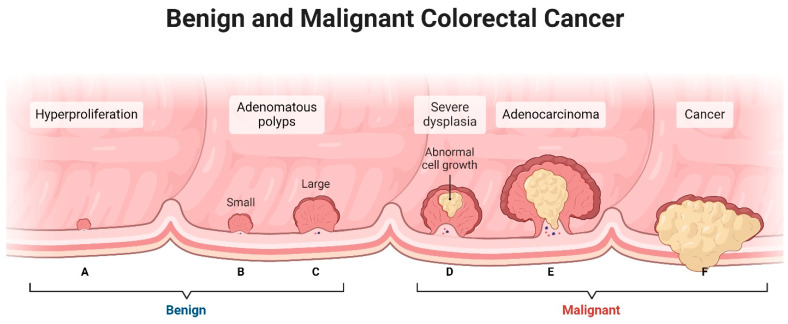
**Conventional adenoma–carcinoma sequence pathway of colorectal cancer development.** (**A**) The first stage involves hyperproliferation, where there is an increased rate of cell division and growth within the colonic epithelium. This is depicted as a slight thickening of the epithelial lining. (**B**) As the process progresses, small adenomatous polyps begin to form. These polyps are benign growths protruding into the colonic lumen and are represented as small, spherical structures attached to the epithelial lining. (**C**) Over time, some of these small polyps can grow larger, forming large adenomatous polyps. These are shown as larger spherical masses connected to the colonic wall. (**D**) Within these larger polyps, severe dysplasia occurs, characterized by abnormal cell growth and organization. This stage is visually depicted by the presence of an irregular, yellow-colored growth within the polyp structure. (**E**) The dysplastic cells in the polyp can then transform into an adenocarcinoma, which is an invasive malignant tumor. This stage is represented by a large, irregularly shaped mass protruding into the colonic lumen, with a distinct boundary separating it from the surrounding normal tissue. (**F**) In the final stage, the adenocarcinoma has progressed to a full-blown colorectal cancer. This is depicted as a large, irregular mass filling a significant portion of the colonic lumen, indicating advanced tumor growth and invasion. Figure created using BioRender (https://www.biorender.com/ accessed on 29 July 2024).

The conventional adenoma–carcinoma sequence is a widely accepted model that explains the development of CRC from benign polyps called adenomas [92]. It describes a stepwise progression from normal colorectal tissue to adenomas and ultimately to invasive carcinoma (Figure 1) [92]. The process begins with the normal lining of the colon or rectum [93]. Genetic mutations occur within the cells of the colorectal tissue [94]. These mutations are often acquired due to various factors such as environmental exposures (i.e., diet, smoking) or inherited genetic predispositions [95]. The initiated cells begin to undergo abnormal growth and form benign polyps called adenomas which can vary in size and shape [96]. Over time, some adenomas may progress to a stage called dysplasia [97]. Dysplasia refers to the presence of abnormal cells within the adenoma, which have acquired additional genetic alterations [98]. High-grade dysplasia indicates a more advanced stage with a greater likelihood of becoming cancerous [99]. If left untreated, some dysplastic adenomas can transform into invasive carcinoma, where the abnormal cells invade through the layers of the colon or rectum and potentially spread to nearby lymph nodes or distant organs [100].

**Figure 2:**
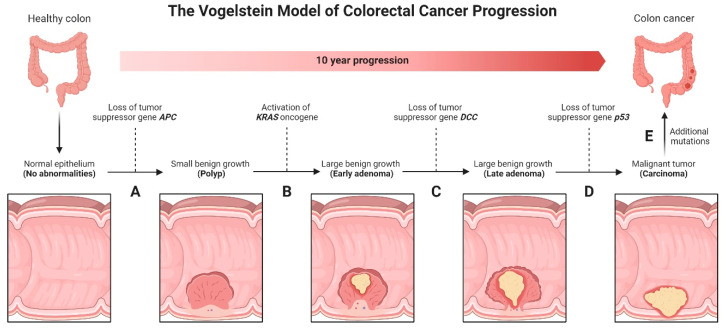
**The Vogelstein model of colorectal cancer progression.** This model was proposed by Bert Vogelstein and his colleagues at Johns Hopkins University in the early 1990s, based on their groundbreaking research on the genetic alterations involved in colorectal cancer (CRC). The initial stage shows a normal epithelium, representing a healthy colon lining with no visible abnormalities. (**A**) The first genetic event leads to the formation of a small benign growth polyp. This is a consequence of the inactivation of the *APC* gene, a critical tumor suppressor gene. (**B**) The next stage is associated with the progression from a small polyp to a large benign growth (early adenoma). The activation of the *KRAS* oncogene, a key driver of cellular proliferation, contributes to the growth and expansion of the adenomatous polyp. (**C**) This is followed by the loss of tumor suppressor gene *DCC*, which contributes to the development of a late adenoma or large benign growth. (**D**) The next step is the transition from a late adenoma to an invasive malignant tumor or carcinoma by the loss of tumor suppressor gene *TP53*. (**E**) The final stage represents a full-blown CRC, depicting an advanced, invasive malignant tumor mass. This stage may involve additional genetic alterations beyond the core events highlighted in the Vogelstein model. Figure created using BioRender.

The conventional adenoma–carcinoma–metastasis phenotypic transitions are associated with the accumulation of specific genetic alterations [51]. These events are often referred to as the “*APC*-*KRAS*-*TP53*” pathway or the Vogelstein model [101,102]. The Vogelstein model is named after Dr. Bert Vogelstein, a prominent cancer researcher who proposed this model to explain the stepwise progression of CRC [101,102]. According to this model (Figure 2), the development of CRC involves the sequential acquisition of genetic alterations in three key genes: *APC* (adenomatous polyposis coli), *KRAS*, and *TP53* [101,102,103]. The process is initiated by the inactivation of the *APC* gene [101,102,103], which normally functions as a tumor suppressor to regulate cell growth and division [104]. Mutation or loss of the *APC* gene function consequentially leads to uncontrolled cell growth and the formation of benign adenomatous polyps [105]. The next genetic alteration commonly observed in CRC is mutation in the *KRAS* gene [101,102,103], which is a potent oncogene regulating signaling pathways supporting cell proliferation [106]. *KRAS* mutation results in continuous activation of pathways promoting cell growth and survival, leading to the transition from adenoma to carcinoma [107]. The final genetic alteration in the Vogelstein model is the inactivation of the *TP53* gene, which is another tumor suppressor gene commonly referred to as the “guardian of the genome” [108]. *TP53* plays a critical role in DNA repair, cell cycle regulation, and apoptosis [109]. Mutation or loss of *TP53* function allow cells with accumulated genetic abnormalities to survive and proliferate, thus facilitating the development of invasive carcinoma and potential metastasis [110]. While the Vogelstein model specifically focuses on CRC, similar genetic alterations in the *APC*, *KRAS*, and *TP53* genes are observed in other cancer types, thus highlighting the broader relevance of these genes in cancer progression [111,112]. It is worth noting that the Vogelstein model represents a simplified framework for understanding the genetic events involved in cancer progression, and that additional genetic alterations and complex interactions among various genes and pathways can support CRC pathogenesis [112]. Some examples of these alternative processes will be discussed below.

### 2.2. Alternative Pathways of CRC Development: The Serrated Neoplasia Route

**Figure 3:**
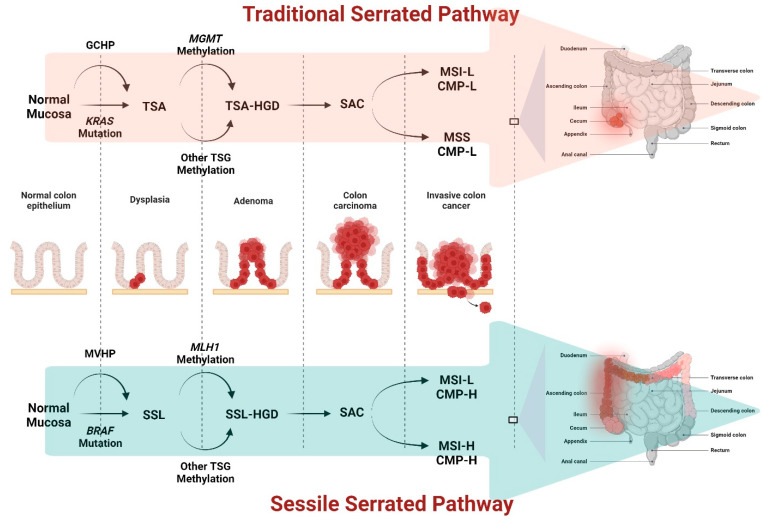
**The serrated neoplasia routes.** (A) The traditional serrated pathway begins with a *KRAS* mutation in normal colonic mucosa, leading to the formation of a traditional serrated adenoma (TSA). Further MGMT methylation and other TSG (tumor suppressor gene) methylation events promote the progression to TSA-HGD (traditional serrated adenoma–high grade dysplasia). Subsequent accumulation of genetic alterations results in the development of serrated adenocarcinoma (SAC), which can exhibit either an MSI-L/CMP-L (microsatellite instability–low/CpG island methylator phenotype–low) or MSS/CMP-L (microsatellite stable/CpG island methylator phenotype–low) molecular profile, indicative of invasive colorectal cancer (CRC). (B) The sessile serrated pathway initiates with a BRAF mutation in normal colonic mucosa, leading to the formation of sessile serrated lesions (SSL). Further *MLH1* methylation and other TSG methylation events promote the progression to SSL-HGD (sessile serrated lesion–high grade dysplasia). Subsequent accumulation of genetic alterations results in the development of serrated adenocarcinoma (SAC), which can exhibit either an MSI-L/CMP-H (microsatellite instability–low/CpG island methylator phenotype–high) or MSI-H/CMP-H (microsatellite instability–high/CpG island methylator phenotype–high) molecular profile, indicative of invasive CRC. Figure created using BioRender.

Although the conventional adenoma–carcinoma sequence is widely accepted pathway [113], not all CRCs develop through this sequence. To encompass CRC cases that arise from different mechanisms, additional models have been proposed such as the serrated pathway lesions [113] or inherited genetic mutations [114]. The serrated neoplasia pathway (Figure 3) is an alternative multistep process of CRC development that is distinct from the conventional adenoma–carcinoma sequence [115]. Approximately 15% to 20% of CRCs are thought to evolve through this pathway [116], which encompasses two different progression presentations: the sessile serrated pathway and the traditional serrated pathway [117]. The sessile serrated pathway is characterized by the development of sessile serrated adenomas (SSAs) [118] or sessile serrated lesions (SSLs) [119]. SSAs are typically flat or sessile polyps with serrated or sawtooth-like features on the surface [120]. They are predominantly found in the right colon [121] and are associated with certain molecular characteristics, such as mutations in the *BRAF* gene combined with DNA methylation abnormalities [122]. Over time, some SSAs can progress to dysplastic serrated lesions (DSLs) and eventually develop into CRC [123]. The traditional serrated pathway involves the progression from traditional serrated adenomas (TSAs) to CRC [124]. TSAs have a distinct histological appearance, characterized by tall columnar cells with abundant eosinophilic cytoplasm, serrated glands, and a characteristic “sawtooth” appearance [124,125]. They are often found in the proximal colon and are associated with mutations in the *BRAF* gene, as well as abnormalities in DNA methylation [126,127]. Another molecular feature associated with serrated neoplasms is increased CIMP [128]. CIMP refers to the hypermethylation of CpG islands in the promoter regions of specific genes [129]. As a result, gene expression is silenced and therefore contributes to the inactivation of tumor suppressor genes [129]. Serrated neoplasms, particularly sessile serrated adenomas/polyps (SSA/Ps), often exhibit a high degree of DNA methylation and subsequent CIMP, which may contribute favorably to their pathogenesis [128]. The presence of activating mutations in *BRAF* and *KRAS*, along with increased CIMP, helps distinguish serrated neoplasms from other colorectal polyps and contribute to CRC progression [130]. Both the sessile serrated pathway and the traditional serrated pathway are recognized as important paths to colorectal carcinogenesis [131]. Understanding these pathways is crucial for accurate diagnosis, management, and surveillance of patients at risk for CRC, particularly those with a family history of serrated lesions or Lynch syndrome, a hereditary condition associated with an increased risk of CRC [132].

### 2.3. Colitis-Associated Cancer: Inflammation-Driven CRC in Inflammatory Bowel Disease

**Figure 4:**
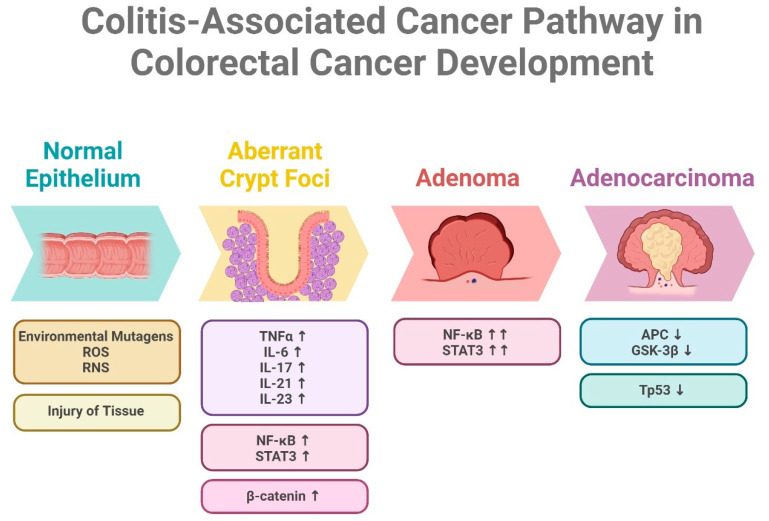
**Model of the colitis-associated cancer pathway in inflammatory bowel disease.** The figure depicts the multistage process by which chronic intestinal inflammation can lead to colorectal carcinoma development in the context of inflammatory bowel disease. The pathway is initiated by recurrent episodes of mucosal injury and inflammation in conditions such as ulcerative colitis (UC) or Crohn’s disease (CD). Prolonged inflammatory cell infiltration and cytokine/growth factor release results in accumulation of DNA damage and mutations in genes such as tumor suppressors (e.g., *TP53*) and oncogenes involved in Wnt/β-catenin signaling. Epigenetic alterations including DNA methylation changes also occur. This contributes to dysregulated epithelial proliferation and dysplasia. Immune system modulation favors an immunosuppressive microenvironment conducive to tumor growth. Through additional genetic and epigenetic changes, low- and high-grade dysplasia may develop, eventually progressing to adenocarcinoma, squamous cell carcinoma, or small cell carcinoma subtypes—the colitis-associated cancers (CAC). Figure created using BioRender.

Colitis-associated cancer (CAC) is another specific form of CRC that is closely associated with chronic inflammation of the colon, particularly in patients with inflammatory bowel disease (IBD) [133]. Patients with long-standing or severe ulcerative colitis (UC) also have an increased risk of developing CAC [134]. The chronic inflammation that occurs in the colon due to IBD can lead to genetic and molecular changes in the cells lining the colon, increasing the likelihood of cancer development [135]. The risk of developing CAC is directly proportional to the duration and extent of inflammation [136]. Patients with extensive and long-standing UC, especially those affecting the entire colon, are at the highest risk [137]. However, it is important to note that the majority of patients with IBD do not develop CAC, while it accounts for approximately 2% of all CRC cases [138]. The specific mechanisms underlying the transition from IBD to CAC are complex and multifactorial (Figure 4), involving various molecular pathways and interactions between genetic and environmental factors [139]. CAC exhibits a unique pattern of genetic alterations compared to sporadic or familial CRC [140]. Inflammation-associated molecular pathways, such as NF-κB (nuclear factor kappa-light-chain-enhancer of activated B cells), play a prominent role in CAC development [141]. Additionally, mutations in *TP53*, *APC*, and *KRAS* genes are also involved in inflammation pathways observed in CAC [142,143]. Moreover, alterations in DNA mismatch repair genes, such as *MLH1* and *MSH2* have been associated with CAC although less common in comparison to sporadic CRC [144,145]. The management of CAC requires a multidisciplinary approach that considers both the underlying IBD and the associated cancer [146]. Treatment options for CAC may include surgical resection, chemotherapy, radiation therapy, and targeted therapies. However, the presence of IBD poses unique challenges in the treatment of CAC [134]. For example, in some cases, colectomy (removal of the colon) may be necessary to eliminate the source of inflammation and reduce the risk of cancer recurrence [147].

## 3. Molecular Classification of CRC: The Consensus Molecular Subtypes

To better characterize the heterogeneity of CRC, researchers have classified tumors into four consensus molecular subtypes (CMS) [148]. These subtypes, designated CMS1 to CMS4, represent distinct molecular and clinical features of CRC [148]. As shown in Table 2, each subtype is associated with specific genetic alterations, gene expression profile, and clinical outcomes [148,149]. CMS1 CRC tumors are characterized by high levels of microsatellite instability (MSI) and immune cell infiltration [150,151]. These tumors often display a strong immune response and are associated with favorable clinical outcomes [148]. CMS2 tumors exhibit classical molecular features of CRC, including activation of the Wnt signaling pathway and epithelial differentiation [152]. These tumors are the most common subtype and are associated with intermediate clinical outcomes [149]. CMS3 tumors are characterized by metabolic dysregulation and are often associated with obesity [153]. They display altered metabolic gene expression signatures and have distinct clinical features and outcomes [148,153,154]. CMS4 tumors exhibit prominent stromal infiltration and activation of pathways involved in epithelial–mesenchymal transition (EMT) typically associated with cancer malignancy [155,156]. These tumors have poor clinical outcomes and are often resistant to standard treatments [156]. The classification of CRC into these consensus molecular subtypes has provided a framework for understanding the heterogeneity of the disease and has implications for prognosis and treatment [157].

### Experimental Models for Interrogating Intratumoral Heterogeneity in CRC Subtypes

**Figure 5:**
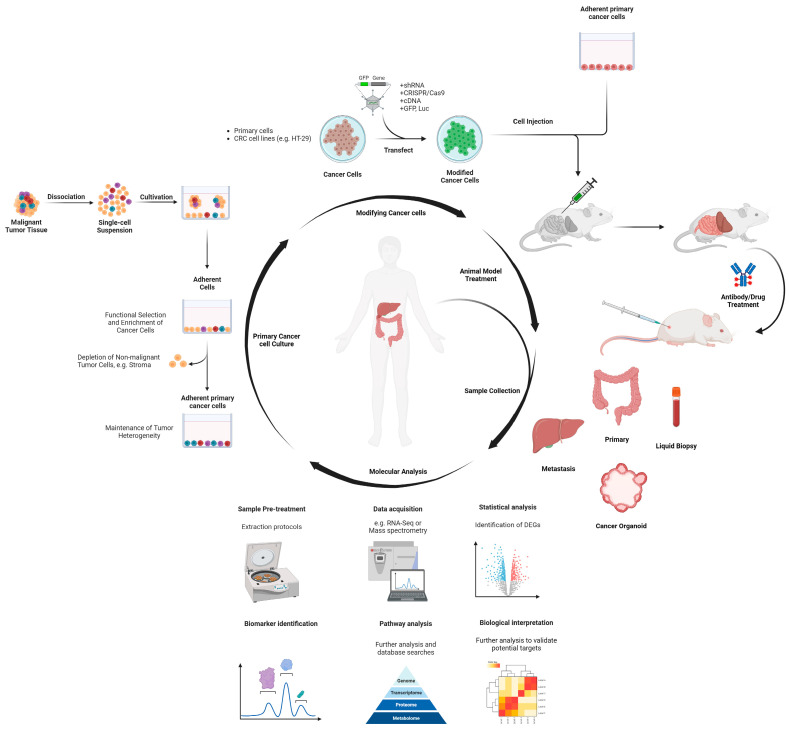
**Leveraging patient-derived models to investigate intratumoral heterogeneity in colorectal cancer subtypes.** Primary tumor tissues dissociated from colorectal cancer (CRC) patients serve as the starting point for generating diverse experimental models. Single-cell suspensions allow enrichment of adherent cancer cells while depleting non-malignant stromal components, maintaining intratumoral heterogeneity. The enriched cancer cells can be directly cultured as patient-derived cell lines or used to derive three-dimensional organoid cultures that recapitulate aspects of the tumor microenvironment. Additionally, these patient-derived cells can be modified through transfection with *siRNA*, *shRNA*, *cDNA*, or *CRISPR/Cas9* gene editing before utilization in downstream applications. One key application is injection into immunocompromised mouse models to establish patient-derived xenograft (PDX) tumors. Sample collection from these in vivo models provides primary tumors, metastases, and liquid biopsy samples for comprehensive molecular analyses. Established CRC cell lines like HT-29 offer an alternative source for generating xenograft models and modified sublines. Multi-omics data acquisition through techniques like RNA sequencing, mass spectrometry proteomics, and identification of differentially expressed genes enables biomarker discovery, pathway analysis, and biological interpretation of distinct CRC subtypes. Potential therapeutic targets derived from these analyses are validated through antibody/drug treatment studies in the PDX and cell line xenograft models. This multifaceted strategy integrating patient-derived models, genetic modifications, organoids, xenografts, and multi-omics profiling facilitates investigations into the complexity of intratumoral heterogeneity underlying CRC. Figure created using BioRender.

The study of CRC subtypes encompasses various approaches to capture their versatile features (Figure 5). These approaches involve the utilization of different model systems, including chemically induced and genetically engineered mouse models [158], patient-derived cell lines [159], organoids [159], and xenografts [160]. Each of these study models offers distinct advantages and allows researchers to explore specific aspects attributable to CRC subtypes [160,161,162]. Chemically induced mouse models involve the manipulation of mice through chemical carcinogenic agents such as azoxymethane (AOM) [163,164,165,166] or dextran sulfate sodium (DSS) [166,167] to induce CRC [158]. Similarly, genetically engineered mouse models (GEMMs) are created by introducing specific genetic alterations relevant to CRC development [168]. These models are more physiologically relevant to CRC and enable researchers to study the fundamental mechanisms driving cancer initiation, progression, and response to treatment [158].

Patient-derived cancer cell lines also provide valuable tools for studying CRC subtypes in vitro [169]. These cell lines are often established from primary tumor tissues or metastatic lesions and can be maintained and propagated in laboratory settings [170]. Researchers can use these cell lines to investigate various aspects of CRC, including its molecular characteristics, drug sensitivity, and mechanisms of resistance [169,170]. Although they do not represent a multi physiological setting (i.e., animal model), they serve as a renewable resource of CRC cells for toxicity assays and elucidating the underlying biology of CRC subtypes at the cellular level [170,171]. Organoids are three-dimensional cultures of epithelial cells that can recapitulate the characteristics and architecture of the original tissue [172]. In the case of CRC, organoids can be generated from tumor tissue or normal colon epithelial cells [173]. These organoid models allow researchers to study the behavior of CRC subtypes in a more physiologically relevant environment [174]. They can also be used to investigate tumor growth, invasion, drug response, and personalized medicine approaches [175].

Finaly, xenograft models involve the transplantation of human CRC cells or tissues into immunodeficient (humanized) mice [176,177]. These models allow the study of tumor behavior and response to therapy in an in vivo setting [178]. Patient-derived xenografts (PDX) are created by implanting patient tumor tissues (biopsies) directly into mice [179]. PDX models retain the heterogeneity and molecular characteristics of the original tumor, making them valuable tools for studying CRC subtypes and testing therapeutic interventions in a physiological environment [180]. By employing a combination of these diverse model systems, researchers can gain a comprehensive understanding of CRC subtypes, unravel their molecular features, and explore potential treatment strategies [181]. Each model system provides unique advantages, and their integration allows for a more robust characterization of CRC subtypes and the development of personalized approaches for patients [148,181,182,183,184].

## 4. Hallmarks of Colorectal Cancer

### 4.1. Genome Instability and Mutations in CRC Driver Genes

**Figure 6:**
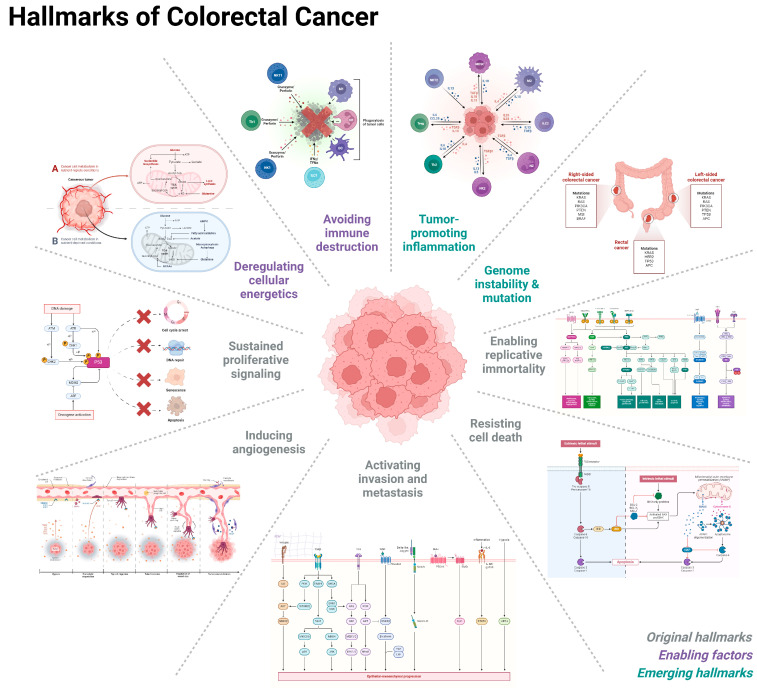
**Mechanisms underlying the hallmarks of colorectal cancer progression.** Colorectal cancer (CRC) is a complex and multifaceted disease characterized by the acquisition of various hallmark capabilities that enable tumor growth, progression, and metastasis. Sustained proliferative signaling in CRC tumors is driven by modifying mechanisms like cell cycle arrest, DNA repair, senescence, and apoptosis, while evasion of growth suppressors occurs through disruptions in tumor suppressors like *TP53* and *APC*. Resistance to cell death is facilitated by dysregulation of apoptotic machinery and BCL-2 family members, and replicative immortality is enabled by deregulation of pathways like WNT, RAS, and PI3K. Angiogenesis is induced by factors like VEGF and hypoxic conditions, while invasion and metastasis involve epithelial–mesenchymal transition, altered cell–cell adhesion, and extracellular matrix remodeling. Deregulation of cellular energetics, such as the Warburg effect, provides a growth advantage, and immune evasion is mediated by mechanisms like PD-L1 upregulation. A tumor-promoting inflammatory microenvironment is created by cytokines, chemokines, and immune cell infiltration, while genomic instability and tumor progression are driven by the accumulation of mutations in genes like *APC*, *KRAS*, and *TP53*. The image further depicts emerging hallmarks specific to CRC, including the distinct molecular features of left-sided and right-sided tumors, unique characteristics of rectal cancer, and the involvement of signaling pathways like Wnt, Notch, Hedgehog, and TGF-β in disease progression. Figure created using BioRender.

Similar to other cancers, CRC is a complex disease characterized by the accumulation of genomic alterations [185]. In fact, genome instability, which refers to an increased propensity for genetic alterations within cells (Figure 6), plays a significant role in CRC development and progression [186,187]. There are two primary forms of genome instability in CRC: CIN [188] and MSI [189]. CIN is described by an abnormal number or structure of chromosomes within cells [190] and is observed in approximately 70–80% of CRC cases [188]. The key features of CIN include: (1) aneuploidy, which is an abnormal number of chromosomes in cells and results in gains or losses of specific chromosomal regions [191]; (2) structural alterations in chromosomes, such as deletions, duplications, inversions, or translocations that can affect the expression of key genes involved in cancer development and progression [192]; (3) complex genomic profiles with numerous chromosomal alterations that contribute to tumor heterogeneity and can impact the response to treatment [193]; and, (4) a wider spectrum of mutations affecting various cancer-related genes, including tumor suppressor genes and oncogenes [194,195].

MSI is another form of CRC genome instability, which refers to a distinct accumulation of small insertions or deletions in repetitive DNA sequences known as microsatellites [189,196]. MSI is observed in approximately 15–20% of CRC cases [189]. It arises due to impaired DNA mismatch repair (MMR), a system responsible for correcting errors during DNA replication [197]. MMR deficiency therefore leads to the accumulation of genetic mutation in microsatellite regions [198]. MSI tumors can also exhibit variable lengths in microsatellite regions due to the insertion or deletion of repeat units [199]. In fact, this unique feature is used to assess MSI status of a tumor in clinical practice [200]. MSI tumors often possess a higher degree of immune cell infiltration, particularly tumor-infiltrating lymphocytes (TILs), which can influence the tumor microenvironment and response to immunotherapy [201,202].

MSI tumors also display specific mutation profiles in genes involved in DNA repair, such as MMR genes (e.g., *MLH1*, *MSH2*, *MSH6*, *PMS2*) [203]. These errors can cause mutations in important cancer-related genes and contribute to tumor development [204]. Other mutations may also occur but are less common compared to CIN tumors [189]. This is because the majority of mutations in MSI tumors are localized to the microsatellite regions, while the rest of the genome remains relatively stable [205]. In contrast, CIN tumors exhibit higher rates of genomic instability affecting various regions of the genome, leading to a higher overall mutation burden [189]. MSI tumors often respond favorably to immune checkpoint inhibitors, which can harness the enhanced immune response observed in these tumors [206,207]. MSI is more common in hereditary forms of CRC, such as Lynch syndrome, but can also occur sporadically [208].

Genetic mutations occur when there are changes in the consensus DNA sequence of specific genes [209]. Several genes have been identified as being frequently mutated in CRC (Figure 6) [210]. The most common driver mutations in CRC affect the Wnt signaling pathway in addition to the *APC*, *KRAS*, *BRAF*, and *TP53* genes [210]. Mutations in these genes disrupt normal cellular processes, leading to uncontrolled cell growth, increased survival, and the acquisition of invasive properties [211,212]. For example, mutations in the Wnt signaling pathway are considered early events in CRC development [213]. On the other hand, the *APC* gene, which normally regulates the pathway, is frequently mutated in both hereditary and sporadic CRC [214]. Inactivation of the *APC* gene leads to inducted Wnt signaling resulting in uncontrolled cell growth and division [215]. Specifically, *APC* mutations correlated with the formation of polyps, which can progress to adenomas and eventually carcinoma [216]. *KRAS* and *BRAF* alterations are also commonly found in CRC, where *KRAS* mutation leads to the constitutive activation of the RAS signaling pathway, cell survival, and proliferation [216,217], whereas *BRAF* alterations, particularly the V600E mutation, activate the MAPK signaling pathway which contribute to tumor growth [218]. *KRAS* and *BRAF* mutations are both associated with a poor clinical prognosis in CRC [219,220]. The most well-described and prominent *TP53* tumor suppressor gene is also involved in CRC genomic stability [221]. In fact, *TP53* mutations are found in a significant proportion of CRC cases associated with advanced tumor staging, resistance to therapy, and poor prognosis [222,223]. In addition to the key driver mutations mentioned above, CRC can also harbor various other genetic alterations in genes regulating DNA repair (i.e., *POLE*, *POLD1*) [224], chromatin remodeling (i.e., *ARID1A*) [225], and cell cycle regulation (i.e., *PIK3CA*, *SMAD4*) [226]. Together, these genetic profiles not only contribute to the heterogeneity and complexity of CRC, but also define the molecular nomenclature supporting cancer stratification and therapeutic approaches [224,225,226,227]. However, genome instability and mutations are not the sole factors contributing to CRC pathogenesis. Environmental factors, lifestyle choices, and other genetic and epigenetic alterations also play significant roles in the initiation and progression of CRC [228,229,230].

#### Telomere Dysfunction/Reactivation

Telomeres are repetitive DNA sequences located at chromosome extremities, which play a critical role in maintaining genomic stability and protecting the integrity of the genome [231]. With each cell division, telomeres gradually shorten with the aging process, and when they become critically short, it triggers cellular senescence or apoptosis to limit cell proliferation and prevent the propagation of damaged DNA [232]. Telomere dysfunction, including shortening or loss of telomeres, has been implicated in various age-related diseases and cancer malignancies [233]. The reactivation of telomerase in cancer serves to counteract telomere shortening and allows cancer cells to bypass typical cellular senescence and apoptosis checkpoints, thus enabling uncontrolled cell division and tumor growth [234]. Telomerase reactivation is considered one of the hallmarks of cancer, and its targeting has been intensively explored as a potential therapeutic strategy [235].

In the early stages of CRC, telomere-based crisis can occur due to critically short telomeres that trigger DNA damage responses [236]. This crisis leads to extensive chromosomal rearrangements and genomic instability, a phenomenon known as CIN [237]. During telomere crisis, cells undergo multiple cycles of DNA damage, repair, and breakage–fusion–bridge (BFB) cycles [238]. BFB cycles are the result of broken DNA ends fusing with other chromosomes during DNA repair, leading to further genetic abnormalities and chromosomal rearrangements [238]. These cycles can result in the amplification of oncogenes or the inactivation of tumor suppressor genes, all of which promote cancer development and progression [239,240]. In CRC, telomere-based crisis and subsequent CIN can facilitate the acquisition of additional genetic alterations necessary for tumor growth and survival [241].

Several approaches have been investigated to target telomerase in CRC. One of these involves the use of small molecule inhibitors including BIBR1532 [242,243], RHPS4 (BRACO-19, Imidazole quinoline derivative) [244,245], and MST-312 (Silybin derivative) [246], that selectively block telomerase activity to prevent telomere elongation and dysfunction in CRC cells [247]. Mechanistically, BIBR1532 is a non-nucleosidic telomerase inhibitor that acts by disrupting the interaction between the telomerase enzyme and its telomeric DNA substrate [242,243]. It also inhibits telomerase catalytic activity thereby impairing telomere maintenance [243]. While BIBR1532 has been primarily studied in the context of hematological malignancies [248,249,250,251], its potential efficacy in solid tumors including CRC [242], breast cancer [252], non-small cell lung cancer (NSCLC) [253], and oral squamous cell carcinoma (OSCC) [254], has also been recently explored. On the other hand, RHPS4 is a small synthetic molecule that targets the G-quadruplex structures formed at the telomeric DNA [244] where it binds and prevents telomerase from elongating the telomeres [245]. RHPS4 has exhibited promising anti-telomerase activity in various cancer models, including CRC [255,256,257]. Finaly, MST-312 is a compound found in milk thistle and has been reported to inhibit telomerase activity by interfering with the telomerase enzyme complex assembly [246]. MST-312 has demonstrated telomere shortening effects in CRC cells and may soon be applied as an anti-cancer agent in preclinical studies [246].

Other targeting strategies include the inhibition of telomerase RNA components (i.e., TER or TERC), which are essential for telomerase expression and function [258]. Inhibiting TERC is an area of active research and holds potential for therapeutic intervention as it is shown to effectively disrupt telomerase activity leading to telomere shortening in cancer cells [259,260]. To date, several approaches have successfully targeted TERC in CRC through the use of antisense oligonucleotides (ASOs) [261], RNA interference (RNAi) [262,263], and small-molecule scaffolds (i.e., GRN163L/imetelstat and CX-5461) [263,264]. ASOs are short synthetic strands of nucleotides that are complementary to specific RNA sequences [265]. The design of TERC-specific ASOs therefore inhibit telomerase activity [266] and has shown promise in preclinical studies in various cancers, including CRC [267]. RNAi has also been used to inhibit telomerase activity in CRC [262]. This is performed by introducing small interfering RNA (*siRNA*) molecules that target TERC, which then initiates a natural process that regulates gene expression by silencing specific RNA molecules (i.e., TERC) [268]. Researchers are still actively exploring the potential of RNAi-based therapies for targeting telomerase in cancer cells [269]. Another approach involves the development of small-molecule scaffolds that specifically bind to TERC and inhibit telomerase function [264,270]. These molecules, known as telomerase inhibitors, disrupt the proper assembly and stability of telomerase, leading to its inactivation [271]. For example, imetelstat is a synthetic oligonucleotide that directly targets and binds to an RNA component of telomerase (called hTERC) resulting in telomerase inhibition and telomere shortening in CRC cells [270]. CX-5461 is another a selective inhibitor of RNA polymerase I transcription, which indirectly affects telomerase activity by reducing the synthesis of telomerase RNA components [272,273]. CX-5461 has shown promising inhibitory effects on telomerase activity in CRC and has been studied in preclinical models [272,273]. Furthermore, alternative strategies aimed to exploit the reliance of telomerase-positive cancer cells on telomere maintenance have been developed [274]. For instance, telomerase-specific oncolytic viruses have been created to selectively replicate and eliminate telomerase-positive cancer cells by exploiting the dependence of these cells on functional telomerase for telomere elongation [275]. Although the targeted inhibition of telomerase activity has demonstrated promise as a potential therapeutic strategy for CRC, further research is needed to better understand its efficacy, safety, and potential side effects [276]. Clinical trials are currently ongoing to determine the optimal approach and to evaluate the long-term effects of targeting telomerase in CRC patients [277].

### 4.2. Enabling Replicative Immortality

Sustaining proliferative signaling is a hallmark of cancer, including CRC [278]. In normal cells, the process of cell proliferation is tightly regulated, and cells divide and grow in a controlled manner [279]. However, in CRC, certain genetic alterations disrupt the normal regulation of cell growth (Figure 6), leading to the sustained proliferative signaling characteristic of cancer [280]. Two key molecular pathways involved in sustaining proliferative signaling in CRC are the EGFR and Wnt signaling pathways [281,282].

#### 4.2.1. EGFR Signaling Pathway

Activation of EGFR triggers downstream signaling cascades, including the RAS/RAF/MEK/ERK and PI3K/AKT pathways (Figure 6) [283]. These pathways are essential for transmitting signals from the cell surface to the nucleus, ultimately leading to various cellular responses such as proliferation, survival, and differentiation [284]. When EGFR is activated by its ligands, such as epidermal growth factor (EGF), it undergoes a conformational change that allows it to dimerize and autophosphorylates specific tyrosine residues located in its intracellular domain, creating docking sites for downstream signaling molecules such as RAS, RAF, MEK, and ERK pathway components [285,286,287]. Upon EGFR activation, the small GTPase protein RAS is recruited to the plasma membrane, where it is activated by guanine nucleotide exchange factors (GEFs) [288]. Activated RAS then recruits and activates RAF kinase, which subsequently activates MEK (MAPK/ERK kinase) [289]. MEK phosphorylates and activates ERK (extracellular signal-regulated kinase), which then translocates to the nucleus and phosphorylates various transcription factors, leading to gene expression changes involved in cell proliferation, survival, and differentiation [290]. Another important pathway activated by EGFR is the PI3K/AKT cascade [291]. EGFR activation leads to the recruitment of phosphatidylinositol 3-kinase (PI3K) to the receptor complex [292]. PI3K phosphorylates phosphatidylinositol 4,5-bisphosphate (PIP2) to generate phosphatidylinositol 3,4,5-trisphosphate (PIP3), which serves as a docking site for AKT for its activation by phosphoinositide-dependent protein kinase 1 (PDK1) [293,294]. Given that activated AKT regulates multiple downstream effectors involved in cell survival, metabolism, and protein synthesis, this cascade is also a prevalent cancer signaling gateway [295].

Dysregulation of RAS/RAF/MEK/ERK and PI3K/AKT signaling cascades can contribute to the development and progression of several diseases, including cancer [296]. In CRC, the most common genetic alterations affecting this signaling pathway are found in the *RAS* gene, especially *KRAS* and *NRAS* (Figure 6) [297]. It involves a single nucleotide substitution, leading to a glycine amino acid replacement by either an aspartic acid at position 12 (G12D) or, by a valine at position 13 (G13D) [298]. Other less common mutations occur at positions 61 (Q61H) and 146 (A146T) [298,299]. *KRAS* G12D and G13D mutations are found in approximately 30–45% of CRC cases, leading to constitutive activation of RAS and aberrant activation of downstream signaling [298,299,300]. Colorectal cancer patients with *KRAS* mutations are often associated with poor prognosis, less responsive to certain targeted therapies, and have a higher risk of disease recurrence when compared to patients with wild-type *KRAS* [301,302,303,304,305,306]. *KRAS* mutations are also valuable predictive markers for the efficacy of anti-EGFR therapies, such as cetuximab [302,303,304] and panitumumab [305,306]. Patients with *KRAS* mutations in codons 12 or 13 are typically resistant to these drugs [302,303,304], and testing for *KRAS* mutation status is now standard practice upon the consideration of anti-EGFR therapy [307,308,309]. Nonetheless, patients with *KRAS* mutations may undergo other targeted therapies, such as MEK inhibitors, which are currently being investigated as effective treatment options for CRC patients with *KRAS* mutation signatures [310].

Furthermore, dysregulation of other components of this pathway, such as RAF, MEK, or ERK, can also occur in CRC [287]. Mutations in the *BRAF* gene, which is part of the RAF protein family, are found in a subset of CRCs and affect the behavior and treatment of the disease [311]. However, BRAF mutations are relatively uncommon in CRC, accounting for approximately 5–15% of cases [312,313]. The most prevalent BRAF mutation in colorectal cancer is the V600E mutation, where valine (V) is substituted with glutamic acid (E) at position 600 [314]. This mutation causes a hyperactive BRAF kinase, leading to increased downstream signaling [315]. BRAF mutations in CRC are associated with poor prognosis as patients tend to have more aggressive disease, advanced stages at diagnosis, higher likelihood of lymph node involvement, and lower overall survival rates compared to patients with BRAF wild-type tumors [316]. BRAF mutation is also frequently observed in specific tumor subtypes, such as those with MSI-high MSI-H or CIMP features [317]. These subtypes often exhibit distinct clinicopathological characteristics and have implications for treatment strategies [150,151,318]. For instance, BRAF mutant tumors might be less sensitive to anti-EGFR treatment, and some BRAF mutations may not be sensitive to any particular targeted therapies [150]. Therefore, other therapeutic options for BRAF V600E have been developed, such as vemurafenib [319] and encorafenib [320], which are often used in combination with other agents like MEK inhibitors (i.e., cobimetinib or binimetinib). These therapeutic interventions have shown promising results in improving outcomes for CRC patients with BRAF mutation [321]. Nevertheless, ongoing clinical trials are exploring novel treatment approaches for BRAF-mutated colorectal cancer, including combinations of targeted therapies, immunotherapies, and chemotherapy [311].

Mutations in genes encoding components of the PI3K/AKT pathway can also result in its dysregulation, leading to uncontrolled cell growth and tumor formation [311]. The most common mutations occur in the *PIK3CA* gene, which encodes the catalytic subunit of PI3K (p110α), and in the *PTEN* (phosphatase and tensin homolog) gene, a negative regulator of the pathway [322,323]. *PIK3CA* mutations are detected in approximately 15–20% of CRC cases and represent the most frequent genetic alterations in CRC, particularly in tumors arising from the left side of the colon [324,325]. PIK3CA mutations are often mutually exclusive with other well-known driver mutations in CRC, such as *KRAS* and BRAF mutations [326]. However, they may co-exist with other gene mutations involved in the PI3K pathway, such as *PTEN* and AKT [327]. Due to the complexity in these signaling components, studies have shown conflicting results regarding the prognostic significance of PIK3CA mutations in CRC. For example, some studies suggest that PIK3CA mutation may be associated with a favorable prognosis [328,329,330,331], while others indicate no significant impact on overall survival or disease-free survival [330,332]. Nonetheless, PIK3CA mutations have gained attention as a potential predictive biomarker for targeted therapies [333,334]. Preclinical studies have demonstrated that CRC cells with PIK3CA mutations may be sensitive to PI3K inhibitors, such as alpelisib [335]. Several clinical trials are ongoing to evaluate the efficacy of PI3K inhibitors in CRC patients with PIK3CA mutations to determine whether targeting the PI3K pathway can improve treatment outcomes in specific patient populations [336,337]. However, similar to other targeted therapies, resistance to PI3K inhibitors can develop over time as alternative downstream signaling components, activation of compensatory pathways, or the emergence of additional mutations may contribute to resistance [338,339]. Given the complex nature of CRC and the heterogeneity of PI3K pathway alterations, combination therapies involving PI3K inhibitors with other targeted agents or chemotherapy drugs are being explored to improve treatment response and overcome resistance [340,341].

AKT, also known as protein kinase B (PKB), is another key signaling protein involved in various cellular processes such as cell growth, survival, and metabolism [342]. In CRC, the most frequent AKT mutations involve the *AKT1* gene [343]. Although relatively rare (2–6%) in comparison to *APC*, *KRAS*, *TP53*, and *PIK3CA* genes [344,345], *AKT1* mutations mainly contribute to the development and progression of cancer [344]. The most common *AKT1* gene mutation observed in solid tumors is a missense mutation known as E17K (G49A) [346]. This mutation occurs in the PH (pleckstrin homology) domain of the *AKT1* protein where a glutamic acid (E) is replaced by a lysine (K) at position 17, leading to its constitutive activation [345]. Studies have shown that the AKT^E17K^ mutation is associated with more aggressive tumor behavior, increased resistance to chemotherapy, and poorer patient outcome compared to CRC cases without this mutation [347]. In fact, AKT^E17K^ mutation has been considered as a potential therapeutic target in cancers for several targeted therapies and inhibitors of the AKT signaling pathway like ARQ751 and ARQ092 [348,349,350], capivasertib or AZD5363 [351,352,353], and BAY1125976 [354], which are currently being developed and tested in clinical trials to specifically target and inhibit the downstream effects of this mutation. Molecular testing, such as DNA sequencing, can also be used to detect the presence of AKT^E17K^ mutation in cancer patients [355]. Therefore, identifying patients who may benefit from targeted therapies or personalized treatment approaches [350]. Nevertheless, the frequency of this mutation may vary depending on the population studied and the methodology used for mutation detection [356].

Activated AKT exerts its downstream effects by phosphorylating and regulating numerous targets within the cell (Figure 6) [357]. One of its major targets is mTOR, a central regulator of cell growth and metabolism, which exists in two distinct complexes: mTORC1 and mTORC2 [358]. AKT-mediated phosphorylation of mTORC1 enables its activation, which regulates protein synthesis, cell growth, and metabolism in response to nutrient availability and growth factors [359]. In CRC, genetic alterations in *PIK3CA* and *PTEN* genes lead to sustained activation of the PI3K/AKT/mTOR pathway, promoting cell survival, proliferation, angiogenesis, and resistance to apoptosis [360]. The dysregulation of the mTOR pathway in CRC has led to the exploration of mTOR inhibitors as potential therapeutic agents [361,362,363]. Drugs like everolimus [364,365] and temsirolimus [366,367], have shown some efficacy in specific subsets of CRC patients with mTOR pathway alterations [368]. Despite the initial promise of mTOR inhibitors, resistance to these drugs can develop [369]. These mechanisms mostly include feedback activation of upstream signaling components, alternative pathway activation, and mutations in downstream effectors [370]. Due to the complexity of drug resistance and the mTOR pathway, combination therapies targeting multiple nodes in the pathway or combining mTOR inhibitors with other agents are being investigated as potential strategies to improve treatment outcomes in CRC patients with mTOR pathway mutations [371].

Overall, the PI3K/AKT/mTOR signaling pathway integrates and responds to a wide range of intracellular and extracellular signals, allowing cells to coordinate their growth, metabolism, and survival in response to various physiological and environmental cues [372]. Interaction between the PI3K/AKT/mTOR pathway and other CRC signaling pathways, such as the Wnt/β-catenin pathway and the MAPK/ERK pathway, establish a complex network of signaling crosstalk that contributes to the overall pathogenesis of colorectal cancer [373].

#### 4.2.2. Wnt/β-Catenin Signaling

The Wnt/β-catenin signaling pathway plays a crucial role in promoting and maintaining cancer cell stemness (Figure 6) [374]. Stem cells are undifferentiated cells that have the ability to self-renew and give rise to different cell types in the body [375]. In the absence of Wnt signaling, a protein called β-catenin is phosphorylated by a complex of proteins, which leads to its degradation [376]. However, when Wnt ligands bind to their receptors on the cell surface, it activates a signaling cascade that inhibits β-catenin degradation by a destruction complex comprising *APC*, Axin, GSK3β (Glycogen synthase kinase 3 beta), and CK1 (Casein kinase 1) [377]. As a result, β-catenin accumulates in the cytoplasm and translocates into the nucleus, where it interacts with transcription factors of the T-cell factor/lymphoid enhancer factor (TCF/LEF) family [378]. This interaction leads to transactivation of target genes including *c-Myc*, *cyclin D1*, and matrix metalloproteinase 7 (*MMP7*) that regulate cell proliferation, survival, and differentiation [379]. In normal stem cells, controlled activation of the Wnt/β-catenin pathway is essential for maintaining self-renewal capacity and tissue homeostasis [380]. However, dysregulation of this cascade leads to aberrant stem cell behavior and contributes to the development of various cancer types, including CRC [381].

In cancer cells, Wnt/β-catenin pathway is often hyperactivated [381]. This occurs through various mechanisms, including ligand-dependent or ligand-independent Wnt signaling activation [382]. In the canonical pathway, secreted Wnt ligands bind to specific cell surface receptors, which include the very well-established Frizzled (FZD) receptor proteins and low-density lipoprotein receptor-related protein (LRP) 5/6 co-receptors [383,384]. Upon ligand binding, the canonical Wnt pathway leads to the stabilization and nuclear translocation of β-catenin [385]. On the other hand, ligand-independent Wnt activation includes alterations in upstream regulators like *APC* [386] or key components of the CTNNB1 pathway [387] and destruction complex components [388]. Altogether, these paths lead to sustained activation of Wnt/β-catenin signaling in CRC cells characterized by enhanced self-renewal capacity, resistance to therapy, and the ability to initiate tumor formation [389,390]. Sustained Wnt/β-catenin activation is also important in maintaining CRC stem cells (CSCs), which are thought to be responsible for tumor initiation, heterogeneity, and recurrence [389,390].

Mutations in several key components of the Wnt signaling pathway have been identified in CRC, including genes encoding ligands, receptors, and downstream effectors [391]. One of the most mutated genes in this pathway is the *APC* gene [391,392]. The *APC* protein normally acts as a negative regulator of Wnt signaling by promoting the degradation of β-catenin [391,392]. *APC* mutations are found in approximately 80% of sporadic CRC cases, making it the most frequently mutated gene in this pathway [392]. *APC* gene mutations therefore lead to the accumulation of β-catenin and constitutive activation of Wnt signaling, which can drive colorectal tumor formation [392]. Other CRC gene mutations associated to the Wnt pathway include *CTNNB1* (encoding β-catenin), *AXIN2*, *TCF7L2*, and *LRP5/6* [391]. The prevalence of these gene alterations vary among CRC cases [393] where *CTNNB1* mutations occur in about 10–15% of CRC cases, whereas *AXIN2*, *TCF7L2*, and *LRP5/6* are relatively rare mutations but have been reported in a small percentage of cases [393,394].

Given the prominent role of Wnt signaling in CRC, most components of this pathway represent potential therapeutic targets [395]. One approach has directly targeted the key downstream effector of the pathway, β-catenin. In this regard, small molecules like ICG-001 [396], PRI-724 [397], CGP049090 [398], C-82 [398], and BC2059 [399] have been shown to prevent β-catenin nuclear translocation, disrupting the transcriptional activity of the pathway and inhibiting tumor growth. Other strategies have targeted components of the destruction complex, such as GSK3β [400] or the tankyrase enzyme, which regulate β-catenin levels and activity [401]. For example, the novel tankyrase inhibitor OM-153 has proven to reduce Wnt/b-catenin signaling and tumor progression in preclinical CRC models [402] and could be used in clinical trials. In addition, novel antibodies are being developed to inhibit specific Wnt pathway components including the Porcupine enzyme, which is involved in the secretion and maturation of Wnt ligands [403]. These inhibitors block the production of both ligand-dependent and ligand-independent Wnt signaling, showing promise as potential therapeutic agents for CRC [404,405]. Nevertheless, challenges in the development of β-catenin inhibitors still remain as the identity of patients who are most likely to benefit from this therapeutic approach by overcoming potential resistance is still underway [406]. Addressing these issues requires a multidisciplinary approach involving collaboration between researchers, clinicians, and pharmaceutical companies [406]. Continued research and clinical trials will be essential to overcome these obstacles and realize the potential of β-catenin inhibitors as effective targeted therapies for cancer [406].

In summary, the EGFR and Wnt/β-catenin pathways are critical players in CRC [406]. Their dysregulation through genetic and epigenetic alterations contributes to uncontrolled cell proliferation, survival, and invasion, which are key characteristics of cancer cells [407]. Understanding these pathways and developing targeted therapies against them have shown promise in the treatment of CRC [374]. These targeted therapies have the potential to improve patient outcomes, reduce side effects, and overcome resistance to traditional chemotherapy [408].

#### 4.2.3. Overriding Restraints: Cancer’s Growth and DNA Damage Tolerance

One aspect of CRC development involves the evasion of growth suppressors, which normally regulate cell growth and prevent uncontrolled proliferation (Figure 6) [409,410]. Tumor suppressor genes such as *APC*, *TP53*, and *SMAD4* are commonly mutated in CRC [409,410]. Mutations in the *APC* gene are considered initiating events in most sporadic cases of CRC and commonly locate to the 8q, 13q, 18q, and 20q chromosomal regions in most colorectal adenocarcinomas [409,411]. Mutations in 18q often result the translation of a truncated *APC* gene product and non-functional protein [412]. Lost or impaired function of *APC* leads to the development of FAP, an inherited condition characterized by the formation of numerous polyps in the colon and rectum [413]. FAP also significantly increases the risk of developing CRC [414]. Disruption of *APC* function can also impact other cellular processes beyond the Wnt pathway [373]. For instance, *APC* is involved in cell migration, adhesion, and cytoskeletal organization by regulating the dynamics of the actin cytoskeleton, and its dysfunction can lead to abnormalities in these processes [415,416,417]. *APC* is also implicated in the maintenance of chromosomal stability and proper segregation of chromosomes during cell division [418]. *APC* mutation can consequently lead to chromosomal instability and increased risk of genomic aberrations [419].

While mutations in the *APC* gene are considered early events in CRC development [412], additional genetic alterations are usually required for advanced tumorigenesis and disease progression. Such mutations can include alterations in the genes involved in cell cycle regulation, such as *TP53* [420]. In healthy cells, *TP53* acts as a checkpoint regulator, preventing the proliferation of cells with damaged DNA [421]. When *TP53* is mutated, the ability to repair damaged DNA is compromised, leading to an accumulation of genetic alterations that promote cancer progression [422]. While *TP53* mutations can occur in different regions of the gene, certain hotspots are more commonly affected such as exons 5–8, which encode the DNA-binding domain of the *TP53* transcription factor [423,424,425,426,427]. Other regions of *TP53* prone to mutation include exon 4, which stabilizes the protein structure [428,429], and exon 10, which encodes the tetramerization domain of the protein [430,431]. *TP53* mutations are relatively common in CRC, occurring in approximately 50% of cases and are frequently observed in advanced-stage tumors [222,432,433]. Some mutations may result in a complete loss of function, while others either retain partial activity or acquire new functions that contribute to tumorigenesis [434,435]. Patients with *TP53*-mutated tumors have greater risks of disease recurrence, metastasis, and overall mortality compared to wild-type *TP53* tumors [436]. Other studies show that *TP53*-mutated tumors exhibit reduced responsiveness to certain treatments [437,438]. For example, *TP53*-mutated colon cells are associated with resistance to chemo-based drugs like fluorouracil (5-FU), which is commonly used in CRC treatment [438,439].

SMAD4 (also known as DPC4) is another tumor suppressor gene affected by genetic alterations in CRC. SMAD4 is an important downstream effector of the transforming growth factor-beta (TGF-β) signaling pathway where it inhibits cell proliferation and promotes apoptosis when the TGF-β pathway is intact [440]. SMAD4 mutations therefore disrupt TGF-β signaling, leading to uncontrolled cell growth and decreased apoptosis [441]. SMAD4 mutations have also been shown to impact Wnt signaling activity through a SMAD4 R361 hotspot mutation, which alters Wnt/β-catenin pathways and contributes to the evasion of growth suppressors in CRC cells [442]. Other studies have demonstrated that SMAD4 mutations are associated with increased CRC metastatic potential [442,443,444]. Mechanistically, loss of SMAD4 in CRC cells causes Bone Morphogenetic Protein (BMP) signaling, which enables a functional switch from tumor suppressive to metastasis promoting features through EMT and other metastasis-related processes [445]. These mutations have been also linked to resistance to certain chemotherapeutic agents commonly used in CRC treatment, such as 5-FU or folinic acid/fluorouracil/oxaliplatin regimen 4 (FOLFOX4) [446]. This drug resistance can be attributed to the altered cellular responses impacted SMAD4 mutation such as the PI3K/AKT/Cdc2 survival cascade [444,447].

While *APC*, *TP53*, and SMAD4 mutations play a significant role in CRC progression [448], it is important to understand that cancer is a multifactorial disease, and other genetic alterations or molecular mechanisms are also involved in evading growth suppressors through the override of cell cycle restriction points [449], enhancing DNA damage tolerance (DDT) mechanisms [450], and bypassing senescence to render immortality [451,452]. Therefore, targeting these mechanisms is a major focus of cancer research, and ongoing efforts are aimed at developing novel therapies to counteract the evasion of growth suppressors and improving the treatment outcomes [453,454,455]. Checkpoint mechanisms and its regulation in CRC are discussed below.

#### 4.2.4. Bypassing Cell Cycle Restriction Checkpoints

Cell cycle checkpoints are crucial mechanisms that regulate cell division processes and prevent the replication of damaged or abnormal cells, including cancerous cells [456]. However, in human cancers like CRC, these checkpoints can be bypassed, allowing cancer cells to divide and multiply unchecked (Figure 6) [449].

##### Cyclin-Dependent Kinase Inhibitors

In addition to *APC* and *TP53* that play important roles in maintaining genome integrity [449], cell cycle inhibitors such as cyclin-dependent kinase inhibitor 1 (CDKN1A) [457] and CDKN2A [458] also act as physiological brakes on the cell cycle, halting cell division and allowing time for DNA repair. CDKN1A (p21), a downstream gene target of *TP53* [459], blocks the activity of cyclin-dependent kinases (CDKs), which are enzymes that regulate the progression of the cell cycle [460]. CDKN2A, on the other hand, is located on the short arm of chromosome 9 (9p21) and encodes multiple proteins through alternative splicing, including p16INK4a and p14ARF [461,462,463]. The p16INK4a protein specifically inhibits the activity of cyclin-dependent kinases 4 and 6 (CDK4/6), which normally promote the progression of the G1 phase in the cell cycle [464]. By inhibiting CDK4/6, p16INK4a prevents the phosphorylation of retinoblastoma protein (Rb) and other target proteins, leading to cell cycle arrest and halting cell division [465]. The p14ARF protein, also known as alternate reading frame protein or ARF, also acts as a tumor suppressor by stabilizing *TP53* when cells undergo excessive proliferation or DNA damage [466,467].

In addition to the *TP53*-mediated impact of CDKN1A and CDKN2A activity, these genes have also been reported to undergo alterations in cancer tumors [468,469]. In CRC, the functional polymorphisms of CDKN1A may contribute to the risk of malignancy [470]. Meanwhile, CDKN2A mutation is relatively rare in CRC, but methylation of the p16 locus is common in both normal and cancerous colonic mucosa [471]. While promoter methylation of CDKN2A can lead to its low expression level, this alone does not show an independent association with the prognosis of cancer. However, this low expression has been shown to negatively affect CRC patients’ survival [472]. This could be because the loss of CDKN2A function inhibits cell cycle progression, promoting tumor growth [473]. The impact of this low expression could be more significant when combined with other factors and clinical stages [474,475,476]. More studies are needed to fully understand how CDKN2A promoter methylation interacts with other genetic alterations and clinical variables to influence CRC patients’ survival.

##### Aurora Kinases

The other protein family actively involved in cell cycle checkpoints is the Aurora serine/threonine kinases [477,478]. Aurora-A (AURKA) is involved in the regulation of centrosome function and spindle assembly, which are anatomically crucial for proper cell division [479]. Aurora-B (AURKB), on the other hand, is part of the chromosomal passenger complex (CPC), a “master controller” of the cell cycle [480]. It plays a role in almost every stage of mitosis, including the condensation, orientation, and segregation of chromosomes in addition to the formation of the spindle checkpoints, and cytokinesis [481,482,483,484]. Aurora B, along with other CPC proteins, ensure the proper segregation of chromosomes by destabilizing incorrect, erroneous kinetochore-microtubule attachments [481,485]. Both AURKA and AURKB are frequently overexpressed in CRC and associate with aggressive tumor behavior, poor prognosis, and resistance to chemotherapy [486,487]. Evidence indicates that *TP53* also can regulate the expression and activity of Aurora kinase in cancer cells [488,489]. Activation of *TP53* leads to the downregulation of Aurora kinases through p21-mediated CDK2/RB1/AURKA or revoking the inhibitory impact of miR-25 on FBXW7 as a negative regulator of AURKA and B, which helps maintain proper cell cycle control [489]. Accordingly, *TP53* knockdown in cancer cells reduces the level of p21, which in turn increases the activity of CDK2 [489]. This leads to the induction of Rb1 hyperphosphorylation and its dissociation with the transcription factor E2F3, which in return can bind to the AURKA gene promoter, potentiating AURKA gene expression [488]. On the other hand, AURKA can also phosphorylates *TP53* at Ser215/315 and facilitate its degradation [490,491]. Therefore, the impact of mutated *TP53* in cancers like CRC can be compounded by increased expression and activity of Aurora kinases, contributing to tumor progression and genomic instability [492].

##### Polo-like Kinases

The impact of *APC* on cell cycle progression can also be manifested through the regulated expression of the polo-like kinases (PLKs) and their activity [493]. PLKs are a family of serine/threonine kinases (PLK1–5) that play essential roles in cell cycle regulation, particularly during mitosis [494]. PLK1 localizes to the centrosomes and spindle poles during prophase and metaphase, and then relocates to the spindle midzone during late anaphase [495]. Expression of PLK1 is low in G0, G1, and S phases of the cell cycle, but then begins to increase during the G2 phase up to the M phase [496]. PLK1 activity is promoted through phosphorylation by CDK1 and AURKA, which help the PLK1 localization, activity, and substrate recognition during mitotic progression [497,498]. Normally, the Spindle Assembly Checkpoint (SAC) monitors the proper attachment of chromosomes to the mitotic spindle during mitosis [499]. When the SAC is activated due to improper attachment, it inhibits PLK1 activity and prevents premature mitosis [500]. PLK1 also controls several key transcription factors that promote cell proliferation, transformation, and EMT in various types of cancers, including CRC [501]. In fact, analysis of PLK1-depletion in CRC cells cultures and CRC mice models demonstrate a key role for PLK1 in colorectal carcinogenesis [502]. In this context, PLK1 overexpression in cancer cells is associated with poor prognosis and has been suggested as a potential target for cancer therapeutic interventions [503]. However, the role of PLK1 in cancer cells with deficient *TP53* or *APC* might be different [502,504]. For instance, in *TP53*-null cancer cells, the cell cycle sequence is more sensitive to PLK1 depletion than in *TP53*-wt cells [504]. On the other hand, in colon cancer cells expressing a truncated form of *APC* (*APC*-ΔC), PLK1 appears to have a tumor-suppressive function [502]. The inhibition of PLK1 in these genetically-modified cells weakens the mitotic suppressive action of PLK1, leading to accelerated mitotic exit and improved cell survival [502]. This suggests that PLK1 helps to maintain the mitotic checkpoint in these cells, and its inhibition can accelerate the development of adenomatous polyps, supporting a “tumor-suppressor function” for PLK1 in *APC*-ΔC-expressing colon cells [505]. Overall, the interaction between PLK1 and *APC* in cancer cells is complex and can have both tumor-promoting and tumor-suppressing effects, depending on the context. Further research is therefore needed to fully understand these dichotomous interactions and their implications for CRC treatment.

##### Checkpoint Kinases

Another group of cell cycle inhibitors are checkpoint kinases (CHKs), a family of serine/threonine kinases involved in the cellular response to DNA damage and replication stress [506]. CHK1 and CHK2 are the two main CHK family members involved in cell cycle regulation [507]. They act as “gatekeepers” and can be activated in response to DNA damage, such as double strand breaks or replication stress, where they subsequently phosphorylate and activate downstream effector proteins involved in DNA repair, cell cycle arrest, or apoptosis [508]. CHK1 is considered to have a stronger inhibitory effect on the activity of cell division control protein 25 (Cdc25) [509], whereas CHK2 is thought to have a larger role in inducing the expression of the G1–S transition inhibitor p21 [510]. Mutations or dysregulation of CHK1 and CHK2 can lead to genomic instability and to an increased risk of cancer development [506]. For instance, overexpression of CHK1 and CHK2 promote cancer cell resistance to radiation or chemotherapies by enhancing their ability to repair induced DNA damage [511,512,513]. In the context of CRC, frameshift mutations in *CHK1* with microsatellite instability, suggest that CHK1 alterations could represent an alternative way for cancer cells to escape cell cycle control [514]. On the other hand, studies show that CHK2 expression levels CRC cases show an approximately 50% reduction, which may contribute to the development of colorectal neoplasm [515,516]. In line with these findings, knockdown of CHK1 expression sensitizes human colon carcinoma cells to DNA-damaging agents, while suppression of CHK2 had no impact on CRC cells [517]. Specifically, it appears that inhibition of CHK1, but not CHK2, caused a greater abrogation of the G2 phase by DNA-damaging treatments and a greater sensibility to the same treatments in CRC cells characterized with *TP53* and p21 wild-type proteins [518]. However, whether CHK1 inhibition can also be exploited for therapy of *TP53*-wild-type cancers remains ambiguous [519,520]. Some studies demonstrate a synergy between *TP53* deficiency and CHK1 inhibition [519], while others indicated that *TP53* status is only one of the decisive factors [520,521]. For example, CHK1 abrogation together with *TP53* inactivation in *TP53*-mutated B-lymphoid cells can result in uncontrolled proliferation leading to direct apoptosis or mitotic catastrophe [519]. Accordingly, a synthetic lethal relationship between CHK1 inhibition and *TP53* deficiency has been observed in soft-tissue sarcomas, but not *TP53*-WT undifferentiated pleomorphic sarcoma (UPS) models, which was associated with an increased proportion of cells with DNA damage [522]. In lung cells, inhibition of CHK1 had a strong effect on *TP53* and p21 dynamics, where CHK1 phosphorylation level was high [523]. In contrast, CHK1 inhibition had almost no effect on *TP53* and p21 dynamics in breast cells, where CHK1 phosphorylation level was low [523]. Additionally, it was found that p21, both basal and *TP53*-induced pools, protects normal epithelial cells and colorectal tumors from the lethal effects of DNA damage as a single stress or in combination with CHK1 inhibition [519]. This suggests that p21 attenuators may sensitize tumors, independent of their *TP53* status, to the lethal effects of DNA damage combined with CHK1 inhibition [519]. While these findings highlighted the impact of CHKs deregulation in CRC development, they also suggest that the specific downstream effects vary depending on the cell type and that more research is needed to fully understand these networks [14].

##### WEE1

WEE1 is another kinase that plays a crucial role in the cell cycle, particularly in the G2/M transition [524]. It functions as a G2 checkpoint regulator by directly phosphorylating and inhibiting Cdc2, the major cyclin-dependent kinase inducing G2-M progression in the cell cycle [525]. This inhibitory action prevents the transition from the G2 phase to the M phase during the cell cycle, ensuring that no DNA damage exists prior to cell division [525]. In the context of cancer, WEE1′s role in cell cycle regulation has been shown to promote cell survival in various types of malignancies, including breast cancer [526,527], leukemia [527,528], melanoma, brain tumors [529], and CRC [530]. Expression of WEE1 in CRC appears to be variable and may be influenced by several factors [530]. For instance, a study found that WEE1 was positive in 52.9% of patients with CRC, which is lower than the positive rate of WEE1 in melanoma and vulvar squamous cell carcinoma tissues [531]. In terms of clinical significance, WEE1 protein staining scores were found to be significantly linked with distant metastasis of CRC and high TNM staging [531]. Therefore, inhibition of WEE1 has been suggested as a potential strategy for cancer therapy, especially in combination with DNA damaging agents [532]. This is because cancer cells often show elevated replication stress, which likely provides sensitivity to WEE1 inhibitors [533]. Furthermore, loss of the G1 checkpoint is frequent in tumors and potentially provides increased reliance on the G2 checkpoint [534], thereby selectively sensitizing cancer cells to checkpoint inhibitors like adavosertib (AZD1775), which is a highly selective inhibitor of WEE1 [535,536,537,538]. For example, microRNAs miR-424 and miR-503 have been found to directly regulate WEE1 leading to a significant decrease in both mRNA and protein expression levels of WEE1 [535]. Accordingly, a lower level of tumor suppressor miR-424/503 has been previously reported in several types of cancer, including CRC [535,539,540,541,542]. In fact, WEE1 expression in ovarian cancer stem-like cells could be resorted by transcription factor NANOG via modulating the negative impact miR-424/503 on WEE1 transcripts [535]. Furthermore, this mechanism was shown to be neutralized in the same model, under atorvastatin stimulation [535]. NANOG activation has been associated with reduced chemosensitivity and poor survival outcome in CRC patients [543]. Moreover, cancer cells transduced with *shRNA* against NANOG failed to form visible or microscopic hepatic liver colonies, compared with parental cells in a mouse model of CRC [544]. These findings suggest that NANOG may play a role in the regulation of WEE1 in certain types of cancer such as CRC, potentially through the modulation of microRNAs [545,546]. However, there are still outstanding questions regarding the use of WEE1 inhibition as an anticancer strategy [529,547,548]. These include determining the optimal timing of treatment with the WEE1 inhibitor and DNA-damaging components of chemotherapy, understanding the impact of WEE1 inhibition on the genomic integrity of normal cells and tissue, and whether WEE1 inhibition can sensitize CRC cells to DNA-damaging agents [529,547,548].

##### Protein Phosphatase-1

In addition to above cell cycle mediators, there are other proteins which despite their noticeable impact on cell cycle regulation, have not been well-studied in human cancers, in particular CRC [549,550,551]. For example, protein phosphatase-1 (PP1) is a serine/threonine phosphatase involved in the regulation of various cellular processes, including cell division, proliferation, and differentiation [549]. It is generally considered a tumor suppressor in cancer [552,553]. PP1 exerts its tumor-suppressive effects by dephosphorylating various signaling proteins involved in cell cycle control, apoptosis, and DNA repair [554,555,556,557]. PP1 plays a role in the transition from the G1 phase (the period before DNA synthesis) to the S phase (DNA synthesis phase) [553]. It dephosphorylates and inactivates the Rb protein which is a negative regulator of the G1 to S transition [553,558,559]. By dephosphorylating Rb, PP1 promotes the activation of E2F transcription factors, allowing the cell to enter the S phase [559]. PP1 also regulates the activity of the CDK complexes that control DNA replication [560,561,562]. During mitotic entry, PP1 dephosphorylates and inactivates CDK1/cyclin B complexes, allowing the cell to progress from the G2 phase to mitosis [561,562]. In mitotic exit, PP1 is involved in the dephosphorylation of various substrates, including the Securing complex (a protein complex involved in chromosome segregation), kinetochore proteins, and the nuclear envelope, which facilitate chromosome segregation and the reformation of the nuclear envelope [563,564,565]. PP1 also plays a role in the inactivation of SAC by dephosphorylation and counteraction the activity of kinases involved in the SAC action, allowing the cell to progress to anaphase once all chromosomes are properly aligned on the spindle [566,567]. Therefore, PP1 helps maintain genomic stability and prevents the formation of cancerous cells [568]. PP1 lacks substrate specificity and depends on over 200 regulatory proteins to confer specificity towards distinct substrates [569]. Most of these regulatory proteins are intrinsically disordered proteins (IDPs) that interact with PP1 through pre-formed secondary and tertiary structures [570]. The interaction of PP1 with regulatory subunits leads to a pronounced reshaping of the catalytic cleft of PP1, contributing to the increased substrate specificity of the complex [571]. In the context of cancer, the protein phosphatase activity of *PTEN*, a protein that shares similar functions with PP1 [572], has been found to negatively regulate the SRC-mediated drug-resistant signaling pathway [573]. This suggests that PP1 and similar proteins may play a role in cancer progression and resistance to treatment [574]. Conversely, dysregulation of PP1 activity can lead to aberrant activation of Wnt/β-catenin signaling and the PI3K/AKT/mTOR, MAPK, and AMPK pathways, and eventually promotes tumor growth and progression. It can also dephosphorylate and inactivate AURKA at T288 residue [575]. Low expression of PP1 along with spinophilin has been reported to correlate with poor prognosis, increased tumor aggressiveness, and reduced patient survival rates in lung cancer [576]. While deregulation of PP1 has not been studied in CRC yet, it has been shown that colorectal tumors from patients with an increased levels of PPP1R11, a regulatory subunit of PP1, directly associated with *TP53* mutations and metastasis to liver [577]. On the other hand, correlation analysis of PP1 and DARPP-32, which involves in cancer cell survival and drug resistance [578], depicted that that low expression of PP1 in samples with a higher level of DARPP-32 associated with adverse survival in breast cancer patients when compared to high expression in the same group [579]. These findings suggest that low expression of PP1 may be associated with adverse outcomes in certain types of cancer, but more research is needed to understand the specific role of PP1 in CRC.

##### Mitotic Arrest Deficient Protein-2

Mitotic arrest deficient protein 2 (MAD2) is another key component of the cell cycle checkpoint machinery that ensures the accurate separation of chromosomes during cell replication [550,551]. Similar to PP1, MAD2 also plays a crucial role in SAC regulation [580]. When the chromosomes are unattached or incorrectly attached, MAD2 becomes activated and forms a complex with other proteins at the kinetochores, which are specialized protein structures on the chromosomes that bind to microtubules of the spindle apparatus [581]. The formation of this complex generates a signal that inhibits the anaphase-promoting complex/cyclosome (*APC*/C), a large E3 ubiquitin ligase that targets key mitotic regulators for degradation by the proteasome and responsible for promoting anaphase onset [582]. Inhibition of *APC*/C prevents the degradation of securing and cyclin B, which are necessary for the cell to progress to anaphase [583,584]. Dysregulation of MAD2 levels, either by upregulation or downregulation, can result in similar genomic aberrations and contribute to decreased patient survival [585]. High MAD2 levels are associated with increased risk of all-cause death and cancer recurrence in non-ovarian cancers [585]. In CRC, a significant decrease in the levels of SAC proteins such as Bub1/R1, Mad1/2, and AURKB, along with the *TP53* oncoprotein, has been reported by Twist1 overexpression that shows their collective role in regulating chromosomal stability in cancer cells [586]. MAD2 might also be related to advanced stages of cancer since its overexpression has been shown in thyroid carcinoma with an aggressive nature [587]. Meanwhile, MAD2-silenced cells showed a reduced viability, suggesting this protein as one of the most important effectors of CMLD-2-induced cell growth decrease [587]. In conclusion, while MAD2 plays a significant role in cancer progression and prognosis, its specific role in CRC has not been explicitly detailed yet. Therefore, more research might be needed to establish this relationship.

#### 4.2.5. Endurance of DNA Damages

Like all cancers, CRC can result from various genetic mutations, including DNA damage [588]. Therefore, these cells have developed several mechanisms to counteract DNA damage, which contributes to their survival and resistance to treatment [588] (Figure 6). Various oncogenic signaling molecules regulate these DNA repair mechanisms [587]. For instance, the forkhead box protein M1 (FOXM1) is a transcription factor involved in the regulation of various cellular functions, including DNA damage response, cancer stem cells, and cell cycle regulation [589,590,591,592]. It transcriptionally regulates most of the DNA damage response proteins that are essential for normal cell survival [589]. However, the overexpression of FOXM1 in cancer cells can lead to chemoresistance, as FOXM1 enhances DNA repair damaged by these drugs, thereby reducing their effectiveness [590,591,592]. In addition, FOXM1 expression has been found to be upregulated in CRC tissues, and its expression level is negatively associated with the sensitivity of CRC cells to the chemotherapeutic agent 5-FU [593]. This suggests that silencing FOXM1 may play a role in overcoming chemoresistance, and invasiveness of CRC cells [594].

The next DNA damage tolerance mechanism is aneuploidy or the presence of an abnormal number of chromosomes in a cell [595]. As a hallmark, cancer cells like CRC have adopted mechanisms to cope with the detrimental consequences of aneuploidy, including different responses to cellular stresses, immune system activation, and cell cycle arrest [596]. For example, they may upregulate heat shock proteins and other molecular chaperones to cope with proteotoxic stress or alter their metabolism to deal with metabolic stress [597,598,599]. Aneuploidy incidence could increase with the size of colorectal adenomas, and adenomas with higher degrees of aneuploidy are more likely to progress to cancer [600,601]. Accordingly, aneuploid CRC tumors have greater allelic loss and are associated with poor differentiation of the carcinomas, but not with distant metastasis [602].

TRIM31 upregulation is another mechanism cancer cells use to counteract DNA damage induced by radiation [603]. The biological effects of radiation, such as cell death and redistribution of the cell cycle, involve many pathways, especially DNA damage repair pathways [604,605,606]. TRIM31 may be involved in these pathways through its interaction with ATM, a protein that plays a key role in the cellular response to DNA damage [607]. Accordingly, enhanced level of TRIM31 promoted invasion and metastasis in CRC cells [608]. In contrast, knockdown of TRIM31 led to increases in ROS production, an aggregation of DNA damage, and radiosensitivity in CRC cells [607]. Therefore, patients with lower expression of TRIM31 have better response to preoperative radiotherapy [607].

Alterations in MMR and MSI mechanisms have also a significant impact on DNA damage tolerance in CRC cells [609]. MMR is a critical DNA repair system that corrects errors (like base-base mismatches and insertion-deletion loops) that occur during DNA replication [610,611]. Defects in MMR genes can lead to MSI, a hypermutable phenotype characterized by lengthy alterations within short repetitive DNA sequences [612]. In CRC, high level of MSI is associated with a distinct clinical and pathological phenotype, including proximal tumor location, poor differentiation, and abundant tumor-infiltrating lymphocytes [613,614,615]. Importantly, MSI-H tumors are generally more resistant to chemotherapy that induces DNA damage, such as 5-FU [53,616], but have a better overall prognosis compared to microsatellite stable (MSS) tumors [617,618,619]. The resistance to chemotherapy in MSI tumors is thought to be due to the increased ability of these cells to tolerate DNA damage [620,621]. The loss of MMR function in cancer cells allows them to accumulate mutations without triggering apoptosis, leading to the survival of cells that would otherwise be eliminated [620,621]. This increased DNA damage tolerance can drive tumor progression and contribute to the development of resistance to DNA-damaging agents [622]. However, while MSI-H CRC tumors are generally more resistant to certain types of chemotherapy, they may be more susceptible to immune checkpoint blockade therapy, which has shown promising results in MSI-H metastatic CRC [207,623]. In conclusion, MSI and MMR alterations can increase DNA damage tolerance in CRC cells, influencing their response to therapy and overall disease progression [624,625]. However, the exact impact can vary depending on the specific genetic context and the types of therapy used [624,626].

#### 4.2.6. Evading Cell Senescence Mechanisms

Cell senescence refers to a state in which cells cease to divide and enter a state of irreversible growth arrest [627]. The two main mechanisms through which cell senescence can be induced are replicative senescence [628] and premature senescence [629]. Replicative senescence is related to the limited replicative capacity of somatic cells [630]. During each cell division, the telomeres, which are protective caps at the ends of chromosomes, become shorter [631]. Eventually, when telomeres reach a critical length, the cell’s ability to divide is halted, and it enters replicative senescence [632]. This process acts as a cell division counting mechanism and is often referred to as the “Hayflick limit”, named after Leonard Hayflick, who first observed this phenomenon [633]. Premature senescence, on the other hand, is triggered by various stress signals that can damage the cell’s DNA, activate oncogenes, or induce oxidative stress [634,635,636,637]. Premature senescence can occur independently of telomere shortening and does not have a strict limit on the number of cell divisions [638]. Instead, it is induced by specific stresses that the cell experiences [638].

In cancer, both replicative [639,640,641,642] and premature senescence [643,644,645] can play significant roles. As cancer cells divide rapidly, their telomeres can become shortened [646]. To bypass replicative senescence and continue dividing, cancer cells often activate telomerase or other alternative lengthening of telomeres (ALT) mechanisms [647]. By maintaining telomere length, cancer cells can evade the normal limitations on cell division, contributing to tumor growth and progression [648]. Premature senescence can also be induced in cancer cells in response to various stressors, including chemotherapy and radiation therapy [649]. Therefore, this cellular response serves as a tumor-suppressive mechanism by arresting the growth of damaged cells [650]. It is a strategy used in cancer treatments to halt the proliferation of cancer cells and promote their clearance by the immune system [643].

Despite the advances made over CRC growth and development mechanisms, unraveling the processes that allow CRC cells to bypass senescence continues to be a complex and challenging field of study [651]. It has been shown that genes involved in DNA replication are significantly deregulated in colorectal tumors, and that overexpression of certain replication genes could be associated with poor patient survival [652]. Similarly, loss of *TP53* function may be a selection pressure for escaping replicative senescence in many human cancers, including CRC (Figure 6) [653]. As for the premature senescence (induced by various factors such as DNA damage [654], oxidative stress [655], and certain drugs [656]), it seems that the deficiency of Caveolin-1, a protein involved in various cellular processes, could be a key factor in CRC cell death through activation of the *TP53*-p21 pathway, a well-known regulator of cell cycle progression and senescence [657]. On the other hand, Teng-Long-Bu-Zhong-Tang (TLBZT), a traditional Chinese medicine, could enhance the effects of 5-FU in colon carcinoma, provoke apoptosis or cell senescence, and inhibit angiogenesis in colon carcinoma [658]. Future studies revealed that TLBZT induces cell senescence in cancer cells by regulating the levels of p21 and p16, and inhibiting the phosphorylation of Rb, ultimately leading to cell cycle arrest and potential anticancer effects [659,660]. Accordingly, it has been shown that low concentrations of camptothecin, a drug that induces DNA damage, enhanced cell cycle arrest and premature senescence in human CRC cells, while high concentrations induced apoptosis [661]. The anticancer effects of camptothecin in cancer cells are mediated through senescence induction via ATM/CHK2/*TP53*/p21 pathway and blocking autophagy via AMPK/TSC2-mTOR inhibition axis [662]. In the context of clinical prediction and outcome, senescence has been shown to be a good treatment response indicator in metastasized CRC patients [663]. Later, in 2022, a study led by K. Dong et al. developed a senescence-related prognostic signature to predict the prognosis and immunotherapeutic response of patients with CRC [664]. This model can also potentially identify drug targets and aid in guiding PD-1 (programmed death-1) immunotherapy [665]. Collectively, these studies suggest that both replicative and premature senescence can play a role in the development and progression of CRC, and that inducing these processes could represent potential therapeutic strategies for this disease [664,666]. However, more research is needed to fully understand senescence mechanism and its implications for cancer treatment [667,668].

Taken together, these mechanisms allow CRC cells to continue growing and dividing even in the presence of signals that would normally restrain growth, restrict cell cycle progression, or induce cell death in response to DNA damage [669,670]. As research advances, scientists are continuously identifying potential therapeutic targets to interfere with these mechanisms and to develop more effective treatments for CRC [671,672,673]. Targeted therapies, immunotherapies, and combination treatments are some of the strategies being explored to improve the outcomes for patients with CRC [674,675].

### 4.3. Resisting Cell Death

Cancer cells possess a remarkable ability to circumvent cell death mechanisms and survive harsh conditions within the tumor microenvironment [676,677,678,679]. Faced with diverse stresses from DNA damage, limited resources, and anticancer therapies, tumor cells have evolved diverse strategies to resist demise (Figure 6) [677,680,681]. Apoptosis resistance, achieved through defects in key tumor suppressors and activated pro-survival pathways, is the most prominent survival strategy [682,683], but not the only one. Cancer cells can also block alternative cell death modes like necrosis and ferroptosis [684,685]. Moreover, they induce pro-survival processes like autophagy to evade death and fuel continued growth [684]. This multi-pronged approach to circumventing cell death allows cancer to thrive despite the myriad stresses it encounters [684]. Overcoming apoptosis resistance as well as nonapoptotic death mechanisms has therefore become a major focus of cancer research to enhance the efficacy of current therapies [684,686,687].

#### 4.3.1. Mechanisms of Intrinsic Apoptosis Resistance

CRC cells frequently have defects in apoptotic pathways that allow the cancer to develop and progress (Figure 6) [688,689,690,691,692]. A key pathway hijacked is intrinsic apoptosis, as CRC tumors encounter various intrinsic stresses in the tumor microenvironment [693]. Hypoxia, or low oxygen levels, is common within the dense tissue of solid CRC tumors [694,695]. Studies have shown that hypoxia activates the YAP oncogene, which in turn upregulates the expression of the anti-apoptotic protein Bcl-xL [696,697]. Bcl-xL works to block the intrinsic pathway by binding pro-apoptotic effectors like Bax and Bak [698], preventing their oligomerization and the release of cytochrome c from mitochondria [699]. This inhibition of the intrinsic apoptotic cascade allows CRC cells to evade cell death even under hypoxic conditions, promoting tumor survival and growth [700].

Other signals produced within the CRC tumor microenvironment also dysregulate intrinsic apoptosis. Inflammatory cytokines from immune cells in the tumor have been found to increase levels of the anti-apoptotic Bcl-2 protein in intestinal epithelial cells [701,702]. Overexpression of Bcl-2 and Bcl-xL helps CRC cells bypass intrinsic apoptotic stimuli to avoid mitochondrial outer membrane permeabilization (MOMP) and caspase activation [703,704,705]. Mutations that commonly occur in CRC, such as Wnt pathway activation [691], indirectly influence anti- versus pro-apoptotic Bcl-2 protein expression as well [706]. Overall, dysregulated expression of anti-apoptotic Bcl-2 family members is a major strategy CRC uses to acquire resistance to intrinsic apoptosis and ensure genetically unstable cells persist [707]. This eventually also allows CRC progression through the accumulation of additional mutations [708,709].

#### 4.3.2. Evading Extrinsic Apoptosis

The extrinsic apoptosis pathway triggers cell death through activation of cell surface death receptors (Figure 6) [710]. These receptors belong to the tumor necrosis factor receptor superfamily and include Fas, TNFR1, and the TNF-related apoptosis inducing ligand (TRAIL) receptors TRAILR1 and TRAILR2 [710,711]. Upon binding of their respective ligands, these death receptors recruit an adaptor molecule called FADD to their intracellular death domains [712,713]. FADD contains both a death domain that interacts with the activated receptor, as well as a death effector domain [714], which enable the recruit of procaspases-8 and -10 to the activated receptor complex [714]. The clustering of FADD and procaspase molecules forms a multi-protein structure called the death-inducing signaling complex (DISC) [714]. Within this DISC, procaspase-8 is brought into close proximity which allows for self-activation through induced proteolytic cleavage [715,716]. Active caspase-8 can then directly activate downstream effector caspases such as caspase-3, rapidly inducing apoptosis independent of mitochondrial outer membrane permeabilization [717]. In some cell types, caspase-8 may also trigger the intrinsic pathway through cleavage of Bid and mitochondrial involvement [718,719].

As with the intrinsic pathway, CRC cells often develop defects in the extrinsic pathway that promote evasion of apoptosis [720]. One way is via mutation of death receptors, such as Fas, that normally initiate the extrinsic apoptotic cascade upon engagement with death ligands [710]. Another mechanism utilized by CRC cells is downregulation of death ligand expression [686,688,721]. TRAIL shows promise as a death ligand that triggers extrinsic apoptosis through DR4 and DR5 receptors [722,723]. However, studies have found that TRAIL and its receptors are often downregulated in CRC tumors compared to normal tissue [65]. This reduction in TRAIL and its receptors decreases the sensitivity of CRC cells to TRAIL-mediated extrinsic apoptosis [724,725,726].

IBD disorders, such as UC, that are associated with increased CRC risk have also been linked to decreased death ligand/receptor systems [727]. UC specifically has been shown to upregulate expression of the decoy receptor DcR3 [728]. DcR3 competitively binds the death ligands FasL and TRAIL without initiating the apoptotic signaling cascade [728]. This sequesters the death ligands and reduces the ability of Fas and TRAIL receptors to trigger extrinsic apoptosis when engaged [729,730]. Overall, CRC cells employ various strategies such as death receptor mutations, downregulation of death ligands and receptors, and upregulation of decoy receptors to circumvent triggering of the extrinsic apoptotic pathway and promote the cancer progression and growth [723,731,732,733,734,735].

#### 4.3.3. Avoiding Non-Apoptotic Cell Death

Necroptosis is a regulated form of necrotic cell death triggered by death receptors like TNFR1 [736]. Upon TNFR1 ligation, the complex I machinery recruits RIPK1 and RIPK3 kinases to initiate the necroptotic signaling cascade [736]. CRCs develop resistance to this pathway through genetic and epigenetic changes that disrupt core components [737,738]. Frequent mutations in CRC directly silence or downregulate expression of RIPK1 and RIPK3 [739,740]. This prevents the critical phosphorylation events driven by RIPK kinases that activate downstream molecules like MLKL [741,742]. MLKL normally drives necrotic plasma membrane rupture, a defining feature of necroptosis [743]. Without RIPK1/RIPK3 signaling, necroptotic execution is effectively blocked in CRC cells [743].

Additional resistance can also occur via epigenetic mechanisms [744,745]. CRC tumors exhibit promoter hypermethylation of RIPK1, reducing its transcription [746]. CRC cell lines also secrete factors that sponge TNF, inhibiting death receptor stimulation of necroptosis [744]. Together, these adaptations allow CRC cells to circumvent controlled necrotic demolition via death receptors to persist even under conditions conducive for necroptotic cell death [744,745]. Overall, disabling the RIPK-dependent necroptotic pathway is a key strategy CRCs use to resist this non-apoptotic cell fate [686,745].

On the other hand, autophagy is a cellular process that involves the degradation and recycling of damaged or dysfunctional cellular components, such as organelles and proteins [747]. The word “autophagy” comes from the Greek words “auto” (self) and “phagy” (eating), which together mean “self-eating” [748]. Autophagy is initiated upon cellular stress through the ULK1/Atg13/FIP200 complex [749]. This normally activates the class III phosphatidylinositol 3-kinase (PI3K) complex containing *Beclin-1*, which nucleates formation of the autophagosome isolation membrane [750]. However, CRCs commonly mutate or delete the *Beclin-1* gene, disrupting PI3K complex assembly and function [751]. Without *Beclin-1*, autophagosomes cannot efficiently engulf damaged cargo like dysfunctional mitochondria and protein aggregates [752]. Studies have shown impaired autophagosome biogenesis and accumulation of autophagic vesicles in CRC models lacking *Beclin-1* [753,754]. Additional resistance occurs via overexpression of p62/SQSTM1, which not only binds ubiquitinated aggregates, but also interacts with LC3 to target them for autophagic degradation [692,755]. High p62 in CRC outcompetes protein aggregate binding to LC3, preventing autophagic turnover [692,755]. Collectively, mutations impacting *Beclin-1* and upregulation of p62 sabotage proper autophagic flux in CRC cells [754]. This allows sequestration of worn-out or stressed organelles to support biosynthesis and bioenergetics promoting persistent neoplastic growth [754,756].

Finally, ferroptosis is characterized by lethal lipid peroxide accumulation resulting from iron-dependent Fenton reactions [756,757]. These reactions produce highly reactive lipid ROS that normally trigger regulated necrotic cell death to eliminate damaged cells [758]. CRC cells frequently undergo dysregulation in tumor suppressor genes like Keap1 that derepress Nrf2 signaling [759,760,761,762,763]. As a downstream target of Nrf2, GPX4 expression is significantly increased at both mRNA and protein levels in CRCs [764,765,766]. GPX4 is the key enzymatic regulator of lipid peroxidation, directly reducing toxic lipid hydroperoxides to halt ferroptotic execution [767]. Studies show GPX4 overexpression alone might be sufficient to confer complete resistance to ferroptotic inducers in CRC cell lines [768,769,770,771,772]. Additional findings indicate GPX4 is also epigenetically upregulated in CRC through histone modifications at its promoter [773]. High GPX4 then potently scavenges lipid ROS to circumvent iron-dependent cell death, even under conditions of oxidative and ER stress that would normally trigger ferroptosis [774]. Together, these GPX4-centered adaptations allow CRC tumors to evade this physiological form of regulated necrosis and continue thriving despite aberrant accumulation of iron and oxidative damage [774,775,776].

### 4.4. Deregulating Cellular Energetics and Metabolism in CRC

Cellular metabolism is tightly regulated to maintain energetic and anabolic homeostasis [777]. In normal cells, glucose and oxygen are broken down through sequential biochemical pathways to efficiently generate energy through oxidative phosphorylation [778]. The citric acid cycle and electron transport chain fully oxidize nutrients to fuel mitochondrial ATP production [778]. Cell proliferation is precisely controlled by metabolic and growth signaling networks [779]. However, cancer cells undergo metabolic reprogramming to support rapid uncontrolled growth (Figure 6) [679]. The disruption of metabolic regulation can be achieved by oncogenic mutations, hypoxia, and other microenvironmental cues [780]. For example, oncogenic BRAF mutations lead to metabolic alterations in less than 10% of CRC cells [781]. A common feature is increased aerobic glycolysis despite under normoxic conditions, known as the “Warburg effect” [782,783]. This heavy reliance on glycolysis allows cancer cells to shunt metabolic intermediates towards biomass generation through pentose phosphate and other anabolic pathways [783,784]. CRC exemplifies dramatic metabolic alterations that fuel tumor progression [785,786]. For example, CRC cells exhibit increased glucose transport and expression of glycolytic enzymes [785,787]. They also depend more on glutamine and beta-oxidation of fatty acids [788,789,790]. These adaptations generate precursors for macromolecule biosynthesis essential for CRC cell proliferation [788,789,790]. Oncogenic *KRAS* mutations in CRC activate signaling cascades that cement the metabolic switch towards glycolysis [785,791,792].

Herein, we will discuss key aspects of how metabolic reprograming supports CRC, including the basis of the Warburg effect and the impact of specific dysregulated enzymes on downstream pathways governing CRC pathology. Systems that feedback to further enhance metabolic flexibility in CRC will also be covered. Finally, the targeting of metabolic vulnerabilities for CRC therapeutic development will be presented along with their challenges.

#### 4.4.1. The Warburg Effect

The Warburg effect plays a crucial role in supporting the rapid growth and progression of CRC through molecular reprogramming of cancer cell metabolism [793]. A primary driver is the presence of oncogenic *KRAS* mutations that occur in approximately 50% of CRC cases [794]. Mutant *KRAS* directly activates downstream signaling pathways like RAF/MEK/ERK and PI3K/AKT, even under normoxic conditions within the tumor microenvironment [795]. A key consequence is the stabilization and accumulation of the α-subunit of hypoxia-inducible factor 1 (HIF1α), a major transcription factor that regulates the cellular response to low oxygen levels [796]. However, in CRC, elevated HIF1α resulting from oncogenic *KRAS* signaling induces transcriptional upregulation of numerous glucose transporters and glycolytic enzymes under normoxia [780,797]. This includes increased expression of GLUT family members that import more glucose into tumor cells, as well as rate-limiting enzymes like hexokinase 2 (HK2) and lactate dehydrogenase A (LDHA) [781,798]. Collectively, these molecular alterations driven by mutant *KRAS* promote aerobic glycolysis, known as the Warburg effect, as the dominant metabolic program in CRC cells to support their insatiable energetic and biosynthetic demands of uncontrolled growth and proliferation [794].

By accelerating aerobic glycolysis, or the Warburg effect, through molecular changes driven by mutant *KRAS*, CRC cells shift away from using the more efficient oxidative phosphorylation pathway to generate ATP [679,784]. Although glycolysis produces ATP at a lower rate, this metabolic reprogramming allows CRC cells to divert a greater portion of glycolytic intermediates into ancillary pathways that fuel biosynthesis [679,799,800]. A key example is the pentose phosphate pathway, into which glucose-6-phosphate can enter after being phosphorylated by hexokinase [679]. Overexpressed enzymes in CRC cells like phosphofructokinase and pyruvate kinase M2 further flux carbon through glycolysis [679,785,801]. Sustained aerobic glycolysis, or the Warburg effect, meets the high energy and anabolic precursor demands required for cancer cells to rapidly grow and divide [784,802]. Excess lactate produced is exported from CRC cells by monocarboxylate transporters, acidifying the microenvironment in a manner that supports invasion, metastasis, and evasion of antitumor immunity through immune suppression [803,804,805,806].

The reliance on aerobic glycolysis, or the Warburg effect, provides CRC cells significant metabolic plasticity and flexibility [807]. This supports their ability to adapt to different tissue microenvironments during metastasis [678]. For example, CRC liver metastases demonstrate the capability to relatively increase glutamine metabolism and glutaminolysis compared to primary colon tumors [808]. Such metabolic reprogramming facilitates colonization at secondary sites [809,810]. Non-invasive FDG-PET/CT imaging capitalizes on altered FDG glucose uptake by CRC tumors to serially monitor treatment response patterns [811,812]. Preclinical studies have shown that directly targeting enzymes causal to aerobic glycolysis, such as hexokinase and lactate dehydrogenase, can inhibit CRC progression both alone and synergistically with chemotherapy [781,813]. This underscores glycolysis as an exploitable metabolic dependency downstream of oncogenic *KRAS* signaling that fuels CRC development and aggressiveness [679,784]. Overall, extensive reprogramming of central carbon metabolism drives the progression of this malignancy by meeting heightened energetic and biosynthetic needs.

#### 4.4.2. Dysregulated Glucose and Glutamine Metabolism in CRC Cells

In addition to the Warburg effect driving increased glycolysis, CRC cells also exhibit dysregulated metabolism of the amino acid glutamine to support tumor growth and survival (Figure 6) [814]. Glutamine is taken up via increased expression of transporters like SLC1A5, then converted to glutamate by elevated glutaminase isoform GLS2 [815,816,817]. This drives the entry of glutamine-derived carbons into the Krebs cycle (also known as citric acid cycle or tricarboxylic acid cycle/TCA) via α-ketoglutarate [818,819]. Around 10% of CRCs also exhibit mutated *IDH1*, generating NADPH from α-ketoglutarate to maintain redox balance during rapid growth [819]. Glutamate can be further metabolized in the mitochondria to fuel ATP production or translocated to the cytosol for biosynthesis of molecules like glutathione, non-essential amino acids, and nucleotides [820,821,822]. GLUT transporters also import high intracellular levels of glucose to feed glycolysis and produce lactate [823,824]. However, glucose-derived pyruvate can enter the TCA cycle, and intermediates from both nutrients converge at oxaloacetate to mutualistically support anabolism [825,826,827].

Metabolic reprogramming exhibited by CRC cells confers significant flexibility in their utilization of key carbon and nitrogen sources [828,829]. Rewired glucose and glutamine metabolism pathways allow tumors to adjust relative reliance on these nutrients depending on environmental conditions [830,831,832,833]. For example, proliferating CRC cells may increase glutamine metabolism and decrease their dependence on glucose uptake under normoxic conditions in certain tissues like the liver, employing glutaminolysis to better support aerobic proliferation [816,834,835]. The many biosynthetic roles of glutamine in CRC particularly involve generation of non-essential amino acids and collagen, a major component of the extracellular matrix [788,836,837]. Heightened collagen production facilitates CRC cell invasion into surrounding stroma and the formation of metastases [838]. Non-invasive analytical techniques like using 13C-labeled glucose [839] and glutamine [840] have begun to elucidate how nutrient flux is altered in individual patient tumors. Such emerging metabolomic profiles could complement genomic analyses to stratify CRC subtypes and predict response to targeted inhibitors [841]. In this regard, dual blockade of glutaminolysis and glycolysis, as shown preclinically with combined glutaminase and hexokinase inhibition, holds promise for comprehensively restricting the multiple nutrient supply lines exploited by CRC to fuel uncontrolled growth, survival, and dissemination [842].

#### 4.4.3. Role of Oncogenic Drivers’ Mutations in Reprogramming Metabolism in CRC

Mutant *KRAS* is a primary oncogenic driver that rewires cellular metabolism in CRC through MAPK pathway activation (Figure 6) [794,843]. *KRAS* signaling leads to chronically elevated ERK1/2 downstream, even under normoxia within tumors [844]. This stimulates the expression of HIF1α and other hypoxia-responsive factors normally [845,846]. HIF1α then induces transcriptional upregulation of numerous glycolytic enzymes and GLUT transporters [847]. Constitutive MAPK signaling also feeds into mTORC1 to promote anabolic processes [848]. The metabolic effects of mutant *KRAS* are further compounded if concurrent PI3K pathway mutations occur in PI3K, *PTEN*, or AKT genes [849,850,851]. Activated PI3K/AKT then further enhances HIF1α activity, HK2 and LDHA expression, and glutaminolysis [785,852,853]. mTORC1/2 signaling emanating from PI3K deregulation also drives lipogenesis and biosynthesis [854,855].

The concomitant activation of MAPK and PI3K/AKT pathways downstream of common *KRAS* and PI3K mutations has profound cooperative effects on rewiring central carbon metabolism in CRC cells [856]. Both pathways converge on promoting HIF1α activity and transcriptome changes that boost glycolysis, glutaminolysis and nutrient transport [830,852,857,858]. Mutant *KRAS*-driven MAPK signaling stimulates expression of numerous glycolytic enzymes and GLUTs via ERK/HIF1α signaling [859,860]. Concurrently, hyperactive PI3K/AKT leads to further induction of HK2, LDHA, and glutamine-associated enzymes through mTORC1/2 and HIF1α, as well [853,857]. This dual activation mechanism elicits greater magnitude alterations in glycolytic and TCA cycle flux compared to either pathway alone [780,861]. The metabolic reprogramming rendered by oncogenic *KRAS* and PI3K cooperation is critical to sustaining drastic increases in energetic and biosynthetic requirements of CRC proliferation [862]. It also provides metabolic plasticity that enables adaptation to diverse microenvironments and evasion of cell death signals, thereby facilitating disease progression and therapy resistance [863,864]. Targeting both the MAPK and PI3K networks may help break this metabolic symbiosis, restricting the extensive metabolic transformations that fuel CRC growth and survival [865,866]. The use of combined small molecule inhibitors against MEK/ERK and AKT pathway components show promise as strategically focused combination treatments [284].

#### 4.4.4. Adaptive Metabolism in CRC: Autophagy, Catabolism, and Mitochondrial Plasticity under Nutrient Deprivation

CRC cells strongly induce autophagy as a survival mechanism under low nutrient conditions (Figure 6) [867]. During periods of glucose and glutamine depletion, which commonly occur in poorly vascularized tumor microenvironments, autophagy acts as a nutrient stress response [785]. Through lysosomal degradation of damaged organelles and proteins, autophagy recycles intracellular components into simple building blocks [868,869]. It generates free amino acids that can directly enter central carbon metabolism through replenishing TCA cycle intermediates like glutamine, or be used for gluconeogenesis to resynthesize glucose [869]. Autophagy-derived lipids and fatty acids are also catabolized to produce acetyl-CoA and ketone bodies for ATP generation in mitochondria under hypoxia [870,871]. This autophagic recycling allows CRC cells to maintain growth, proliferation and anti-apoptotic pathways even in the absence of ample exogenous nutrients [870,872]. By liberating biosynthetic precursors internally through self-digestion, autophagy acts as a key adaptive strategy that enables CRC cell survival under low glucose and glutamine conditions [871,872].

CRC tumors activate additional catabolic processes including lipolysis and protein degradation to break down stored macromolecules [873,874,875]. Enhanced lipolysis occurs through increased expression of lipases such as adipose triglyceride lipase that hydrolyze triglycerides in lipid droplets [876]. This releases free fatty acids that CRC cells can readily use through fatty acid oxidation [876]. Fatty acid catabolism in the mitochondria generates acetyl-CoA and NADH to help fuel ATP production via the electron transport chain, especially under hypoxic stress [877,878,879]. Concurrently, proteasomal degradation and autophagic recycling of proteins liberates free amino acids, especially glutamine, which acts as a critical anaplerotic substrate [880,881]. These catabolism mechanisms break down stored energy sources into smaller bioavailable nutrients that CRC cells can employ to survive periods of low glucose or glutamine availability typically found within tumors [788,799,882].

#### 4.4.5. Metabolic Diversity among CRCs: How Disease Stage, Genetics, and Location Drive Variability

CRC tumors exhibit considerable heterogeneity in their metabolic profiles based on staging, location, genetics, and other factors [883,884]. More advanced cancers stages (i.e., III–IV) demonstrate pronounced increases in aerobic glycolysis and glutaminolysis to fuel their invasive growth patterns [799]. These late-stage tumors also robustly induce autophagy and catabolic programs to salvage nutrients under nutrient stressed conditions within bulky tumor masses [885]. Additionally, the primary location of the CRC influences metabolism, as right-sided tumors commonly bearing BRAF mutations show preferential reliance on glutamine due to mitochondrial alterations induced by oncogenic BRAF signaling [70,816,886,887,888,889]. In contrast, left-sided CRCs lacking BRAF mutations typically exhibit increased dependence on glycolysis [816,843,889,890].

Intratumoral heterogeneity in CRC metabolism is also prevalent and driven by somatic mutations found in subpopulations [883,891,892,893]. Genome-wide analyses have found altered metabolic enzyme expression profiles and flux patterns between *KRAS*-mutant versus wild-type regions within CRCs [892,893,894,895]. *KRAS*-driven glycolysis and glutaminolysis renders these subclones less sensitive to metabolic therapies that may still target oxidative regions [781,785,835,896]. Additional mutations impacting genes like *HIF1A*, *IDH1*, or tumor suppressors further introduce variability in metabolic wiring between CRC tumor cells and microenvironments [897,898,899,900]. This intratumoral diversity poses challenges to targeted metabolic therapies but may be overcome through predictive biomarkers and combination treatments [901,902,903].

#### 4.4.6. Exploring the Microbiome-CRC Metabolic Interface

The gut microbiome also plays an important role in influencing CRC tumor metabolism and responses to therapy through the metabolites it produces [904]. Certain bacterial species, such as Bacteroides associated with Western diets, produce tumor-promoting effects by metabolizing primary bile acids into secondary bile acids like deoxycholic acid [905,906,907]. These secondary bile acids enter the tumor microenvironment and activate the farnesoid X receptor (FXR) in CRC cells [908,909,910]. This induces expression of FGF19, stimulating proliferative pathways such as MAPK and β-catenin, which promotes cell cycle progression and inhibits apoptosis [911]. Other gut pathogens secrete volatile organic compounds that generate oxidative stress, activating Wnt/β-catenin signaling through phosphorylation and degradation of the *APC* tumor suppressor [912,913,914]. Compounds from bacteria like *Fusobacterium nucleatum* and Enterobacteriaceae support inflammation and drive genomic instability within the CRC microenvironment [915,916,917]. Additionally, antibiotic or chemotherapy use can alter the microbiome composition and metabolite outputs through dysbiosis, potentially compromising treatment responses [918,919,920,921]. Modulation of the gut microbiome and its metabolic functions therefore presents opportunities to influence CRC metabolism and therapeutic response [907].

Commensal gut bacteria exert potent antitumor effects in CRC through the production of short chain fatty acids (SCFAs) like butyrate [907,922,923,924]. Butyrate is produced through the fermentation of dietary fiber by certain bacteria including Clostridia and *Faecalibacterium prausnitzii* [925,926,927,928]. In CRC cells, butyrate functions as a histone deacetylase (HDAC) inhibitor, preventing deacetylation of histone proteins [929]. This modifies chromatin structure, increasing transcription of tumor suppressor genes involved in cell cycle regulation such as p21 [672]. Butyrate also inhibits GSK3β phosphorylation, which stabilizes β-catenin for proteasomal degradation and blocks Wnt pathway stimulation of proliferation [930]. Through these epigenetic modifications, butyrate induces cell cycle arrest and apoptosis in CRC cells [931]. Additionally, as an HDAC inhibitor, butyrate establishes an anti-inflammatory environment in both the gut and tumor microenvironment by inhibiting HDAC activity in macrophages and dendritic cells (DCs) [932,933]. Supplementing butyrate through dietary interventions or modulating the bacterial community composition to enrich butyrate-producers represents a promising strategy to establish protective microbial metabolites that directly impact oncogenic and tumor suppressor signaling pathways critical to impairing CRC pathogenesis [934,935,936,937,938,939].

#### 4.4.7. Metabolic Rewiring during EMT and Metastatic CRC

Glycolytic reprogramming plays a pivotal role in EMT and metastasis [940]. At the molecular level, EMT is driven by alterations in key metabolic regulators, including transcription factors (TFs) Snail, Slug, and Twist that directly repress miR-200 family microRNAs, which normally target glycolytic genes *GLUT1/3* and Pyruvate kinase M2 (*PKM2*) [941,942]. Overexpression of EMT-TFs therefore induces aerobic glycolysis through transcriptional and post-transcriptional changes [941,943,944]. This includes increased HIF1α signaling via PI3K/AKT/mTORC1, leading to upregulation of glucose transporters and glycolytic enzymes phenotype [785,945,946]. CRC cells undergoing EMT exhibit increased *GLUT1/3* transcription and membrane localization to enhance glucose uptake [787,947,948]. This supports elevated glycolytic flux and lactate production under normoxia, providing nutrients and reducing equivalents to fuel the biosynthetic requirements of migration and invasion [823,949,950]. *PKM2* is also induced, shunting pyruvate away from the TCA cycle, which would otherwise contribute to energy production through oxidative phosphorylation [801]. Instead, *PKM2* promotes lactate production and supports the anabolic processes necessary for migration and invasion [679].

Mitochondrial dynamics also undergo significant alterations during EMT and metastasis of CRC cells [951]. During EMT, loss of E-cadherin triggers mitochondrial fission and fragmentation through Drp1, favoring migration and invasion [952]. At metastatic sites, tumor cells alter their metabolism to meet the bioenergetic and biosynthetic demands upon colonizing distant tissues [786,953]. Mitochondrial biogenesis and oxidative phosphorylation are often augmented, driven by oncogenes like *c-Myc* and *HIF2α* [953,954]. However, emerging evidence from metabolomic profiling reveals diverse mitochondrial heterogeneity between primary and metastatic lesions [955,956]. In fact, studies have shown that metastasized tumors within the same patient exhibit metabolic variability [949,957]. For example, some metastatic tumors rely more heavily on glycolysis or glutaminolysis depending on tissue-specific signaling cues [821,949,958]. These unique discrepancies provide avenues to develop combination therapies tailored to the vulnerabilities of each metastatic site [956]. Targeting glycolytic enzymes like GLUT or MCT inhibitors may impede EMT and cancer dissemination by disrupting metabolic reprogramming associated with these processes [855,940,959]. Similarly, blocking glutaminolysis [835] or altered oxidative phosphorylation [785] in specific metastases offers opportunities to prolong survival in advanced CRC. Continued multi-omics analyses of metabolic evolution during CRC progression should illuminate additional targets to include with conventional drugs, thereby improving outcomes for patients with liquid or solid tumor metastases [960,961].

### 4.5. Tumor-Promoting Inflammation in Colorectal Carcinogenesis

Chronic inflammation establishes a pro-tumorigenic environment in the colon for conditions such as UC and Crohn’s disease (CD) [962]. Patients with long-standing UC or CD have a 4- to 18-fold elevated risk of developing CRC compared to the general population due to prolonged gut inflammation [963,964]. Prolonged inflammation involves immune cell infiltration that secretes factors remodeling the environment into a state conducive to uncontrolled cell growth over decades [965].

#### 4.5.1. Inflammation-Driven Molecular Mechanisms

At the molecular level, macrophages and neutrophils contribute to chronic inflammation and increased cancer risk through both acute and prolonged effects (Figure 6) [966,967,968]. In the short term, these immune cells secrete ROS and cytokines to eliminate pathogens and cell debris. ROS react with DNA, potentially inducing oxidized base mutations if unchecked [969,970]. Cytokines simultaneously activate intracellular signal transduction by binding to cell surface receptors, stimulating cascades such as NF-κB that reshape the microenvironment [971,972,973]. However, decades of repeated exposure to this inflammatory barrage have deeper, long-lasting consequences [974,975]. Persistent ROS generation subjects surrounding colonic cells to chronic oxidative stress, gradually accumulating DNA damage that may mutate critical cancer driver genes [976,977,978]. Constitutive cytokine signaling also exerts epigenetic modifications and sustained activation of pro-inflammatory and pro-growth signaling like NF-κB, which disrupt normal cellular processes and set the stage for uncontrolled proliferation [979,980,981,982]. Together, these molecular alterations incited by macrophages and neutrophils establish a pro-tumorigenic microenvironment conducive to cancer development over the prolonged course of conditions such as UC and CD [966,967,968].

The NF-κB signaling pathway plays a central role in linking chronic intestinal inflammation to CRC development [983,984]. At the molecular level, pro-inflammatory cytokines like TNF-α and IL-1β stimulate NF-κB activation upon engaging their cell surface receptors (Figure 6) [985]. This triggers a cascade of intracellular events, starting with recruitment of adaptor proteins and degradation of the inhibitory protein IκB [986]. Relief from IκB repression allows the NF-κB transcription factor to translocate to the nucleus and transactivate a myriad target genes [987]. Prolonged cytokine stimulation maintains NF-κB in its active state, resulting in chronic overexpression of pro-inflammatory, anti-apoptotic, and mitogenic genes critical for tumorigenesis [668,973,988].

Sustained NF-κB signaling also exerts genomic instability through multiple mechanisms (Figure 6) [972,988,989,990,991]. It disrupts DNA damage response pathways, preventing efficient repair of cytokine-induced mutations [992,993]. NF-κB also induces expression of proteins involved in DNA replication and cell cycle progression, collectively increasing cellular mutation rates over the long-term presence of inflammation [994]. These effects directly enable accumulation of the genetic alterations required to transformed normal colonic epithelium into malignant cancer [668,989,994,995]. Therefore, persistent activation of the NF-κB pathway by cytokines represents a key molecular link between chronic intestinal inflammation and elevated CRC risk [702,972,981,996].

#### 4.5.2. Long-Term Epigenetic Repercussions of Prolonged Inflammation

Chronic inflammation also induces long-term epigenetic alterations that profoundly influence CRC risk at the molecular level [997,998]. Prolonged exposure to cytokines and ROS modifies chromatin structure and DNA methylation patterns within colonocytes over many decades [999,1000,1001]. Histone modifications induced by inflammatory signaling, such as acetylation and oxidation, relax chromatin and stimulate expression of oncogenic genes [1002,1003,1004]. Meanwhile, cytokines and ROS directly and indirectly influence DNA methyltransferases to gradually accumulate aberrant CpG island hypermethylation [1005,1006]. These heritable epigenetic changes modify cellular behaviors and phenotypes without direct DNA mutations [1007,1008,1009]. The accumulation of histone modifications and dysregulated DNA methylation across colonic epithelial cells, resulting from years of constant inflammatory cues, generate broad genomic instability conducive to cancer development [1003,1010]. Epigenetic alterations developmentally reprogram colonic tissues at the molecular level, facilitating an environment supportive of uncontrolled proliferation [1011,1012,1013]. These chronic inflammation-driven epigenetic transformations represent an additional critical link between inflammatory bowel diseases and elevated long-term CRC risk through non-mutational gene regulation changes [1014,1015,1016].

#### 4.5.3. Microbiota Interactions Exacerbate Inflammation and Carcinogenesis

**Figure 7:**
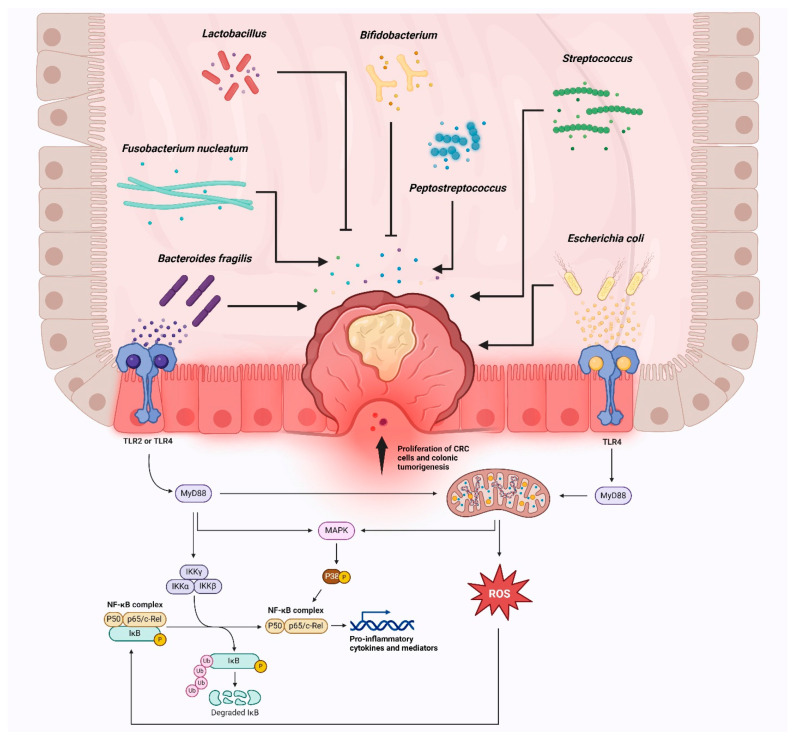
**Gut microbiome–host interactions in colorectal cancer development.** The gut microbiome plays a crucial role in influencing colorectal cancer (CRC) development and progression. This schematic image illustrates the presence of both potentially pro-carcinogenic pathogens, including *Fusobacterium nucleatum* (*F. nucleatum*), *Peptostreptococcus*, *Streptococcus*, and *Escherichia coli* (*E. coli*), as well as beneficial probiotic species like *Lactobacillus*, *Bifidobacterium*, and *Bacteroides fragilis* (*B. fragilis*). These microbes interact with the colonic epithelium, influencing the proliferation of CRC cells and the process of tumorigenesis. For instance, *B. fragilis* and *E. coli* can promote CRC progression by activating the TLR/NF-κB signaling pathway. *B. fragilis* secretes polysaccharide A (PSA), which acts as a TLR2-specific agonist. The binding of PSA to TLR2 leads to downstream NF-κB activation, a key transcription factor that promotes CRC cell proliferation, survival, angiogenesis, and metastasis. NF-κB signaling induced by PSA enhances CRC growth and development by increasing pro-inflammatory cytokines like IL-6 and IL-8, leading to chronic inflammation and fostering CRC progression. PSA also stimulates TLR2 expression on colon and CRC cells, creating a positive feedback loop wherein higher TLR2 levels induce greater NF-κB responses to repeated PSA, driving cell proliferation. PSA protects CRC cells from chemotherapy and activates NF-κB survival signaling as well. Meanwhile, *B. fragilis* lipopolysaccharide (LPS) engages TLR4, stimulating NF-κB -mediated expression of genes for survival, invasion, and angiogenesis in CRC tissues with co-expressed TLR4 and NF-κB. LPS increases cytokines like IL-1β and IL-6 via NF-κB, fueling tumor growth and metastasis. It also induces COX-2 and EMT through NF-κB. *E. coli* LPS also can bind TLR4, triggering MyD88 recruitment and mitochondrial reactive oxygen species (ROS) generation via NOX1 upregulation in a NF-κB -dependent manner. Elevated mitochondrial ROS activates MAPKs and IKB oxidation, as well as nuclear translocating NF-κB. This underscores the importance of maintaining a balanced gut microbiome to modulate the tumor microenvironment and potentially prevent or manage CRC development. Figure created using BioRender.

The gut microbiota also influences chronic intestinal inflammation and CRC risk through molecular interactions at the epithelial interface [904,1017]. Bacterial secondary bile acids generate ROS in colonocytes, directly inducing oxidative DNA damage through formation of lesions like 8-oxo-dG altered nucleotides or adducts [1018,1019]. They also bind DNA nucleotides to form bulky and etheno adducts that distort the DNA helix, resulting in mutations over years [906,1020]. As depicted in Figure 7, certain pathobionts (organisms native to the host’s microbiome) engage Toll-like receptors (TLRs) on colonocytes, recruiting adaptor proteins like MyD88 triggering downstream NF-κB signaling cascades [1021,1022,1023]. Prolonged TLR stimulation by changes in the microbiota (dysbiotic) maintains prolonged NF-κB activation leading to chronic overexpression of genes disabling DNA damage response and promoting mitogenic/anti-apoptotic pathways [980,1024,1025,1026]. This sustained dysbiosis-driven NF-κB activation exerts the same molecular effects attributed to immune cells during chronic gut inflammation that elevate risk over decades [1027,1028,1029].

In addition, the gut microbiota influences CRC development through long-term epigenetic modifications to colonocytes [1030,1031]. Microbial metabolites regulate the one-carbon metabolic pathway within epithelial cells, indirectly altering DNA methylation profiles by providing methyl groups [1032,1033,1034]. Certain metabolites like secondary bile acids have also been shown to directly influence DNA methyltransferase activity and gene methylation levels in colonocytes [1035]. Prolonged dysbiosis reshapes histone modifications over time via their effects on histone-modifying enzymes and chromatin accessibility as bacteria engage cellular receptors to stimulate epigenetic modifiers [1034,1036,1037,1038]. This results in aberrant chromatin landscapes with changes to histone codes at genes involved in processes like proliferation, DNA damage repair, and stress response [1034,1039,1040]. The accumulation of these stochastic epigenetic alterations, including dysregulated DNA methylation and aberrant histone modifications, affects gene expression profiles supporting cancer processes when accrued over decades [1041]. At the molecular level, microbes represent a critical environmental factor driving the non-mutational, heritable changes to the epigenome that developmentally reprogram colon tissues and increase CRC risk through long-term epigenetic impacts of dysbiosis [998,1036,1042,1043]. Microbial metabolites and ligands can also both directly and indirectly drive the pathogenic DNA and epigenetic alterations linking chronic gut inflammation to CRC progression [1017,1040,1044].

#### 4.5.4. Inflammation and Genomic Instability

Inflammation can also contribute to CRC pathogenesis through its impact on telomere biology [1045,1046]. Specifically, inflammatory can accelerate telomere shortening by promoting oxidative stress, DNA damage, and increased cell turnover [1047]. Chronic inflammation also leads to the activation of immune cells releasing of pro-inflammatory cytokines and ROS, all of which contribute to telomere attrition [1047,1048,1049]. Additionally, inflammatory processes can upregulate telomerase activity, which not only compensates for telomere shortening, but also contribute to the survival and proliferation of cancer cells [1050]. Furthermore, telomerase itself can have pro-inflammatory effects through the modulation of pro-inflammatory cytokine levels, which promote immune cell recruitment and an inflammatory microenvironment associated with tumor progression [1051,1052]. Overall, the interplay between telomere biology and inflammation is complex and multifaceted [1053,1054,1055]. Telomere dysfunction and chronic inflammation can mutually fuel each other, creating a vicious cycle that supports the initiation and progression of colon cancer, particularly in individuals with IBD such as UC [1045,1056]. Therefore, understanding the molecular mechanisms underlying this relationship is important for developing strategies to prevent or intervene in the development of CRC in high-risk populations [996].

In summary, chronic intestinal inflammation establishes a pro-tumorigenic environment conducive to CRC development over decades. Immune cells and gut microbiota alike perpetuate inflammation and its associated molecular changes [1057,1058]. Persistent ROS and cytokine signaling induce DNA damage, disrupt cellular processes, and activate oncogenic signaling pathways like NF-κB [1059,1060]. Prolonged inflammation also causes enduring epigenetic changes through histone modifications and aberrant DNA methylation [1061]. The cumulative molecular and epigenetic alterations derail epithelial homeostasis and increase mutational burdens in cancer genes [1013,1062,1063,1064]. Over many years, this inflammation-driven pathogenic cascade stochastically accumulate genetic and epigenetic alterations required for malignant transformation of colonocytes [1062]. Effective long-term management of chronic gut inflammation may help curb these sequential molecular events and lower CRC risk in conditions such as IBD [9,1065]. Taken together, the discussed mechanisms elucidate how chronic inflammation acts as a key driver of colorectal carcinogenesis through diverse influences at the DNA, RNA, and protein levels in addition to epigenetic changes.

### 4.6. Avoiding Immune Destruction by CRC Tumors

The immune system plays a vital role in protecting against cancer through its ability to recognize and eliminate tumor cells [1066]. The innate and adaptive immune systems work in close collaboration to identify and eliminate developing tumor cells [1067,1068]. Natural killer (NK) cells constitutively patrol tissues and induce antibody-dependent cellular cytotoxicity against malignant cells lacking appropriate self-markers [1069]. Macrophages and other myeloid cells phagocytize tumor debris and apoptotic bodies [1068,1070]. They also secrete cytokines to activate adaptive immune responses [1070]. CD8^+^ cytotoxic T lymphocytes (CTLs) have T cell receptors that directly recognize tumor-specific antigens displayed on cell surface major histocompatibility complexes (MHC) class I molecules of cancer cells [1071]. Once tumor antigens are endocytosed by resident dendritic cells (DCs) in tissues, DCs migrate to lymph nodes where they present processed peptide fragments to CD4^+^ helper T cells and CD8^+^ CTLs [1072,1073,1074,1075]. Activated tumor-specific T cells then proliferate and circulate through peripheral blood and lymph, migrating back to sites of tumor development [1076]. When this immunoediting process functions properly through immune surveillance and elimination phases, it prevents establishment of clinically evident cancer [1077]. Immunotherapies seek to re-engage the immune system’s intrinsic ability to identify and destroy malignant cells through vaccines, checkpoint inhibitors, and other modalities [1077,1078].

Unfortunately, CRC tumors evade immune detection and suppression through various mechanisms [1079,1080,1081]. One strategy is to decrease tumor antigen expression and cell surface MHC class I molecules to shield themselves from T cell recognition [1082]. Alternatively, immunosuppressive cytokines and metabolites in the tumor microenvironment also paralyze antitumor immune effector cells [1079]. Recruitment of regulatory T cells (Tregs) and myeloid-derived suppressor cells (MDSCs) establishes local immunosuppression. Tumors also exploit checkpoint molecules to inhibit T cell activation [1083,1084,1085]. By orchestrating the infiltration of suppressor cells within the tumor and surrounding stroma, CRC tumors establish an immune desert where effector T cells and natural killer (NK) cells cannot eliminate cancer cells [1083,1084,1085].

#### 4.6.1. Immune Cell Subsets in the Tumor Microenvironment

##### CD8^+^ Cytotoxic T Cells

**Figure 8:**
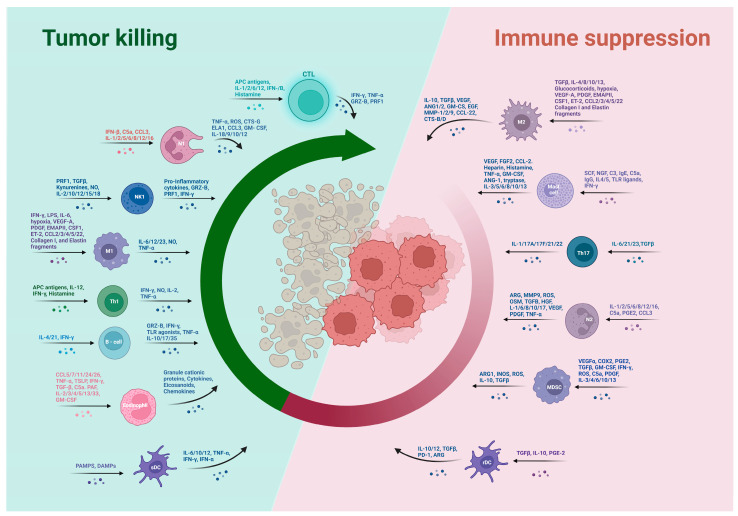
**Tumor–immune interactions and the dynamic interplay between tumor killing and immunosuppressive mechanisms.** The image presents a comprehensive overview of the complex interactions between tumor cells and the immune system, highlighting the dynamic interplay between tumor-killing mechanisms and immunosuppressive pathways within the tumor microenvironment. Key effector cells like natural killer (NK) cells and cytotoxic T lymphocytes (CTLs) can directly eliminate tumor cells through the release of cytotoxic granules, while being activated by the presentation of tumor antigens by antigen-presenting cells (*APC*s) and the presence of pro-inflammatory cytokines like IFN-γ and TNF-α, which also enhance the expression of MHC molecules and tumor antigens, making them more susceptible to immune recognition. Chemokines like CXCL9, CXCL10, and CXCL11 promote the trafficking of these effector cells into the tumor site, facilitating their antitumor functions. Conversely, regulatory T cells (Tregs) and immunosuppressive cytokines like TGF-β, IL-10, and IL-4 create an inhibitory environment that dampens the activity of effector immune cells. Myeloid-derived suppressor cells (MDSCs) inhibit T cell responses through the production of enzymes like arginase (ARG1), inducible nitric oxide synthase (iNOS), and reactive oxygen species (ROS), which deplete essential nutrients and induce oxidative stress. Other enzymes like indoleamine 2,3-dioxygenase (IDO) and tryptophan 2,3-dioxygenase (TDO) deplete the essential amino acid tryptophan, leading to metabolic stress and suppression of T cell responses. Additionally, factors like VEGF, COX2, and PGE2 not only support tumor growth but also contribute to the recruitment and function of immunosuppressive cell types. This intricate balance and crosstalk between pro-inflammatory and anti-inflammatory signals within the tumor microenvironment ultimately determines the overall efficacy of the antitumor immune response or the establishment of an immunosuppressive state that favors tumor progression, with the balance often tipped towards progression in advanced stages of cancer. Figure created using BioRender.

T cells are central players in antitumor immunity Figure 8 [1086,1087]. The CD8^+^ T cell subset recognize tumor antigens presented on MHC class I molecules and kill cancer cells [1086,1087]. However, CRC tumors suppress CD8^+^ T cell activation through several mechanisms [1085,1088]. For example, MDSCs in the tumor can secrete arginase and ROS that deprive CD8^+^ T cells of essential amino acids and induce oxidative stress [1085,1089,1090]. This renders them exhausted and unable to proliferate [1091]. CRC tumors can also recruit MDSCs and express galectins to induce T cell apoptosis [1092,1093,1094]. As a result, MDSCs present antigens in an immune subdued manner [1095] or enhance IDO/IDO2 expression [1096,1097] to deplete tryptophan essential for T cells.

Tumor cells also downregulate MHC class I to evade detection by CD8^+^ T cells [1088,1098]. In a similar fashion, cancer cells can modulate the expression of surface checkpoint molecules like programmed cell death protein 1 (PD-1) on CD8^+^ T cells to functionally impair their cytotoxic effector function within tumors [1099]. In fact, upregulation of PD-1 on CD8^+^ T cells can be induced in the microenvironment through various mechanisms including chronic antigen exposure and tumor-mediated immunosuppression [1100]. Tumors can also produce inhibitory cytokines like TGF-β [1101], IL-10 [1102], and prostaglandin E2 [1103], which directly suppress T cell effector functions. Cancer cells including CRC can also co-express the PD-1 specific ligand (PD-L1) to engage PD-1 and deliver inhibitory signals to CTLs [1104,1105,1106]. This interaction effectively dampens immune response by suppressing overall antitumor CD8^+^ T cell function [1105].

There are several promising therapeutic approaches being evaluated to block the diverse immunosuppressive pathways utilized by CRC tumors [1107]. Inhibiting checkpoint molecules like PD-1/PD-L1 using monoclonal antibodies is a direct method to reactivate exhausted T cells [1107,1108,1109]. Additionally, depleting or blocking the inhibitory effects of MDSCs [1108], Tregs [1109,1110], and immunosuppressive cytokines such as TGF-β, VEGF, IL-10, and IL-6 [1111] can relieve multiple levels of suppression. Enhancing antigen presentation through vaccination or oncolytic viruses aims to fully activate T cells [1112,1113,1114]. Targeting metabolic vulnerabilities like IDO/IDO2 depletion seeks to restore T cell proliferation [1115,1116]. Adoptive cell therapies like CAR T-cell therapy infuse large numbers of activated tumor-specific CD8^+^ T cells able to overcome immunosuppression [1117,1118]. Combination regimens involving two or more of these strategies have shown synergistic potential to reprogram the tumor microenvironment into an immunostimulatory state where endogenous T cell responses can robustly eliminate cancer cells [1119]. Proper pairing of immunotherapies is essential for effectively lifting molecular brakes across different suppression pathways controlling CD8^+^ T cell antitumor immunity in CRC [1120,1121,1122].

##### Tumor-Associated Macrophages

Macrophages play complex roles in the CRC tumor microenvironment [1123]. Two main subsets exist: M1 macrophages activated by IFN-γ and microbial signals that promote antitumor immune responses, and M2 macrophages dominant in tumors that exhibit pro-tumoral functions [1124,1125,1126]. Tumor cells secrete high levels of cytokines like IL-4, IL-10, and IL-13 that signal through STAT6 pathways and drive macrophages towards an M2 alternative activation state [1127,1128,1129]. M2 tumor-associated macrophages (TAMs) show distinctive gene expression profiles characterized by arginase-1 and Ym1/2 expression [1130,1131]. They secrete growth factors like EGF, FGF, and PDGF that stimulate proliferation of cancer cells in a paracrine manner [1125,1132,1133]. M2 macrophages also suppress CD8^+^ cytotoxic T cell functions needed to eliminate developing tumor cells [1134,1135]. Additionally, they promote processes like angiogenesis and extracellular matrix remodeling through VEGF, TGF-β, and matrix metalloproteinases (MMPs), facilitating tumor invasion and metastasis [1125,1136].

Due to these tumor-promoting functions mediated through multiple pathways, re-educating or depleting M2 TAMs is an important therapeutic strategy for eliciting antitumor immunity in CRC [1137]. Several approaches have also aimed to re-educate macrophages towards an antitumor M1 profile [1138,1139,1140,1141]. Inhibiting cytokines like IL-4, IL-10, and IL-13 that drive M2 polarization can redirect macrophages differentiation [1138]. Activating them with IFN-γ, TLR agonists and chemotherapies promotes a cytotoxic M1 phenotype capable of damaging cancers [1139]. Blocking growth factors and angiogenic factors produced by M2 macrophages also impacts tumor progression [1140]. Adoptive transfers of pre-activated M1 macrophages genetically modified to target tumors may provide a local source of tumor cell killing [1138]. Combined with checkpoint therapy, such as CTLA-4 and PD-1 blockers, programmed M1 macrophages seek to enhance macrophage-T cell crosstalk for coordinated anti-cancer immunity [1141]. As an alternative, depleting established tumor-associated M2 macrophages while simultaneously stimulating M1 polarization through targeting specific surface receptors, like PI3Kγ, holds promise [1142]. Taken together, understanding macrophage polarization dynamics will enable optimizing strategic combinations for clinically applicable measures to defeat the tumor microenvironment [1143].

##### Regulatory T Cells

Tregs play an important physiological role in controlling excessive immune response and maintaining tolerance to self-antigens [1144,1145,1146]. Characterized by expression of CD25 and the transcription factor FoxP3, Tregs comprise 5–10% of CD4^+^ T cells in healthy individuals [1147]. However, colorectal cancer cells have developed mechanisms to co-opt this regulatory network and avoid immune-mediated elimination [1148]. Considerable quantities of FoxP3^+^ Tregs are densely recruited into the tumor and surrounding stroma of CRC patients [1149,1150]. Higher densities of intratumoral Tregs directly correlate with advanced tumor stage and poor patient prognosis [1151,1152]. Within the tumor microenvironment, Tregs employ two primary suppressive strategies [1153,1154]. Firstly, through surface expression of CTLA-4, they can directly inhibit dendritic cell and macrophage involvement by outcompeting for CD80/86 binding [1153]. Secondly, Tregs secrete anti-inflammatory cytokines like IL-10 and TGF-β, which dampen the activation and effector functions of tumor-specific CD4^+^ helper and CD8^+^ killer T cells [1154]. By harnessing host Treg responses, CRC establishes local immunosuppression, which promotes unchecked growth and spread [965,1155].

There are several promising strategies being investigated to counteract the immunosuppressive influence of Tregs in the colorectal cancer microenvironment [1156,1157,1158,1159,1160,1161]. Direct depletion of Tregs offers one approach to remove their inhibitory effects on other antitumor immune cells [1156]. This can be achieved through monoclonal antibodies targeting the interleukin-2 receptor alpha (IL-2Rα) chain (CD25), which is highly expressed on Tregs [1156,1162]. Alternatively, targeting the transcription factor FoxP3 that controls Treg development and function using small interfering RNA shows potential to diminish Treg numbers [1157]. Another method is blocking the mechanisms through which Tregs exert suppression [1158,1159]. Inhibiting CTLA-4 prevents direct contact-based inhibition [1158], while targeting the indoleamine 2,3-dioxygenase pathway blocks Treg-mediated tryptophan depletion [1097]. Inflammatory cytokines like TGF-β can also be neutralized to curb immunosuppressive signaling [1159]. Additional strategies involve interfering with intracellular pathways critical for Treg stability and function [1162]. Promisingly, combining Treg-depletion or -inhibition with checkpoint therapies, vaccines or other immunomodulators demonstrates synergistic effects in unleashing robust antitumor immunity [1162,1163]. Aside from the strategies mentioned, blocking the migration of Tregs to the tumor microenvironment could also help in reducing their infiltration and immunosuppressive impact [1161]. Collectively, a multifaceted approach seeks to comprehensively relieve Treg suppression through complementary pathways [1160].

#### 4.6.2. Microbiome and Immune Response

The gut microbiome plays an important role in influencing the immune system and vice versa [1027,1164,1165,1166]. The gut is home to trillions of microorganisms that make up the microbiome [1027]. These microbes help regulate intestinal immunity and support the development of the gut-associated lymphoid tissues (GALTs) [1027]. A healthy, diverse microbiome is important for maintaining intestinal barrier integrity and priming appropriate immune responses against pathogenic microbes [1167]. Changes in the gut microbiome composition have been linked to CRC development and progression [1017,1168]. Studies have found that individuals with CRC tend to have a less diverse microbiome with reduced populations of certain beneficial bacteria like *Faecalibacterium prausnitzii* [1169]. A lower microbiome diversity or dysbiosis is associated with increased CRC risk potentially by compromising the colonization resistance against pathogenic bacteria [1044,1169]. Certain pathobionts like *Fusobacterium nucleatum* have also been found at higher levels in CRC tumors and may promote tumorigenesis [1044].

Specific probiotic strains have been shown to strengthen the intestinal barrier, a key component of gut immunity [1170,1171,1172]. *Lactobacillus rhamnosus* GG produces a soluble protein that increases the expression of tight junction proteins like zonula occludens-1 (ZO-1), reinforcing intestinal barrier integrity [1170]. A blend of probiotic strains, including *Lactobacillus acidophilus*, *Bifidobacterium bifidum*, and *Bifidobacterium lactis* also reduced epithelial cell apoptosis and enhanced barrier function in animal studies [1171]. These probiotics support mucin expression, contributing to improved barrier function and pathogen exclusion [1171]. They enhance immune surveillance of the gut through effects on dendritic cells [1172], macrophages [1173], and lymphocytes [1174]. *Lactobacillus casei* DN-114 001 promotes a Th1 immune profile by activating dendritic cells [1172]. *Bifidobacterium lactis* HN019 increases antigen presentation by maturing dendritic cells [1172,1175]. Both *Bifidobacterium longum* and *Lactobacillus acidophilus* induce NK cells cytotoxicity, which helps eliminate developing tumor cells [1172,1176].

Certain probiotic metabolites also aid gut immunity [1172,1173,1177,1178,1179]. Butyrate is a short-chain fatty acid (SCFA) produced when probiotics ferment dietary fibers in the colon [1180]. It serves as an important energy source for colonocytes and exerts potent anti-inflammatory effects [1180]. Butyrate also suppresses the activity of NF-κB and its pro-inflammatory downstream genes [1181]. It also supports the generation and function of Tregs in the colon [1182]. *Lactobacillus plantarum* has been shown to produce polysaccharide A when it colonizes the gut [1175]. Polysaccharide A activates dendritic cells by binding to the Toll-like receptor 2 [1183,1184]. This stimulates dendritic cells to secrete IL-6 and IL-23 cytokines, which drive the differentiation of protective T helper 17 (Th17) cells [1185]. It also induces Treg cell development via retinoic acid production [1186]. The balanced Th17/Treg response induced by polysaccharide A helps strengthen intestinal barrier integrity while keeping inflammation in check, both of which are crucial in halting CRC progression [1187]. SCFAs from the gut microbiota, including butyrate, propionate and acetate, impact both innate and adaptive immune responses in the colon [923,1188]. They shape the structure and composition of gut lymphoid follicles where immune cells constantly sample gut contents [923,1189]. This supports colonic immune surveillance against developing tumors [1190,1191]. The anti-inflammatory environment maintained by SCFAs also prevents excessive or prolonged inflammation, which promotes DNA damage and tumor initiation in the colorectum [1190,1191].

In summary, the immune system plays a crucial role in protecting against CRC through its ability to recognize and destroy developing tumor cells [1095]. However, like other cancers, CRC tumors have evolved sophisticated mechanisms to evade immune detection and suppression [1079,1141]. They inhibit CD8^+^ T cell activation [1192], recruit immunosuppressive myeloid cells and Tregs [1079], and alter the tumor microenvironment [1193] to establish local immunosuppression. Understanding these complex immune escape networks utilized by CRC is important for developing rational immunotherapeutic approaches [1095]. Promising strategies targeting different immune cell populations and pathways, especially in combination, show potential to lift multiple brakes on antitumor immunity imposed by cancers [1095,1194]. Modulating the gut microbiome may also influence CRC risk and progression through interactions with intestinal immunity [1195]. Harnessing a deeper understanding of tumor immunology, including the microbiome-immune interplay in the CRC microenvironment, is key to designing more effective immunotherapies that can tip the balance towards robust, coordinated antitumor immune responses capable of eradicating cancers [1196,1197,1198].

#### 4.6.3. Specialized Resistance in Cancer Stem Cells

CRC stem cells play an important role in driving tumor initiation, progression, and therapeutic resistance [1199,1200,1201,1202]. In order to attain a survival advantage, CRC stem cells overexpression ATP-binding cassette transporters like ABCB1 [1203,1204]. High ABCB1 effectively effluxes chemotherapeutic agents from CRC stem cells, limiting exposure to drugs typically used to induce apoptosis [1205]. Another mechanism involves unique metabolic processes found in CRC stem cells [946,1206]. Rather than relying heavily on mitochondrial respiration like differentiated tumor cells, stem-like CRC cells favor autophagy-dependent metabolism [1207,1208]. This makes them less susceptible to mitochondrial outer membrane permeabilization during apoptosis [1209,1210]. CRC stem cells also maintain slow cycling and quiescence, rendering them comparatively resistant to cytotoxic drugs targeting actively proliferating cells [1211,1212,1213,1214]. Enhanced DNA damage response pathways further support self-repair of lethal lesions in the stem cell genome [1215,1216,1217]. The regenerative microenvironment where CRC stem cells reside also promotes survival [70,1218,1219]. These niches secrete signals activating pro-survival pathways important for stem cell maintenance, such as Wnt and Notch, which block apoptosis when inhibited [691,1220,1221]. Immune evasion mechanisms such as loss of MHC class I molecules [1088], downregulation of death receptors [1222], and immune checkpoint pathways like PD-1/PD-L1 [1223] also hamper antigen presentation and immune killing of stem-like tumor cells. Together, these intrinsic and extrinsic adaptations reserve sufficient protection for CRC stem cell pools to escape cell death stimuli [692,1212].

The regenerative microenvironment of the intestinal crypt niche also plays a pivotal role in supporting CRC stem cell survival [1224]. Stromal cells and cytokines secreted within the crypt microenvironment activate key pro-survival pathways in stem-like CRC cells, such as Wnt/β-catenin and IL-6/STAT3 signaling [691,702,1225]. These signals serve to directly antagonize apoptosis while maintaining the stem cell phenotype [691,702,1225]. Additionally, hypoxic zones and immune evasion mechanisms in the crypt niche collectively hamper antigen presentation and shield CRC stem cells from immune-mediated killing [1226,1227,1228]. Through a combination of intrinsic adaptations in drug transportation, DNA repair, and autophagy-dependent metabolism, coupled with extrinsic support from the pro-tumorigenic stem cell microenvironment, CRC stem cell pools are uniquely equipped to withstand conventional cytotoxic therapies and fuel recurrent disease [686,1221]. Therefore, developing strategies to disrupt this regenerative niche may help target treatment-resistant CRC stem cell populations driving tumor propagation and clinical relapse [1229].

### 4.7. Sustained Proliferative Signaling and the CRC Tumor Microenvironment

The CRC tumor microenvironment consists of a diverse array of cell types that engage in bidirectional communication with cancer cells to drive tumor development and resistance to therapy [1230,1231,1232]. Cancer-associated fibroblasts are highly prevalent within the desmoplastic stroma of CRC tumors [1233]. Through secretion of extracellular matrix molecules and growth factors, like TGF-β, FGF2, and PDGF, fibroblasts restructure the physical architecture of the tissue to generate pronounced hypoxic regions of dense matrix adhesiveness [1234,1235,1236]. This pathological remodeling induced by cancer-associated fibroblasts establishes topographical niches ideally suited to harbor CRC stem-like cells [1237]. Studies show such stem cell sanctuaries enriched with fibroblast-derived signals like Wnt2 and SDF1 promote expression of genes linked to tumorigenic properties, stem cell survival, and apoptosis evasion [1219,1238]. By compartmentalizing the CRC microenvironment through aberrant deposition of new matrix, cancer-associated fibroblasts generate a pro-tumorigenic environment optimized to support the CRC stem cell pools responsible for sustaining long-term recurrence and therapeutic failure [1239,1240,1241].

In addition to cancer-associated fibroblasts, immune cells are another major cell type sculpting the CRC tumor microenvironment [1242,1243]. Specifically, TAMs extensively penetrate CRC tissue and secrete a myriad of growth factors and cytokines like epidermal growth factor (EGF), colony stimulating factor 1 (CSF-1), IL-6, TGF-β, and IL-10 [1244,1245]. Macrophages polarize to an immune-suppressive phenotype within the tumor and produce EGF [1246], CSF-1 [1247], and various inflammatory mediators [1248]. These secreted factors have been shown to amplify pro-survival and pro-inflammatory signaling cascades in neighboring CRC stem-like cells through pathways such as STAT3 and NF-κB [1125,1247,1248]. The immune-evasive polarization of TAMs towards an immune-suppressive state also helps shield CRC stem cells from immune detection and killing by T cells [1079,1249].

As CRC tumors progress, hypoxic conditions develop within the growing mass due to insufficient vascular supply [694,1250,1251]. This triggers an angiogenic switch characterized by upregulation of pro-angiogenic signals such as VEGF [1252,1253,1254]. New blood vessels then infiltrate the tumor to ameliorate hypoxic stress [1252,1253,1254]. However, the perivascular regions surrounding these tissues remain suboptimal for oxygenation [1255,1256]. Within these hypoxic perivascular niches, endothelial cells secrete high levels of stem cell-regulating factors like Wnt3A and NOTCH ligands that stimulate self-renewal pathways in nearby CRC stem-like cells through pathways such as β-catenin and Hes1 [1257,1258,1259].

In addition to stromal and endothelial cells, cancer cells themselves play an active role in sculpting their microenvironment through secretion of extracellular vesicles such as exosomes [1260,1261,1262]. Exosomes released from CRC cells transport biologically active cargo such as regulatory RNAs, proteins, and lipids that can manipulate surrounding cell populations upon fusion and cargo transfer in target cells [1263]. Specific microRNAs that are highly enriched in CRC exosomes like miR-21-5p, miR-203, miR-934, miR-25-3p, miR-130b-3p, and miR-425-5p have been shown to induce pro-tumor inflammatory phenotypes when delivered to macrophages [1264,1265,1266,1267]. This reprograms macrophages towards a pro-inflammatory M2-like state characterized through different axis like miR-21-5p promoting TLR-7/IL-6 and miR-25-3p, miR-130b-3p, and miR-425-5p targeting *PTEN*/PI3K/AKT, which in turn enhances EMT and metastasis of CRC cells [1264,1265,1266,1267].

The CRC tumor microenvironment influences cancer stem cells not only through secreted soluble factors and physical niches, but also via epigenetic reprogramming [1268,1269]. Hypoxic niches, metabolic intermediates, and intercellular signaling pathways cooperate to induce epigenetic alterations in cancer stem cells that maintain “stemness” traits enabling recurrence and therapy resistance [1219,1270,1271,1272]. For instance, prolonged HIF stabilization and chronic NF-κB/STAT activation deposit H3K4me3 enhancer marks in stem-like cells, driving self-renewal pathways through genes such as *SOX2* and *MYC* [1273,1274]. This epigenetic process drives gene expression profiles that promote tumor-initiating capacity and hinder differentiation [1273,1274]. By modifying the epigenetic landscape of cancer stem cells, the tumor microenvironment endows them with characteristics of both self-renewal and therapy resistance [1275,1276,1277,1278].

Collectively, CRC tumors coordinate a multi-pronged campaign targeting different arms of cellular suicide pathways through cell-intrinsic mutations, microenvironmental optimization of survival signals, and cancer stem cell dependencies [691,1279,1280]. This multi-tiered resistance allows CRC to thrive despite genetic instability and therapeutic insults, presenting a major challenge to treatment [691,1279,1280]. Overcoming the diverse cell death evasion strategies employed through redundancy across molecular, cellular, and tissue levels may be necessary to achieve improved clinical outcomes by more effectively eliminating tumor cells [677,720,903,1079].

#### Signaling Pathways Governing EMT

EMT is a process whereby epithelial cells adopt a mesenchymal phenotype, allowing increased migratory and invasive properties [1281]. In CRC, EMT endows cells with traits necessary for dissemination from the primary tumor [1282]. EMT is largely driven by TGF-β signaling through Smad proteins and developmental pathways such as Wnt/β-catenin [1281]. When TGF-β binds to TGF-β receptor II on cancer cells, it activates receptor I and the SMAD2/3 signaling pathway downstream [1283]. SMAD2/3 forms a transcriptional complex with SMAD4, which translocates to the nucleus [1284] to induce EMT-TFs such as Snail, Slug, and Twist [1283]. TGF-β levels are often elevated in CRC tissues and correlate with poor prognosis [1285]. Knockdown experiments have demonstrated that Snail and Slug are critical for TGF-β-mediated E-cadherin repression in CRC cells undergoing EMT [1286]. These EMT-TFs directly suppress transcription of the CDH1 gene encoding E-cadherin to disrupts adhering junctions between epithelial CRC cells, facilitating detachment [1287]. Concurrently, Wnt ligands stabilize β-catenin, inducing EMT-TFs and MMPs through association with LEF/TCF [1288]. Activation of the Wnt/β-catenin pathway promotes EMT, stemness and invasion in cancer cells [1288].

Cytoskeletal reorganization during EMT relies on Rho/ROCK (Rho-kinase) signaling [1289]. ROCK phosphorylation of myosin light chain and LIM kinases regulates actin polymerization and contractility; enabling morphological shifts during cell migration [1289,1290,1291]. In CRC models, blocking ROCK activity impairs cytoskeletal shifts, which inhibit migration seen during EMT [1290]. EMT is also characterized by increased expression of mesenchymal proteins like N-cadherin and vimentin that facilitate cell motility and interactions with the tumor microenvironment [1292,1293]. Beyond the TGF-β and Wnt/β-catenin pathways, other signaling cascades such as Hedgehog (Shh), Notch, hypoxia-inducible factors, and receptor tyrosine kinases converge on EMT transcriptional programming to fully induce the migration and invasion phenotypes crucial for CRC metastatic dissemination [1294]

### 4.8. Activating CRC Invasion and Metastasis

CSCs represent a subpopulation within colorectal tumors that possess tumor-initiating capabilities [1295]. They can self-renew and differentiate into the heterogeneous cell types that comprise the tumor [1296]. CSCs undergo EMT to acquire a migratory, drug-resistant phenotype ideal for metastatic spread [1297]. These EMT processes further favor and enrich cells exhibiting CSC biomarkers like CD44, CD133, and ALDH1, enabling direct activation of stemness and pluripotency programs [1298,1299]. Thereafter, CSCs in EMT disseminate from primary tumors due to their slow-cycling nature, drug efflux pumps, and recruitment of supportive stromal cell types to pre-metastatic sites via secreted cytokines [1300,1301].

#### 4.8.1. Adaptations for Survival and Macrometastatic Outgrowth

Upon arrival at distant organs, disseminated CRC cells must overcome challenges to survive and proliferate [1302,1303,1304]. EMT and stemness programs enhance adaptive survival pathways [1299,1305]. In fact, colonized cells re-express epithelial markers through mesenchymal–epithelial transition (MET), allowing utilization of niche signals [1299]. Other adaptations include metabolic reprogramming to utilize available nutrients via amino acid metabolism, autophagy induction, redox homeostasis, and oxidative phosphorylation [1306,1307]. The metastatic CRC secretome changes through environmental factors such as exosomes, cytokines, and growth factors that prompt angiogenesis, recruit fibroblasts, and alter the niche for favorable outgrowth [1308,1309]. Genomic evolution also occurs where metastatic cells acquire new mutations that activate pro-tumorigenic programs like Wnt/β-catenin signaling [1310]. Therefore, the plasticity of CRC phenotypes and adaptation to various environmental conditions is the gateway to metastatic colonization and expansion into macrometastases [1311].

#### 4.8.2. Preparing the Pre-Metastatic Niche through Tumor-Derived Signals

Primary CRCs prepare pre-metastatic niches in distant organs via exosomes and factors that induce chemokines, growth factors, extracellular matrix (ECM) remodeling, and inflammation [1312,1313]. Exosomal transfer of miRNAs like miR-19a, miR-29a, miR-21, and miR-200 family members are known to condition niches in the liver and lungs [1312]. Soluble factors such as TGFβ, VEGFs, and LOXL4 crosslink collagen IV to rigidify the ECM [1312]. These signals also recruit bone marrow-derived inflammatory cells like neutrophils, macrophages, platelets, and myeloid precursors that establish a supportive microenvironment [1313].

#### 4.8.3. Mechanisms of Cell Detachment, Circulation, and Extravasation

CRC cells undergoing EMT lose cell-to-cell adhesion and detach upon E-cadherin downregulation, which concomitant leads to the repression of other adhesion molecules like occludins and claudins by EMT transcription factors [1281,1314]. This transition involves the rearrangement of the cytoskeleton, leading to decreased polarity and adhesion structures in epithelial cells [1315]. Increased expression and activation of proteases like MMPs, cathepsins, and uPA degrade surrounding components of the ECM, allowing detached CRC cells to facilitate migration through surrounding tissues [1314]. In the circulatory system, EMT-associated cytoskeletal changes also grant cellular rigidity and resistance to shear stresses [1287]. CRC cells are also capable of forming microembolic clusters, which are reinforced by sustained surface expression of N-cadherin [1287]. Upon physical arrest in smaller capillary networks, selectins and integrins mediate initial tethering and rolling of circulating CRC cells followed by firm adhesion to endothelial cells expressing intracellular adhesion molecule-1 (ICAM), which bind to lymphocyte function-associated antigen-1 (LFA-1) found on tumor cells [1316,1317].

The exit of the circulatory system (or extravasation) requires the degradation of endothelial junctions like the platelet–endothelial cell adhesion molecule (PECAM) and vascular–endothelial cadherin (VE-cadherin) by MMPs, and cathepsins and uPA activation by CRC cells [1318,1319]. Meanwhile, CRC chemokines interact with endothelial ICAM and vascular cell adhesion molecules (VCAMs) to induce integrin ligands on endothelium, further stabilizing adhesion [1320,1321]. Receptors on CRC cells thereafter engage neuropilin-1 (NRP1)/focal adhesion kinases (FAK) and integrin–talin on endothelium, enabling trans-endothelium migration through junctional retraction [1322]. In tissues, niche factors including chemokine/cytokine gradients and matrix cues support extravasated CRC cell colonization [1101]. Targeting proteases, chemokines and receptor pairs involved (especially CCR6 and CCR2) could block CRC dissemination at extravasation [1101,1323,1324].

#### 4.8.4. Organotrophic Metastasis in CRC

The organ preference of CRC metastases is explained by organotropic signaling from primary tumors [809]. Molecular profiling reveals similarities between primary and hepatic lesions [1266,1325,1326]. CRC exosomes carrying specific miRNAs condition the liver niche by inducing chemokines, growth factors, and NF-κB signaling [1266,1327]. Additional tropic factors like SDF-1 chemokine and P-selectin interactions facilitate CRC microemboli homing to the liver [1266,1327]. In the lungs, CRC-derived signals similarly activate niche-specific cues through miRNAs and surfactant proteins, like SP-A and SP-D, enabling organotropic lung colonization [1328,1329,1330,1331].

### 4.9. Inducing Angiogenesis to Fuel CRC Metastasis

In order to grow at secondary sites, disseminated CRC cells must stimulate angiogenesis [1332,1333]. Hypoxic CRC cells upregulate VEGF-A through HIF1α, which activate VEGF receptors on endothelial cells to induce vessel formation [1332,1333]. Other pro-angiogenic factors like FGFs, PDGF, TGF-β, and angiogenin are also secreted [1332,1333]. Additionally, tumor-recruited neutrophils and TAMs secrete pro-inflammatory mediators that stimulate angiogenesis, which culminate into optimal angiogenic stimulation conditions to establish blood supply essential for macrometastatic outgrowth [1333].

In summary, many spatio-temporal events are required for successful CRC metastasis. TGF-β, Wnt, and other pathways induce EMT and CSC properties critical for early dissemination from primary tumors [1283,1305]. Disseminated CRC cells are then aided by pre-metastatic niche preparation and organotropic tropism to extravasate and initiate colonization [1305,1334]. Adaptive responses and angiogenesis subsequently enable growth to macrometastases [1335,1336]. Crucially, EMT, stemness maintenance, and pre-metastatic niche formation represent key opportunities for therapeutic intervention [1307,1337,1338]. Targeting these molecular drivers and pathways at different points along the metastatic cascade, from early dissemination to organ-specific colonization, holds promise for more effectively treating metastatic CRC [1307,1337,1338]. Combined anti-EMT, anti-CSC, anti-angiogenic, and niche-modulation strategies may improve patient outcomes by disrupting CRC’s ability to successfully disseminate and proliferate at secondary sites [1303,1338].

## 5. CRC Biomarkers and Therapeutic Approaches

### 5.1. Biomarkers of Immune Response

Biomarkers are biological indicators that can be objectively measured and evaluated as indicators of normal biological processes, disease progression, or therapeutic responses [1339]. Their discovery, validation, and clinical application help researchers and medical professionals better understand diseases, identify high-risk patients, guide treatment decisions, and assess new therapies [1339]. One biomarker showing promise in CRC is MMR or MSI status [1340]. Cancer cells with a defective MMR/MSI-high profile tend to have more mutations and respond better to PD-1/PD-L1 checkpoint inhibitors [1340,1341]. Testing tumors for MMR or MSI could help identify patients likely to benefit from these immunotherapies [624,1342]. Another potential CRC biomarker is PD-L1 expression on tumor or immune cells [1343]. While PD-L1 expression alone is not sufficient to predict response [1343], when used in combination with other factors it may help select CRC patients that will respond best to anti-PD-1/PD-L1 drugs [1104,1344].

Beyond genetic characteristics, the type and location of immune cells within the CRC tumor microenvironment may provide predictive information [1345,1346,1347,1348]. Several studies have found increased densities of CD3^+^ and CD8^+^ TILs within the tumor core; these cancers exhibit improved response to chemotherapy and overall prognosis [202,1349,1350,1351,1352]. Researchers believe that CRC tumors with higher levels of intratumoral CD3^+^ and CD8^+^ T cell infiltration not only enhances antitumor immune responses [1345,1346,1347,1348], but also may experience greater responses to immunotherapy due to greater pre-existing immunogenicity [624]. Ongoing research aims to define specific cut-off values of CD3^+^ or CD8^+^ tumor density that correlate best with clinical outcomes on immunotherapy [624]. In addition to cell density, the location of immune cell infiltration is significant [624,1353]. Having a predominance of lymphocytes at the invasive margins, where the tumor meets healthy tissue, rather than confined to the tumor center may impact immunotherapy efficacy [1353,1354].

Aside from CD3^+^ and CD8^+^ T cells, other immune cell populations within the tumor microenvironment show promise as predictive biomarkers [1355]. The ratio of cytotoxic to immunosuppressive cells, such as the balance between CD8^+^ T cells and FOXP3+ regulatory T cells, is being investigated [1356]. A higher cytotoxic/regulatory T cell ratio offers insights into a pre-existing antitumor immune response that may be further stimulated by immunotherapy [1357,1358]. Emerging evidence suggests this ratio, along with the densities and locations of specific immune cell subsets, can aid patient selection for checkpoint inhibitors or other immunotherapies in CRC treatment [1355,1357,1359].

### 5.2. Cancer Immunotherapy Approaches

New immunotherapy approaches aim to reverse immune suppression and boost antitumor immunity against CRC, such as checkpoint inhibitors, cancer vaccines, and adoptive cell therapies [1360,1361]. Checkpoint inhibitors aim to reverse immune suppression caused by certain immune checkpoint proteins [1362]. Pembrolizumab and nivolumab are monoclonal antibodies that target the PD-1 checkpoint receptor [1362,1363]. In clinical trials, these PD-1 inhibitors have shown responses in a subset of patients with advanced CRCs that show evidence of MMR or MSI [624,1364]. These types of CRCs tend to have more mutations in their DNA and are more visible to immune checkpoint blockade [1363]. Ongoing research aims to identify additional biomarkers that can help predict which CRC patients are most likely to benefit from PD-1 inhibitors [1363]. Combining PD-1 inhibitors with other immunotherapies, like vaccines, is also a promising avenue being explored in clinical trials [1364].

Ipilimumab works through a different mechanism by targeting the CTLA-4 checkpoint receptor [1365]. CTLA-4 acts earlier in the immune response than PD-1 [1365], and ipilimumab appears to activate more T cells in the antitumor response [1366,1367]. However, this activation comes at the cost of more immune-related side effects [1368]. In pre-treatment, ipilimumab has shown limited activity as monotherapy for CRC [1368]. To potentially improve responses, ipilimumab is now being studied in combination with PD-1 inhibitors, chemotherapy, radiation therapy, or other immunotherapies in ongoing clinical trials [1368,1369]. The goal is to make CRC tumors more visible while also enhancing T cell function through dual checkpoint blockade [1366,1370].

Cancer vaccines, on the other hand, attempt to boost the body’s natural antitumor immune response [1371]. These vaccines can contain tumor-associated antigens that train the immune system to recognize and attack the cancer [1371]. One of the most studied tumor antigens for CRC vaccines is carcinoembryonic antigen (CEA) [1371]. CEA is highly expressed in many colorectal tumors but is also present at low levels in some healthy tissues [1371]. Vaccines containing CEA aim to induce immune responses against this antigen, training T cells and antibodies to recognize and destroy CEA-expressing cancer cells [24,1371,1372]. Several CEA vaccine candidates have shown promising results in clinical trials, generating CEA-specific immune responses in CRC patients [1371,1372,1373,1374,1375]. Ongoing research is optimizing the dosing and combinations of CEA vaccines to improve their clinical efficacy [1371].

Mutated *KRAS* is another antigen being targeted with experimental CRC vaccines [1376]. *KRAS* mutations are very common in CRC (45% of cases) and help drive tumor growth [1377]. Vaccines containing peptides from the most prevalent *KRAS* mutations including G12C, G12D, G12V, and G13D, have induced T cell responses against mutated *KRAS* in early-phase trials [1376]. Combining *KRAS* or CEA vaccines with checkpoint inhibitors may further boost these immune responses [1376]. Researchers are also exploring mRNA and viral vector-based vaccines to enhance the delivery and immunogenicity of tumor-associated antigens like *KRAS*, CEA, and others [1378,1379,1380]. By improving antigen presentation and induction of T cell and antibody immunity, these newer generation cancer vaccines aim to provide greater clinical benefit than past generations [1376].

Adoptive cell therapies such as chimeric antigen receptor (CAR) T cell therapy take this approach a step further by extracting a patient’s own immune cells, such as T cells, engineering them to express artificial tumor-targeting receptors in the lab, and then infusing the enhanced immune cells back into the patient to target the cancer [1381]. In early trials for CRC, CAR-T cells have been designed to target the CEA antigen expressed by many colorectal tumors [1382]. After being activated and multiplied in the lab, the anti-CEA CAR-T cells have shown some success in eliminating CEA-positive cancer cells [1382]. Researchers continue optimizing CAR design and working to overcome obstacles like T cell exhaustion [1383].

Another approach involves isolating and activating TILs found naturally within colorectal tumors [1384]. These TILs have already demonstrated an ability to infiltrate and attack the patient’s own cancer cells [1384]. In the laboratory, TILs are selected, grown in large numbers, and re-infused back into the patient together with therapies to support their expansion [1384,1385]. Early results show TIL therapy can generate antitumor responses and patient benefits [1385,1386,1387]. Ongoing work focuses on integrating TILs with checkpoint blockade to further potentiate their long-term anti-cancer activity [1387]. Adoptive cell therapy offers a personalized avenue for CRC treatment but will require more clinical evaluation [1386].

### 5.3. Therapeutic Strategies to Restore Cell Death

#### 5.3.1. Targeting the Intrinsic Apoptotic Pathway

The intrinsic pathway acts as a convergence point for numerous cell death signals such as DNA damage, oxidative stress, and hypoxia [1388]. Drugs that modulate this central hub have the potential to synergize with diverse anticancer therapies including chemotherapy, radiotherapy, and molecularly targeted agents [1388,1389]. Reactivating the intrinsic pathway allows these therapies to more effectively eliminate CRC cells through mitochondria-mediated apoptosis [710,1388]. By contrast, upstream extrinsic signals are more easily disrupted by cancer processes [683]. Therefore, reinstating the intrinsic pathway provides a robust downstream amplified cell death response [710,1388].

#### 5.3.2. Reengaging the Mitochondrial Death Machinery

One approach is re-activating the intrinsic mitochondrial apoptosis pathway in CRC cells using targeted agonistic agents [687,732]. The intrinsic pathway is normally activated in response to cellular stress like DNA damage, which triggers pro-apoptotic Bcl-2 family proteins like Bax and Bak to permeabilize the outer mitochondrial membrane [1390]. This allows release of pro-apoptotic factors from the mitochondria that initiate caspase activation and cell death [1390]. Chemotherapeutics such as 5-FU can induce this pathway through DNA damage [687]. However, anti-apoptotic Bcl-2 proteins like Bcl-2, Bcl-xL, and Mcl-1 are often overexpressed in CRC and inhibit the mitochondrial death response [1391,1392,1393].

Drugs aimed at disabling anti-apoptotic Bcl-2 proteins therefore seek to lift their blockade of the intrinsic pathway [1394]. BH3 mimetic drugs bind these proteins and disrupt their interaction with Bax/Bak [705,1395]. Venetoclax is an FDA-approved oral Bcl-2 inhibitor showing promise in restoring mitochondrial apoptosis in chronic lymphocytic leukemia (CLL) cells [1396]. Similarly, Navitoclax targets both Bcl-2 and Bcl-xL, providing broader inhibition [1396,1397,1398,1399]. Accordingly, combining these Bcl-2 inhibitors with chemotherapy allows synergistic reactivation of the intrinsic pathway through simultaneous DNA damage and anti-apoptotic blockade [1400]. Therefore, sequential or concurrent use of Bcl-2/Bcl-xL inhibitors with DNA damaging agents represents an attractive strategy for overcoming redundancy between survival proteins and inducing mitochondrial outer membrane permeabilization in apoptosis-resistant CRC [1400,1401,1402].

#### 5.3.3. Reinstating the *TP53* Guardian of Apoptosis

Mutations in the *TP53* tumor suppressor gene occur in over 50% of CRC cases and severely hamper the intrinsic apoptotic response [453]. As a master transcriptional regulator, wild-type *TP53* activates pro-apoptotic Bcl-2 family members like PUMA and Bax upon sensing DNA damage from chemotherapy or radiation therapy [423]. However, mutant *TP53* fails to induce these cell death effectors even in the face of severe stresses [423]. To this point, small molecules like PRIMA-1 and APR-246 are being investigated that can bind to the mutated *TP53* core domain, correct misfolding and restore wild-type conformation and function [1403]. PRIMA-1 and APR-246′s correction of misfolding suggests potential for therapeutic applications in CRC by reactivating apoptotic responses associated with *TP53* mutations [1404].

Reactivated mutant *TP53* can then rescue mitochondrial apoptosis through downstream targets in a similar manner to wild-type *TP53* [453]. Preclinical research shows PRIMA-1 and similar molecules strongly sensitize *TP53*-mutant CRC models to DNA damaging agents by re-establishing *TP53* tumor suppressor signaling [1405]. Ongoing clinical trials are evaluating *TP53* reactivating drugs alone and with chemotherapy in patients selected by tumor sequencing validation of *TP53* status [1406]. Early results indicate induction of cell death pathways in mutant *TP53* cancers [1406]. Therefore, targeting both *TP53* and anti-apoptotic Bcl-2 proteins represents an attractive combination approach, as each modulates independent but intersecting components of intrinsic apoptosis regulation [519,1407].

#### 5.3.4. Bypassing IAP-Mediated Apoptotic Resistance

The intrinsic pathway converges on the activation of effector caspase proteases to execute apoptosis [1390,1408]. However, inhibitor of apoptosis proteins (IAPs) can directly bind and inhibit caspase-3, -7, and -9, halting the apoptotic cascade [1409,1410]. IAPs like cIAP1, cIAP2, and XIAP are often overexpressed in CRC and prevent cell death signaling downstream of mitochondria [687,1411]. A therapeutic strategy uses small molecule Second Mitochondrial-derived Activator of Caspases (SMAC) mimetics that antagonize IAP proteins by mimicking the endogenous IAP-antagonist SMAC [1411,1412]. By displacing IAPs from caspases, SMAC mimetics liberate the apoptosis execution machinery and amplify death signaling initiated by other pathway modulators [1411].

Preclinical studies show SMAC mimetics, such as birinapant, potentiate the effects of DNA damaging chemotherapy and radiotherapy in CRC cell lines and models [1413,1414]. These mimetics induce RIPK1-dependent necroptosis, contributing to their efficacy in CRC models [1413,1414,1415]. Combination regimens demonstrate strong synergistic interactions to induce tumor cell apoptosis through parallel mechanisms of cytotoxic stress imposition and inhibition of caspase blockades [1413,1414,1416]. Ongoing clinical trials are evaluating SMAC mimetics alone or combined with genotoxic agents, showing signs of pathway modulation and preliminary efficacy in selected solid tumors including CRC [1417,1418].

#### 5.3.5. Targeting the Extrinsic Apoptotic Pathway

The extrinsic pathway offers an alternative route to trigger apoptosis in CRC cells when the intrinsic pathway is disrupted [687,723]. It is activated by death receptor (DR4/5) ligand binding, principally FasL and TRAIL, which recruits caspase-8 to initiate caspase cascades [687,723]. However, CRC tumors frequently downregulate or mutate DR4/5 receptors and upregulate decoy receptors to negate extrinsic signals [1419,1420]. Targeted therapies aim to overcome these blocks by supplementing death ligand activity through TRAIL receptor agonists or blocking inhibitory decoy receptors [1421,1422,1423,1424]. Initial clinical evidence suggests some TRAIL monotherapy efficacy in selected CRC, demonstrating feasibility of modulating extrinsic signaling [731,1425].

Combining extrinsic modulators with drugs targeting the intrinsic pathway holds promise for potent synergy through concurrent activation of proximal caspase-8 and distal effector caspase-3/7 [1426,1427]. Preclinical models demonstrate robust apoptosis when TRAIL agonism is combined with inhibitors of anti-apoptotic Bcl-2 proteins [1426]. This dual approach leverages both major induction routes to trigger robust, redundant caspase cascades even in apoptosis-resistant CRC [1427]. Rational sequencing of extrinsic pathway modulators with cytotoxic chemotherapy also shows potential to sensitize CRC to DNA damage [1427].

In addition to DR4/5 downregulation, overexpression of c-FLIP is another common resistance mechanism deployed by CRC cells to inhibit death receptor signaling [1428]. Precisely how c-FLIP inhibits caspase-8 activation involves competition for recruitment to the DISC complex that forms upon DR4/5 ligation [1429,1430]. c-FLIP structurally resembles caspase-8 but lacks protease activity, essentially acting as an inhibitory stowaway that prevents caspase-8 dimerization and autoproteolysis required for its apoptotic function [1431]. Small molecule c-FLIP inhibitors in development disrupt this protein–protein interaction to free caspase-8 [1432]. Studies show they strongly potentiate DR4/5 agonist-induced apoptosis in CRC models when combined [1428,1433]. The dual blockade comprehensively dismantles extrinsic adaptive resistance, with c-FLIP inhibition restoring receptor-initiated caspase-8 activity while DR4/5 agonism provides tonic pro-death signaling [1428]. This synergistic bimodal approach to re-engage both the receptor and downstream extrinsic machinery holds promise as a rationally designed strategy deserving of clinical evaluation in biomarker-selected CRC patient groups [1434,1435].

Adoptive T cell therapies engineered to present TRAIL on their surface provide an alternative extrinsic modulatory approach [1436]. Upon CAR-mediated homing to tumor sites, these “TRAIL-CAR” T cells induce localized DR4/5 engagement and extrinsic apoptosis [1437,1438,1439]. Early studies demonstrate the feasibility of generating TRAIL-CAR T cells that persistently kill CRC cell models through apoptosis [1440]. However, key challenges remain in fully harnessing the potential of this approach as prolonging TRAIL expression and maintaining robust CAR T cell engraftment over time are critical for durable antitumor effects [1441]. The tumor microenvironment can also limit T cell function through immunosuppressive mechanisms [1442]. Developing strategies to protect CAR T cells through costimulatory domains or adjuvant therapies may help overcome these inhibitory factors [1441,1443]. Combining TRAIL-CAR T cells with targeted agents reactivating intrinsic apoptosis could further synergize killing through non-redundant mechanisms [1441].

With continued engineering refinements and insights from ongoing clinical research, TRAIL-CAR T cell therapy holds promise as a personalized precision medicine for CRC subsets [1444,1445]. Integrating predictive biomarkers may help discern optimal patient subsets for specific extrinsic modality trials such as evaluable for c-FLIP dependence or immunosuppressive tumors amenable to CAR T cell therapies [1444,1446]. Combined predictive and dynamic pharmacodynamic monitoring moreover allows rational sequencing of extrinsic targeted drugs or immunotherapies with chemotherapy to maximize apoptotic responses in resistant CRC [1444,1446].

#### 5.3.6. Inducing Non-Apoptotic Cell Death

When apoptosis resistance develops in CRC, targeting alternative programmed cell death modes holds value [1447]. Necroptosis is a lytic, inflammatory form of regulated necrosis induced upon TNF receptor activation [1447,1448]. CRC cells often overexpress necroptosis suppressors like RIPK1 to avoid this fate [1449]. RIPK1 plays a crucial role as a scaffold protein in the necroptosis signaling cascade [1450]. High RIPK1 expression in CRC cells suppresses necroptosis by interfering with this RIPK3 activation step [1449]. Small molecule RIPK1 inhibitors currently in preclinical development selectively block RIPK1′s necroptosis inhibitory function [1451,1452]. Without RIPK1′s brake, kinase activity shifts to RIPK3 phosphorylation upon death receptor stimulation or genotoxic stress [1451]. This drives formation of the necrosome complex and downstream MLKL phosphorylation to perforate membranes in necroptotic cell death [1451]. Studies show RIPK1 inhibitors effectively sensitize CRC models otherwise resistant to TNF-driven necroptosis through this targeted release of the necroptotic brake [1453].

Necroptosis also elicits antitumor immunity more strongly than apoptosis [744]. When cells undergo necroptosis, their membranes rupture in a lytic process that release damage-associated molecular patterns (DAMPs) like HMGB1 and ATP [744]. These act as danger signals to stimulate nearby dendritic cells and recruit immune effectors like NK cells [744,1454]. Activated dendritic cells can then migrate to lymph nodes and prime T cell responses against tumor antigens [1455]. The inflammatory contents released from necroptotic cells also promote inflammation within the tumor microenvironment [1456]. This makes dying tumor cells more visible to adaptive immune cells and supports the development of long-term immunological memory [1456]. As such, inducing necroptosis represents an attractive strategy to not only directly kill CRC cells, but also stimulate protective antitumor immunity less obtainable through the non-immunogenic process of apoptosis [1448].

Autophagy likewise assumes both tumor suppressive and promoting roles in CRC depending on context [867]. Autophagy is a catabolic process involving lysosomal degradation of cellular components that cancer cells can subvert for pro-survival purposes under stressful conditions like nutrient deprivation or chemotherapy [867]. However, excessive autophagy can also trigger non-apoptotic programmed cell death termed autophagic cell death [753]. The context-dependent roles of autophagy in CRC make both inducing and inhibiting autophagy potential therapeutic strategies [753,867]. In the early stages of CRC, autophagy can reduce genetic instability and promote an anti-cancer immune response, but in established tumors, it can confer resistance to metabolic stress and therapy [1457]. Drugs that force high levels of autophagy beyond a tolerable threshold may induce autophagic cell death [1458]. Conversely, autophagy inhibitors would block its pro-survival functions during chemotherapy or prevent tumor initiation in predisposed individuals [1459].

Preclinical models provide promising validation of autophagy modulation in CRC [1460,1461,1462]. Inducers like Rapamycin demonstrate autophagic cell death capabilities when combined with standard therapeutics, while inhibitors like hydroxychloroquine (HCQ) enhance chemosensitivity [1463]. However, the challenge of developing autophagy inhibition as a therapeutic strategy lies in the potential for contradictory or inconsistent results, as autophagy can have both cytoprotective and nonprotective functions [1464]. So far, the FDA-approved autophagy inhibitors chloroquine (CQ) and HCQ are currently being evaluated in clinical trials for their safety and efficacy in cancer therapy [1465]. For example, a phase II trial combined the autophagy inhibitor HCQ with FOLFOX chemotherapy and bevacizumab and observed increases in the autophagy marker LC3 with a complete response rate of 11% in patients with metastatic CRC [1466]. Additionally, phase I trials found combinations of HCQ with temozolomide [1467] or the mTOR inhibitor temsirolimus [1468] to be safe and show beneficial antitumor activity in solid tumors including CRC. However, another phase I trial reported HCQ treatment with the AKT inhibitor MK-2206 was tolerable but had minimal antitumor effect in CRC [1469]. Furthermore, in vitro studies showed autophagy inhibition by chloroquine (CQ) enhanced the anti-proliferative effects of 5-FU chemotherapy [1470] and bevacizumab [1471] in CRC cell lines. On the other hand, a phase I trial found no significant clinical improvement when combining HCQ with the HDAC inhibitor vorinostat in renal and CRC [1472]. Ongoing studies are evaluating optimal dosing schedules and sequencing of these multi-drug regimens [1465]. Selection of CRC subtypes most reliant on autophagy for survival is also an important focus, with biomarkers like LC3 expression being studied [753,1473]. Managing adverse events particular to CRC patients remains essential [867]. Given autophagy influences CRC development at different stages, patient characteristics like tumor mutation burden (TMB) are also being investigated to define contexts where autophagy inhibition may be most beneficial [70,1474,1475]. Large biomarker analyses continue enrolling CRC patients to further refine candidate biomarkers for clinical application [1460]. Ultimately, these ongoing combination trials aim to establish effective and safe approaches for leveraging autophagy modulation to improve CRC treatment outcomes [1460,1474].

Microtubule-targeting agents (MTAs) such as taxanes are a mainstay of CRC chemotherapy [1476,1477,1478]. These drugs work by interfering with the formation of the mitotic spindle during cell division [1476,1477]. This induces a type of non-apoptotic programmed cell death called mitotic catastrophe [1476,1477]. By trapping cells in abnormal mitosis, MTAs cause extensive DNA damage that cells cannot repair, leading to mutations, multinucleation, and senescence [1479,1480]. Several studies have investigated the use of antimitotic drugs, which interfere with cell division, as potential treatments for CRC and colon cancer. Antimitotic drugs such as taxanes and vinca-alkaloids like vinflunine that target microtubules have shown activity against CRC cell lines in preclinical research [1481,1482]. Accordingly, the antitumoral effect of these two MTAs have been reported in CRC clinical trials [1483,1484]. While this halts cancer cell proliferation, it may not outright kill apoptosis-resistant tumors [1478]. However, combining these agents with modulators of apoptosis pathways such as Bcl-2 inhibitors could tip cells over the edge into true cell death via mitotic catastrophe [1478,1485]. By augmenting the DNA damage and disrupted cell cycle induced by MTAs, resistant CRC cells may be effectively eliminated through mitosis gone awry rather than traditional programmed cell death pathways [1486]. In this regard, several MTAs have been tested or used in combination with other chemotherapy agents for better treatment outcomes. For instance, paclitaxel and docetaxel, the first generation taxanes, are often combined with platinum drugs like cisplatin for cancers like breast and lung [1487]. A newer taxane, larotaxel, showed promise in phase I/II trials when combined with cisplatin for non-small cell lung cancer, metastatic breast, and bladder cancer [1488,1489,1490]. The antibody-drug conjugate brentuximab vedotin, which links an anti-CD30 monoclonal antibody to the microtubule-disrupting agent monomethyl auristatin E, demonstrated efficacy against Hodgkin’s lymphoma when combined with cytotoxic chemotherapy in clinical studies [1491]. Fosbretabulin, a vascular disrupting agent from the combretastatin family that targets the colchicine binding site, has been evaluated pre-clinically in combination regimens for lung cancer and thyroid carcinoma [1492,1493]. Collectively, it seems that this strategy has the potency to eliminate even apoptosis-resistant CRC tumors by combining the mitotic catastrophe elicited by microtubule disruption with the blockade of anti-apoptotic molecules [1476,1477,1478].

### 5.4. Therapeutic Challenges and Emerging Approaches

#### 5.4.1. Tumor Plasticity and Adaptive Resistance in CRC

One of the major challenges is the remarkable plasticity and adaptive abilities of CRC tumors [1494]. The plasticity of CRC poses a significant hurdle because it allows tumors to dynamically reprogram their signaling networks in response to therapies [1494,1495]. Even when prominent pro-survival pathways are simultaneously inhibited, cancer cells retain the capacity to engineer diverse backups that maintain proliferative and anti-apoptotic drives [1494]. They can alter expression of downstream components, modify crosstalk between nodal points, or activate entirely different collateral routines [1496]. This remarkable adaptability inflates the number of combinations needing investigation to comprehensively disable fallback options [1495,1496].

Compounding this challenge is the ability of CRC to enact subtle compensatory shifts that evade detection [1497,1498]. Current profiling resolution may miss low level changes to signaling routing or alternative cascades recruited deep within signaling webs [1497,1499]. This enables adaptive survival transformations to initiate below profiling thresholds until clinical resistance emerges [1340,1497,1499]. Developing higher sensitivity techniques and more sophisticated analytics approaches is needed to track subtle rewiring over time [1498].

An additional layer of complexity arises from intratumoral heterogeneity [901,1500]. Different subclones within a tumor possess divergent genomic and epigenomic alterations [1500]. This implies individual subpopulations could employ distinct escape routes from combination therapies [1501,1502]. Treatments must eliminate resilient subclones without enriching rare resistant variants, requiring personalized strategies tuned to the ever-evolving makeup of each patient’s unique cancer [1503]. Addressing such multifaceted plasticity demands innovative investigative avenues [1503,1504].

The extensive crosstalk between tumor cells and the microenvironment allows survival signaling to spread beyond cell-autonomous control [1505]. When inhibitors deactivate survival circuits inside cancer cells, paracrine factors secreted by surrounding stromal and immune cells can restore this lost functionality [1506]. Fibroblasts in particular play a key role, releasing a diversity of cytokines, chemokines, and growth factors that revive dormant proliferative pathways in tumor cells [1507]. Some of the primary factors involved include IGF, IL-6, HGF, and FGF, which can reactivate downstream PI3K/AKT and Ras/Raf/MEK/ERK cascades even when directly targeted. This restores anti-apoptotic functions and circumvents cell-intrinsic pathway blockade [1508].

The stroma also participates in modifying the extracellular matrix to promote plasticity [1509,1510,1511]. Fibroblasts laying down new ECM components and remodeling fibrillar architecture provide an infrastructure for proliferation cues [1509]. They interact through integrins and other adhesion receptors to reestablish signal transduction in anchorage-dependent cancer cells sheltered within favorable niches [1510]. The dynamic interplay between tumor cells, fibroblasts, immune cells, and the ECM they construct greatly enhances phenotypic variability [1511].

#### 5.4.2. Combination Approaches to Overcome Redundancy-Driven Resistance in CRC

As discussed, CRC cells have developed numerous mechanisms to evade apoptosis and survive [1512,1513]. Single targeted therapies often fail because when one pro-survival pathway is inhibited, backup survival pathways often compensate to maintain viability [1512,1513]. There is significant crosstalk and redundancy between key proliferation and survival signaling pathways in CRC like Wnt/b-catenin, EGFR/MAPK, and PI3K/AKT [683]. When faced with cytotoxic drugs or molecularly targeted agents, CRC cells can alter signaling through these pathways to continue propagating anti-apoptotic signals [720,1512]. This redundancy has made inducing cell death through single agents a significant challenge in CRC treatment [720,1512].

However, combination therapies may be able to overcome these survival adaptations by simultaneously blocking multiple pro-survival nodes [1514,1515]. One approach is to combine agents targeting the EGFR pathway with inhibitors of downstream signaling molecules [1514,1515,1516]. Cetuximab or panitumumab combined with MAPK pathway inhibitors like vemurafenib, which targets B-Raf, aim to simultaneously block both upstream receptor activation and downstream signal propagation, which leaves no avenues for survival signals to continue [1516]. Ongoing studies are exploring EGFR inhibition paired with PI3K/AKT/mTOR blockade as another option, taking advantage of crosstalk between these proliferation pathways [849]. For example, the combination of EGFR and mTOR inhibitors (erlotinib and RAD001, respectively) has modulated the growth and autophagy level in SCLC cells [1517]. Similarly, the dual inhibition of PI3K and mTOR has been effective in reducing renal cell carcinoma (RCC) proliferation and viability [1517]. In CRC, the combination of a PI3K/mTOR inhibitor (PF-04691502/PF-502) and a MEK blocker (PD-0325901/PD-901) has demonstrated enhanced anti-proliferative effects [1518]. Furthermore, the targeting of both EGFR and mTOR by a combination of erlotinib and temsirolimus, respectively, has been effective in EGFR-resistant squamous cell carcinoma (SCC) [1519]. These findings suggest that the dual inhibition of EGFR and PI3K/AKT/mTOR pathways may be a promising approach in solid tumors including CRC [849].

Immunotherapies are also being evaluated both alone and in combination with other treatment strategies for their potential to enhance CRC outcomes [624]. One proposed mechanism is that the use of chemotherapy and targeted therapies can make tumor cells more recognizable to the immune system by damaging and killing tumor cells, which increases the release of antigens from these dying cells [1520]. This antigen release then makes the tumor cells a more visible target for immunotherapy agents to elicit an antitumor immune response [1520]. In this accordance, studies are exploring the optimal combinations and sequencing of immunotherapies, chemotherapy, and targeted therapies to take advantage of these synergistic immune-mediated effects against CRC (Table 3). Early studies on combinations of Cetuximab with anti PD-1 Avelumab [1520,1521] or the combination of panitumumab with (anti PD-1 inhibitor) and ipilimumab (anti CTLA-4) in CRC have shown promising response rates [1522]. Combining oncolytic viruses with checkpoint therapy also aims to generate a similar one-two punch in which engineered viruses selectively infect and lyse tumor cells while stimulating inflammatory signals to recruit immune cells [1523], and immune checkpoint blockade ensures those attracted cells can fully eliminate any remaining cancer cells [1524]. In this regard, the phase I/II clinical trial NCT01413295 was performed to evaluate the efficacy of combination of avelumab (anti-PD-L1) plus autologous dendritic cell (ADC) vaccine in pre-treated MSS metastatic CRC patients [1524]. The investigators reported that using the autologous dendritic cells pulsed with autologous tumor antigens as a third-line therapy was found to be safe and well tolerated, but exhibited only modest clinical activity in target patients [1524]. Table 3 provides more details of the clinical trials discussed in this section.

Adoptive T-cell therapies are also undergoing investigation when combined with other modalities [1579]. CAR T-cells engineered to target CRC-associated antigens show potential but can be limited by the immunosuppressive tumor microenvironment [1580]. Combining CAR T-cells with targeted agents may help overcome this by inhibiting pro-survival signaling and making the environment less hospitable to tumor cells [1579,1581]. Sequencing treatments with checkpoint therapy following T-cell infusion may also help the adoptively transferred cells persist longer and mount a more effective antitumor response [1582]. Overall, diverse combination regimens bringing together immunotherapies, targeted drugs, and conventional therapies hold promise for overcoming resistance to single treatments in CRC [1583].

Newer targeted agents are expanding the toolbox for combining treatments. MDM2 inhibitors activate the *TP53* pathway to induce apoptosis, and combining these drugs with cytotoxic chemotherapy aims to trigger a one-two punch against cancer cells [1584,1585]. Bcl-2 family inhibitors also synergize with standard therapies by disabling a major anti-apoptotic mechanism [1586,1587,1588]. These novel targeted drugs may help bypass resistance when paired with established approaches [1589]. Future combinations could also incorporate immunotherapy and epigenetic modulators that downregulate pro-survival genes [1402]. By simultaneously hitting cancer cells through multiple death pathways, like proteolysis-targeting chimeras (PROTAC) technology, these diverse combination regimens hope to achieve what single agents cannot [1590].

Sequence and schedule also impact the success of combinations [1591,1592]. Giving agents together continuously may antagonize their effects through offsetting mechanisms of action or toxicity [1591]. Alternating treatment periods or intermittent dosing using metronomic schedules helps reduce antagonism while still attacking the tumor through different vulnerabilities [1591]. This sequential approach also applies continuous selective pressure over time to less resistant subclones [1592]. Properly spacing out components addresses tumor heterogeneity by targeting diverse subpopulations sequentially as they evolve resistance [1593]. Ongoing clinical investigations are defining optimal sequencing and schedules to maximize benefit of priority combination partners for CRC patients [1594]. Through rigorous evaluation of new combinations and administration schedules, researchers aim to establish readily implementable multi-drug regimens as standard of care [1594]. This strategy acknowledges the evolving nature of tumors and aims for effective adaptation [1595].

### 5.5. Necessity of Biomarker Development for Precision Care

The development of predictive biomarkers is critical as clinicians now have more treatment options to combine targeted drugs, immunotherapies, and chemotherapy [1596,1597,1598,1599]. Without biomarkers to guide patient selection, combination regimens risk being administered indiscriminately without accounting for inherent tumor vulnerabilities [1596,1597]. This could expose some patients to toxic side effects without meaningful benefit [1598,1599,1600]. Biomarkers that can identify subgroups most sensitive or resistant to specific multi-drug partnerships will optimize clinical outcomes and healthcare costs by ensuring only suitable patients receive personalized therapies [1600].

In CRC, molecular profiling technologies have already identified several promising predictive biomarkers to guide combinatorial regimens [1600]. RAS and BRAF mutations are among the most well-validated predictive biomarkers used in the clinic today to personalize CRC treatment [1600]. Around 40–50% of mCRC tumors harbor mutations in *KRAS* or NRAS which have been consistently associated with primary resistance to anti-EGFR monoclonal antibody therapies like cetuximab and panitumumab [1601,1602]. Genomic profiling tests for these predictive alterations are now the standard of care to select appropriate patient populations for anti-EGFR targeted agents combined with chemotherapy [1601]. However, a significant percentage of RAS/BRAF wild-type tumors also fail to respond to anti-EGFR therapies [1603]. This highlights the potential for additional predictive factors to be identified [1603]. Emerging multi-omics approaches integrating genomic, transcriptomic, and proteomic data promise to uncover new signatures beyond single genes [1604]. For example, dysregulation of downstream or parallel pathways due to epigenetic changes or protein phosphorylation may also drive anti-EGFR resistance [1601,1605,1606].

Liquid biopsies, such as cell-free DNA (cfDNA) analysis, offer several advantages for CRC management over traditional tumor tissue profiling [1607,1608]. They provide a less invasive method to serially monitor tumor genetics throughout treatment [1607]. This could help address tumor heterogeneity [1609] and track acquired resistance signatures that emerge [1609], which tissue biopsies may miss. Several studies have demonstrated high concordance between RAS/BRAF mutations detected in plasma ctDNA samples and tumor tissue in mCRC patients [1610,1611,1612,1613]. These findings suggest that plasma-based testing can be a viable alternative to tissue-based testing for determining RAS/BRAF status in mCRC patients, with potential implications for treatment selection and monitoring [1610,1611,1612,1613]. By tracking alterations in resistance genes like EGFR or new drivers of metastases like *KRAS* G12D, researchers hope to guide sequencing of targeted agents, immunotherapies or chemotherapy [1596,1614,1615]. In this accordance, ongoing research aims to expand liquid biopsy applications [1616,1617,1618,1619]. Large prospective clinical trials are investigating whether serial ctDNA monitoring can predict response to first-, second- and third-line therapies more accurately than baseline profiling alone [1616,1617,1618,1619]. For instance, dynamic monitoring of ctDNA aids in prognosis, notably in first-line bevacizumab-based chemotherapy for mCRC [1616]. It has also been found to accurately reflect tumor burden and predict metastasis-free (PFS) survival in patients with locally advanced rectal cancer (LARC) undergoing neoadjuvant chemoradiotherapy (nCRT) [1617]. In LARC patients, the combination of ctDNA and MRI has been shown to improve the prediction of pathological complete response (pCR) and recurrence risk [1618]. Furthermore, early changes in ctDNA concentration have been linked to therapeutic efficacy in mCRC patients [1619]. Such findings underscore the importance of utilizing plasma ctDNA analysis for mutation profiling in mCRC patients, offering a less invasive alternative to traditional tissue biopsy methods [1607,1608].

#### Leveraging Computational Modeling and Machine Learning for CRC Personalized Therapy

As the armamentarium of targeted drugs and combinations expands rapidly, high-throughput profiling technologies will become essential for parsing patient subgroups [1620,1621]. Next-generation sequencing technologies now allow comprehensive molecular profiling of tumor samples through multiple omics lenses simultaneously [1621,1622,1623]. For instance, panel-based or targeted sequencing tests focus on genes associated with particularly rare diseases, facilitating their causal mutation detection [1621,1622,1623]. These tests have revealed a range of mutations in CRC, including in genes such as *APC*, *MLH1*, *MSH2*, *MSH6*, *PMS2*, *MUTYH*, *NTHL1*, *KRAS*, *TP53*, *FBXW7*, *PIK3CA*, *BRAF*, *CTNNB1*, *ERBB2*, and *SMAD4* [1621,1622,1623]. Furthermore, a cost-effective DNA pooling next-generation sequencing (NGS) strategy has been employed to identify rare single nucleotide variants and small indels in established and candidate CRC susceptibility genes [1624]. Accordingly, initiatives like OCTANE utilize NGS to unify molecular profiling approaches in cancer care [1625]. It facilitates identification of oncogenic driver mutations for targeted drug treatment through integration of whole-genome tumor sequencing (WGTS) with immune profiling [1625]. Collectively, NGS provides an unprecedented view of the genomic, epigenomic and proteomic alterations present in each patient’s unique cancer [1620,1621]. Combining these diverse data types using multi-omics analytical approaches has the potential to uncover novel biomarkers composed of sets of molecular features that more accurately predict response or resistance to therapeutic combinations [1604,1626,1627]. Rather than relying on single genes or pathways, these composite biomarkers may capture the multi-factorial determinants of treatment outcome in complex cancers like CRC [1604,1626,1627].

Computational modeling provides a way to simulate the complex dynamic interplay of pathways that gives rise to plasticity like the influence of the tumor microenvironment and the crosstalk between cancer cells and macrophages [1628,1629]. These models aid in mechanistically understanding complex biological systems, facilitating insight into cellular signaling dynamics [1630]. They are crucial for analyzing the operation of biochemical networks, including cell signaling pathways [1631]. Mathematical modeling assesses the dynamics and robustness of regulatory networks, offering a comprehensive approach to studying cellular behavior [1631,1632]. Moreover, computational models elucidate the interplay between extracellular matrix and signaling networks, shedding light on regulatory mechanisms [1633]. In addition, these modeling allows for the simulation of dynamic pathways, aiding in the identification of critical nodes, bypass routes, and activation patterns for adaptive survival [1634]. This allows in silico experimentation to identify critical nodes, bypass routes, and sequential activation patterns involved in adaptive survival [1635]. Simulations may predict currently unknown effectors or routes that emerge as compensatory strategies [1636]. Computational models integrate mathematical equations with computational resources, aiding in the investigation of biological mechanisms [1636].

An important application of modeling is optimizing drug orderings to limit pathway flux rerouting. By simulating pathway responses to sequential versus concurrent therapies, models may determine how to progressively block shifting signaling conduits over time. Araujo et al. (2005) and Saez-Rodriguez et al. (2015) both highlight the potential of computational models in understanding and optimizing cancer therapies [1514,1637]. Araujo’s work specifically demonstrates the enhanced attenuation of biochemical signals when multiple upstream processes are inhibited, particularly in serially-connected target points [1514]. This finding suggests that sequential therapies, which progressively block shifting signaling conduits over time, may be more effective than concurrent therapies [1514]. Eduati et al. (2020) further supports this idea by using patient-specific logic models to predict personalized combination therapies, indicating the potential for tailored sequential treatments [1638]. Calder et al. (2006) also contributes to this discussion by emphasizing the importance of accurate computational modeling in simulating pathway responses to different therapeutic strategies [1639]. These findings and similar reports shed a light on this debate that how computational approaches and molecular pathway data integration could improve researchers’ insights into drug responses and optimize therapeutic outcomes [1640].

Computational models can also evaluate factors like treatment schedules, durations, and combinations to identify protocols maximally disabling long-term pathway plasticity [1641,1642]. As dynamic omics data from clinical trials of rational sequencing regimens becomes available, models can incorporate new biological insights to iteratively improve predicted guidance strategies [1641]. In this regard, a recent computational model has been developed to quantify the global effects of mutations and drug treatments in the signaling networks of CRC cells [1642]. This model, based on a chemical reaction network, can simulate the impact of single or multiple mutations on protein concentrations and identify potential therapeutic targets [1642]. The model was further extended to account for the effects of targeted drugs, demonstrating its potential in personalized medicine [1642]. This work complements Roumeliotis et al. (2017), who used isobaric labeling to characterize the proteomic landscapes of CRC cell lines and identify the functional consequences of somatic genomic variants [1643]. Accordingly, new modeling approaches, like those developed at the University of Illinois Urbana-Champaign, provide insights into understanding colon cancer, including its long-term effects and responses to treatment protocols [1644]. Ultimately, computational approaches aim to give physicians a roadmap visualizing how best to guide CRC signaling networks toward a state where adaptive survival capacity is most constrained [1641,1645]. By simulating plasticity network-wide rather than focusing on isolated nodes or routes, these emerging techniques may revolutionize strategies to pre-emptively intercept tumor adaptation [1641,1645].

Applying machine learning (ML) to the huge volumes of integrated multi-omics data being generated by clinical trials offers a way to discover extremely complex predictive models [1646]. Algorithms can train on datasets containing molecular profiles paired with patient treatment exposures and clinical course [1647]. Techniques such as dimensionality reduction, autoencoders, random forests, and support vector machines are commonly used to handle the high feature count and relatively few samples in these datasets [1648]. Bayesian models, tree-based methods, kernel methods, network-based fusion methods, and matrix factorization models have also been explored for integrating multi-view biological data in machine learning systems [1649]. This allows the software to autonomously identify intricate response-predictive patterns spanning multiple levels of biological information [1647]. The models developed through deep learning may far surpass human ability to detect subtle inter-related predictive signatures [1647,1650]. As clinical trial datasets accumulate over time with additional patient–treatment–outcome trios, the predictive performance of these data-driven machine learning biomarkers is expected to steadily improve [1650].

A range of studies have demonstrated the potential of ML in predicting clinical outcomes in CRC [1651,1652,1653,1654]. Gründner et al. (2018) and Chowdary et al. (2023) both highlight the success of ML models in predicting disease-free survival, relapse, and response to radio-chemotherapy, with accuracies ranging from 0.70 to 0.86 [1651,1652]. These models have the potential to aid in decision-making and improve survival prognosis [1651,1652]. Alboaneen et al. (2023) further emphasizes the benefits of ML and deep learning in early diagnosis, particularly in the analysis of medical texts and images [1653]. Achilonu et al. (2021) extends this research to the South African population, demonstrating the high discriminative accuracies of ML algorithms in predicting CRC recurrence and patient survival [1654]. Together, these studies underscore the potential of ML in leveraging multi-omics data to improve clinical decision-making in clinical outcomes in CRC [1651,1652,1653,1654].

In summary, adoption of multi-omics profiling and machine learning approaches has the potential to truly transform cancer treatment by enabling rational upfront selection of optimal personalized combination therapies [1626,1655]. By precisely pairing each patient to the specific regimens their tumor biology profile indicates they have the highest chance of benefiting from, these advances promise to minimize exposure to ineffective toxic treatments [1656]. This personalized precision oncology approach aims to optimize clinical benefit for CRC patients in the era of expanding combination options [1657,1658,1659,1660].

## 6. Conclusions

Taken together, this extensive review presents a comprehensive perspective on the multifaceted challenges posed by CRC, a formidable global health burden. The review sheds light on the distinct clinical and molecular features that distinguished colon from rectal cancers, emphasizing the critical role of tumor location in guiding treatment. It elucidates the intricate architecture of colonic crypts and their vital functions in intestinal homeostasis. Unraveling the complex pathways of carcinogenesis, this review navigates the conventional adenoma–carcinoma sequence, the serrated neoplasia route, and colitis-associated cancer. The influential Vogelstein model, proposing sequential *APC*, *KRAS*, and *TP53* alterations as drivers, was extensively explored. Notably, the review spotlighted the CMS1–CMS4 molecular subtypes, capturing disease heterogeneity and guiding personalized approaches.

Ultimately, this comprehensive review synthesized the current knowledge while highlighting the invaluable contributions of experimental models, from mouse models to organoids and xenografts. These powerful tools dissected CRC’s intricate molecular landscapes, accelerating the quest for novel targets and strategies. As researchers continue unraveling this disease’s complexities, this review beacons future endeavors toward improving outcomes and more effectively managing this formidable challenge.

## Figures and Tables

**Table 1:** GLOBOCAN estimates incidence and mortality worldwide of colorectal cancer [3].

World Region	Incidence Rate (ASR per 100,000)	Number of New Cases	Mortality Rate (ASR per 100,000)	Number of Deaths
**Africa**	8.2	70,428	5.6	46,087
**Asia**	15.6	966,399	7.1	462,252
**Europe**	30.5	538,262	12.1	247,842
**Latin America and Caribbean**	16.9	145,120	8.2	73,647
**Northern America**	27.2	183,973	8.2	66,155
**Oceania**	31.1	22,243	9.2	8036
**Total**	18.4	1,926,425	8.1	904,019

ASR: Age-Specific Rates.

**Table 2:** Summarizing the prevalence, molecular characteristics, and clinical outcomes of the four consensus molecular subtypes in colorectal cancer [148,149].

Consensus Molecular Subtype	Prevalence (%)	Molecular Characteristics	Clinical Outcomes
**CMS1 (MSI Immune)**	14	High Microsatellite Instability Strong Immune Cell Infiltration	Best Prognosis Responsive to Immune Checkpoint Blockade
**CMS2 (Canonical)**	37	Activation of Wnt and Myc Signaling Pathways Chromosomal Instability	Intermediate Prognosis
**CMS3 (Metabolic)**	13	Dysregulation of Metabolic Pathways Obesity-Related Molecular Alterations	Poor Prognosis Resistance to Standard 5-FU Chemotherapy
**CMS4 (Mesenchymal)**	23	Activation of TGF-b and EMT Transcriptional Programs Stromal Infiltration	Poorest Prognosis Aggressive Clinical Course

**CMS**: consensus molecular subtype; **MSI**: microsatellite instability; **TGF**: tumor growth factor; **EMT**: epithelial-to-mesenchymal transition.

**Table 3:** Summary of clinical trials investigating the combination therapeutic strategies in CRC.

Strategy	Clinical Identifier	Combination	Setting	Primary Objective	Clinical Trial Phase	Sample Size	ORR (%)	PFS (Months)	OS (Months)	Reference
Bispecific antibodies	NCT02650713	RO6958688	Refractory	To assess the safety, tolerability, pharmacokinetics, pharmacodynamics, and preliminary efficacy of CEA-CD3 TCB (RO6958688) monotherapy in CRC participants	I	38	12	NA	NA	[1525]
	NCT02324257	RO6958688 + Atezolizumab	Refractory	To assess the safety, tolerability, pharmacokinetics, pharmacodynamics, and preliminary efficacy of CEA-CD3 TCB (RO6958688) combined with atezolizumab in CRC participants	I	68	6	NA	NA	[1525]
	NCT04826003	RO7122290 + Cibisatamab + Obinutuzumab	Refractory	To assess the safety, pharmacokinetics, pharmacodynamics, and preliminary antitumor activity of RO7122290 in combination with Cibisatamab for mCRC	I	Recruiting	NA	NA	NA	Still ongoing
	NCT04468607	BLYG8824A	Refractory	To assess the safety and efficacy of BLYG8824A in treating locally advanced colorectal cancer	I	Recruiting	NA	NA	NA	Still ongoing
BRAF + MEK and EGFR inhibitors	NCT02928224	Triplet (Encorafenib + Binimetinib + Cetuximab) vs. Doublet (Encorafenib + Cetuximab) and Control	Refractory	To study the combination of encorafenib and cetuximab with or without binimetinib for the treatment of mCRC	III	224 vs. 220 and 221	26.8 vs. 19.5 and 1.8	4.5 vs. 4.3 and 1.5	9.3 vs. 9.3 and 5.9	[1526]
BRAF + MEK inhibitors and ICI	NCT03668431	Dabrafenib + Trametinib + Spartalizumab/PDR001	First-line	To provide insight into the combining the PD-1, BRAF, and MEK inhibition in treatment of patients with BRAFV600E-mutated CRC	II	37	24.3	4.3	13.6	[1527]
EGFR Inhibitors	NCT01412957	Panitumumab + BSC vs. BSC alone	Refractory	To evaluate the survival benefit of panitumumab in combination with supportive care compared to supportive care alone in patients with mCRC	III	189 vs. 188	27 vs. 1.6	3.6 vs. 1.7	10 vs. 7.4	[1528]
	NCT01001377	Cetuximab vs. Panitumumab	Refractory	To compare cetuximab and panitumumab in terms of effectiveness and safety in mCRC patients	III	504 vs. 506	19.79 vs. 22.02	4.4 vs. 4.1	10 vs. 10.4	[1529]
	NCT00113763	Panitumumab (ABX-EGF) plus BSC vs. BSC alone	Refractory	To evaluate the impact of ABX-EGF plus best supportive care versus best supportive care alone in patients with mCRC	III	231 vs. 232	NA	2 vs. 1.8	6.4 vs. 6.3	[1530]
EGFR Inhibitor + Chemotherapy	NCT02394795	Panitumumab + chemotherapy vs. Bevacizumab + chemotherapy	First-line	To evaluate the efficacy of panitumumab versus bevacizumab when added to standard first-line chemotherapy for CRC	III	400 vs. 402	74.9 vs. 67.3	12.2 vs. 11.4	36.2 vs. 31.3	[1531]
	NCT01228734	Cetuximab + FOLFOX4 vs. FOLFOX4 alone	First-line	To compare Cetuximab + FOLFOX4 efficacy with FOLFOX4 alone in patients with previously untreated mCRC	III	193 vs. 204	61.1 vs. 39.5	9.2 vs. 7.4	20.7 vs. 17.8	[1532]
	NCT00339183	Panitumumab + FOLFIRI vs. FOLFIRI alone	Refractory	To evaluate the combination of panitumumab with FOLFIRI chemotherapy compared to FOLFIRI alone for patients with mCRC	III	591 vs. 595	35 vs. 10	5.9 vs. 3.9	14.5 vs. 12.5	[1533]
	NCT00154102	Cetuximab + FOLFIRI vs. FOLFIRI alone	First-line	To study the efficacy of cetuximab plus FOLFIRI as treatment for mCRC	III	599 vs. 599	46.9 vs. 38.7	8.9 vs. 8	19.9 vs. 18.6	[1534]
	NCT00364013	FOLFOX + Panitumumab vs. FOLFOX	First-line	To assess panitumumab with FOLFOX4 efficacy in mCRC patients	III	325 (Wild-type *KRAS*) and 221 (Mutant *KRAS*) vs. 331 (Wild-type *KRAS*) and 219 (Mutant *KRAS*)	NA	9.6 and 7.3 vs. 8 and 8.8	23.9 and 15.5 vs. 19.7 and 19.3	[1535]
EGFR inhibitors + ICI	NCT03442569	Panitumumab + Ipilimumab + Nivolumab	Refractory	To investigate the combination of nivolumab and ipilimumab with panitumumab for patients with unresectable, refractory, *KRAS*/NRAS mutant CRC	II	56	35	5.7	NR	[1522]
	NCT03608046	Cetuximab + Avelumab	Refractory	To evaluate the efficacy of avelumab combined with cetuximab and irinotecan for treating microsatellite stable mCRC patients	I/II	10 (RAS WT) vs. 13 (RAS mutant)	30 vs. 0	6 vs. 3.4	13.7 vs. 7.9	[1536]
	NCT04561336	Cetuximab + Avelumab	Refractory	To test the combination of avelumab and cetuximab for mCRC patients with RAS wild-type tumors	II	77	8.5	3.6	11.6	[1521]
	NCT04017650	Encorafenib + Cetuximab + Nivolumab	Refractory	To check the efficacy and safety of the treatment combination in mCRC patients, particularly those with BRAF V600E mutation	I/II	26	45	7.3	11.4	[1537]
EGFR Inhibitor + Immunotherapy + Chemotherapy	NCT03174405	Cetuximab + Avelumab + FOLFOX	First-line	To examine the combination of avelumab and cetuximab with FOLFOX chemotherapy in patients with metastatic colorectal cancer.	II	43	NA	11.1	32.9	[1538]
ICI + MEK Inhibitor + MKI		Atezolizumab Monotherapy vs. Atezolizumab + Cobimetinib, and vs. Regorafenib	Refractory	To investigate the efficacy and safety of cobimetinib in combination with atezolizumab versus regorafenib for participants with CRC	III	121 vs. 61, and vs. 61	2.2 vs. 2.7, and vs. 2.2	1.5 (atezolizumab + cobimetinib vs. regorafenib) and 1.39 (atezolizumab monotherapy vs. regorafenib)	8.5 vs. 8.9 and vs. 7.1	[1539]
ICI + Chemotherapy	NCT03202758	Durvalumab + Tremelimumab + mFOLFOX6	First-line	To evaluate the safety and efficacy of Durvalumab and tremelimumab in combination with FOLFOX chemotherapy regimen for patients with previously untreated mCRC	Ib/II	57	64.5	8.2	NA	[1540]
	NCT03832621	Ipilimumab + Nivolumab + Temozolomide	Refractory	To provide insights into the efficacy and safety of combining Temozolomide (TMZ) with other agents for treating mCRC	II	33	45	7	18.4	[1541]
	NCT02563002	Pembrolizumab vs. chemotherapy (mFOLFOX6 or FOLFIRI with or without bevacizumab or cetuximab)	First-line	To standard therapy for MSI-H advanced CRC	III	153 vs. 154	42 vs. 33	16.5 vs. 8.2	NA vs. 36.7	[1542]
ICI + Vaccines	NCT01413295	Avelumab + ADC vaccine	Refractory	To evaluate the combination of avelumab (anti-PD-L1) plus autologous dendritic cell (ADC) vaccine in pre-treated mismatch repair-proficient mCRC	I/II	19	0	3.1	12.2	[1543]
	NCT03050814	mFOLFOX6 + Bevacizumab (standard of care, SOC) alone or with Avelumab immunotherapy and AdCEA vaccine (SOC + IO)		To Study the combination of mFOLFOX6 + bevacizumab alone or with avelumab immunotherapy and AdCEA vaccine in mCRC patients	II	10 vs. 10	50 vs. 50	8.8 vs. 10.1	NR	[1544]
ICI + Vaccines + Chemotherapy	NCT00529984	Pembrolizumab + Adoptive cell therapy (ACT) + Cyclophosphamide + Fludarabine	Refractory	To test the CEA(6D) VRP vaccine’s safety in patients with advanced or metastatic CRC conditions	I/II	21	NA	NA	NA	[1545]
	NCT02981524	Pembrolizumab (anti-PD-1) + GVAX colon vaccine + low dose Cyclophosphamide	Refractory	To investigate the efficacy of GVAX colon vaccine combined with pembrolizumab in patients with advanced MMR-p CRC	II	17	0	0.82	7.1	[1546]
Immunotherapy	NCT02060188	Pembrolizumab	Refractory	To investigate the efficacy of pembrolizumab in patients with advanced solid tumors that are deficient in mismatch repair (MMR), regardless of tissue of origin	II	18	0	2.2	5	[1547]
	NCT02870920	Durvalumab + Tremelimumab + BSC vs. BSC alone	Refractory	To evaluate the combination of durvalumab (PD-L1 inhibitor) and tremelimumab (CTLA-4 inhibitor) vs. best supportive care alone in patients with advanced CRC	II	119 vs. 61	1 vs. 0	1.8 vs. 1.9	6.6 vs. 4.1	[1548]
Immunotherapy + Radiotherapy	NCT02888743	Duvalumab (PD-L1) + Tremelimumab (CTLA-4) + Low-dose Fractionated Radiotherapy or Hypofractionated Radiotherapy	Refractory	To test the combination of PD-L1/CTLA-4 inhibition with LDFRT or HFRT for patients with microsatellite-stable mCRC	II	10 vs. 10	0 vs. 0	1.7 vs. 1.8	3.5 vs. 4	[1549]
	NCT03104439	Ipilimumab (anti-CTLA4 antibody) + Nivolumab (anti-PD1 antibody) + Radiation therapy	Refractory	To evaluate the efficacy of combining radiation therapy with ipilimumab and nivolumab in treating microsatellite stable CRC patients	II	40	10	2.4	7.1	[1550]
Immunotherapy + Vaccines	NCT00154713	Interleukin-2 (IL-2) + DC-based cancer vaccine	Refractory	To test the immunotherapy’s efficacy using dendritic cells (DCs) pulsed with carcinoembryonic antigen (CEA) and tetanus toxoid, followed by interleukin-2 (IL-2) treatment in mCRC	I	12	NA	NA	NA	[1365]
Interleukin-1α (IL-1α) neutraliser	NCT01767857	Xilonix (MABp1) vs. Placebo	Refractory	To evaluate MABp1 treatment impact for advanced CRC	III	411 vs. 200	NA	2.1 vs. 2.1	5.6 vs. 5.4	[1551]
MEK Inhibitor + ICI	NCT02788279	Regorafenib vs. Cobimetinib + Atezolizumab vs. Atezolizumab	Refractory	To investigate the efficacy and safety of target drugs for participants with CRC	III	90 vs. 183 and 90	NA	2 vs. 1.91 and 1.94	8.51 vs. 8.87 and 7.1	[1552]
Nucleoside Metabolic Inhibitor	NCT01607957	TAS-102 (Trifluridine/Tipiracil) vs. Placebo	Refractory	To assess the efficacy and safety of TAS-102 in treating refractory mCRC	III	534 vs. 266	1.6 vs. 0.4	2 vs. 1.7	7.1 vs. 5.3	[1553]
	NCT01955837	TAS-102 vs. Placebo	Refractory	To evaluate the efficacy and safety of combining trifluridine/tipiracil in Asian patients with mCRC	III	271 vs. 135	1.1 vs. 0	2 vs. 1.8	7.8 vs. 7.1	[1554]
TKIs	NCT00700102	Avastin (Bevacizumab) plus chemotherapy vs. chemotherapy alone	Refractory	To assess the efficacy of Avastin combined with crossover fluoropyrimidine-based chemotherapy in patients with mCRC	III	409 vs. 411	NA	5.7 vs. 4.1	11.2 vs. 9.8	[1555]
	NCT04322539	Fruquintinib (HMPL-013) + BSC Group vs. Placebo + BSC Group	Refractory	Evaluate the efficacy and safety of HMPL-013 in patients mCRC	III	461 vs. 230	1.5	3.7 vs. 1.8	7.4 vs. 4.8	[1556]
	NCT01103323	Regorafenib +BSC vs. Placebo + BSC	Refractory	Assessing regorafenib’s efficacy and safety in mCRC	III	505 vs. 255	1.5	1.9 vs. 1.7	6.4 vs. 5	[1557]
	NCT02314819	Fruquintinib + BSC vs. placebo + BSC	Refractory	To evaluate the efficacy and safety of fruquintinib in patients mCRC	III	278 vs. 138	20.9 vs. 4.3	3.71 vs. 1.84	9.3 vs. 6.57	[1558]
TKI + ICI	NCT03406871	Regorafenib + Nivolumab	Refractory	To evaluate the combination of regorafenib plus nivolumab for gastric and colorectal cancers.	IB	25	36	79	NA	[1559]
	NCT03657641	Regorafenib + Pembrolizumab	Refractory	To examine the combination of regorafenib and pembrolizumab in patients with advanced MSS-CRC	I/II	73	0	2	10.9	[1560]
	NCT04362839	Regorafenib + Ipilimumab + Nivolumab (RIN)	Refractory	To assess the combination of regorafenib, ipilimumab, and nivolumab (RIN) for chemotherapy-resistant MSS-mCRC	I/II	39	36	5	22	[1561]
	NCT03539822	Cabometyx (Cabo) + Durvalumab (Durva)	Refractory	To test the combination of cabometyx (Cabo) + durvalumab (Durva) for advanced pMMR/MSS-CRC patients	II	36	27.6	4.4	9.1	[1562]
	NCT03332498	Ibrutinib + Pembrolizumab	Refractory	To evaluate the efficacy and safety of pembrolizumab in combination with ibrutinib for treating advanced CRC	I/II	40	0	1.4	6.6	[1563]
	NCT03712943	Regorafenib + Nivolumab	Refractory	To investigate the combination of regorafenib and nivolumab in patients with pMMR-CRC	I/Ib	52	10	4.3	11.1	[1564]
	NCT03797326	Lenvatinib + Pembrolizumab	Refractory	To examine the efficacy and safety of combining lenvatinib and pembrolizumab in patients with non-MSI-H/pMMR	II	32	22	2.3	7.5	[1565]
	NCT04126733	Regorafenib + Nivolumab	Refractory	To evaluate the combination of regorafenib plus nivolumab in patients with dMMR/MSI-H CRC	II	70	7	1.8	11.9	[1566]
	NCT03170960	Cabozantinib + Atezolizumab	Refractory	To test the combination of cabozantinib with atezolizumab in patients with previously treated mCRC	Ib	31	9.7	3	14	[1567]
Vaccines	NCT01147965	Ad5 [E1-, E2b-]-CEA(6D) vaccine	Refractory	To evaluate ETBX-011, a therapeutic vaccine, in adults with CEA-expressing advanced or mCRC	I/II	32	NR	NR	48	[1568]
	NCT01890213	AVX701	NA	To determine the use of virus-like replicon particles (VRP) delivering antigens against stage III CRC	I	12	NA	NA	NA	[1569]
	NCT01462513	Tecemotide (L-BLP25) vs. Placebo	Adjuvant/post-operative	To investigate adjuvant immunotherapy with tecemotide (L-BLP25) after R0/R1 resection in CRC patients	II	79 vs. 42	NR	6.1 vs. 11.4	62.8 vs. NR	[1570]
	NCT01461148	The FSP vaccine included peptides derived from three genes: AIM2, HT001, and TAF1B.	Refractory	To assess the safety and immunogenicity of a frameshift peptide (FSP)-based vaccine for MSI-H CRC.	I/IIa	22	NR	NR	NR	[1571]
Vaccines + Chemotherapy	UMIN000001791	Five HLA-A2402-restricted peptide cocktail + mFOLFOX6 or XELOX	Refractory	To assess the efficacy and safety of combining five HLA-A2402-restricted peptide cocktail (derived from RNF43, TOMM34, KOC1, VEGFR1, VEGFR2) in combination with oxaliplatin-based chemotherapy as a first-line therapy for advanced CRC	II	50 (HLA-A * 2402-matched) vs. 46 (HLA-A * 2402-unmatched)	62 vs. 60.9	7.2 vs. 8.7	20.7 vs. 24	[1572]
VEGF Inhibitor + Chemotherapy	NCT00561470	Aflibercept + FOLFIRI vs. Placebo + FOLFIRI	Refractory	To evaluate the efficacy and safety of aflibercept in combination with irinotecan and fluorouracil in treating patients with mCRC	III	614 vs. 612	19.8 vs. 11.1	6.9 vs. 4.67	13.5 vs. 12.06	[1573]
	NCT01183780	Ramucirumab + FOLFIRI	Refractory	To check the efficacy and safety of ramucirumab plus FOLFIRI compared to placebo plus FOLFIRI as second-line treatment for in mCRC patients whose disease had progressed during or after first-line treatment including bevacizumab.	III	536 vs. 536	13.4 vs. 12.5	5.7 vs. 4.5	13.3 vs. 11.7	[1574]
	NCT01661270	Aflibercept + FOLFIRI vs. FOLFIRI + Placebo	Refractory	To investigate aflibercept in combination with FOLFIRI in patients with mCRC who had progressed on a prior oxaliplatin regimen	III	223 vs. 109	18.4 vs. 3.7	6.93 vs. 5.59	14.59 vs. 11.93	[1575]
VEGF Inhibitor + ICI + Chemotherapy	NCT03721653	FOLFOXIRI + bevacizumab + atezolizumab vs. FOLFOXIRI + bevacizumab	First-line	To investigate upfront FOLFOXIRI plus bevacizumab and atezolizumab for unresectable mCRC	II	73 vs. 145	NR	13.1 vs. 11.5	NR	[1576]
	NCT02873195	Atezolizumab + Capecitabine + Bevacizumab vs. Capecitabine +Bevacizumab	Refractory	To study the efficacy of capecitabine and bevacizumab with or without atezolizumab in treating CRC patients	II	82 vs. 46	8.5 vs. 4.4	4.4 vs. 3.6	10.3 vs. 10.2	[1577]
VEGF Inhibitor + Nucleoside Metabolic Inhibitor + Chemotherapy	NCT03750786	ARFOX + Bevacizumab vs. mFOLFOX-6 + Bevacizumab	First-line	To compare the efficacy of arfolitixorin versus leucovorin in patients with mCRC	III	245 vs. 245	48.2 vs. 49.4	12.8 vs. 11.6	23.8 vs. 28	[1578]
